# Species of Dactylogyridae (Platyhelminthes: Monogenoidea) infecting marine fishes of Moreton Bay, Queensland, Australia, with proposals of *Pleuronectitrema* n. gen. and *Ecnomotrema* n. gen. and descriptions of 13 new species

**DOI:** 10.1051/parasite/2023053

**Published:** 2023-12-19

**Authors:** Delane C. Kritsky

**Affiliations:** School of Health Professions, Campus Box 8090, Idaho State University Pocatello Idaho 83209 USA

**Keywords:** Dactylogyrids, Monogenoidea, “Monogenea”, Parasite diversity, Taxonomy, New species, New genera, Moreton Bay

## Abstract

Twenty-nine dactylogyrid species were reported from teleosts collected during a survey of the parasites of fishes of Moreton Bay, Queensland, Australia, in 2016. Two new genera, *Ecnomotrema* n. gen. and *Pleuronectitrema* n. gen., were proposed, and 13 new species were illustrated and described: *Atherinicus difficilis* n. sp., *Diversohamulus similis* n. sp., and *Ecnomotrema cetiosum* n. sp. from *Atherinomorus vaigiensis*; *Haliotrema apiculum* n. sp. from *Pempheris schwenkii*; *Haliotrema tugulduriforme* n. sp. from *Scarus ghobban*; *Lethrinitrema australiense* n. sp., and *Lethrinitrema lituus* n. sp. from *Lethrinus nebulosus*; *Tetrancistrum siganioides* n. sp. from *Siganus fuscescens*; *Ligophorus bostrychus* n. sp. from *Planiliza subviridis*; *Neohaliotrema gemmula* n. sp. from *Abudefduf vaigiensis*; *Neohaliotrema moretonense* n. sp. from *Ab. vaigiensis* and *Ab. bengalensis*; and *Pleuronectitrema spirula* n. sp. from *Pseudorhombus arsius* (all from Moreton Bay); *Pleuronectitrema kuwaitense* Kritsky & Sey n. sp. was described from specimens collected from *P. arsius* in Kuwait during 1996. Ten new host records were recorded: *Chauhanellus duriensis* Lim, 1994 and *Hamatopeduncularia thalassini* Bychowsky & Nagibina, 1969 from *Pararius proximus*; *Diplectanotrema* sp. 1 and sp. 2 from Sillago *maculata* and *Goniistius vestitus*, respectively; *Diversohamulus tricuspidatus* Bychowsky & Nagibina, 1969 from *At. vaigiensis*; *Hal.* cf. *dempsteri* (Mizelle & Price, 1964) Young, 1968 from *Prionurus microlepidotus*; *Hal. spirale* Yamaguti, 1968 from *Upeneus tragula*; *Ligophorus kaohsianghsieni* (Gussev, 1962) Gussev, 1985 from *Planiliza subviridis*; and *Neohaliotrema malayense* Lim & Gibson, 2010 from *Ab. bengalensis* and *Ab. whitleyi*. Twenty-five new faunal records for Moreton Bay were recorded, including the new species listed above and *C*. *duriensis*, *Diplectanotrema* sp. 1 and sp. 2, *Diversohamulus tricuspidatus* Bychowsky & Nagibina, 1969, *Glyphidohaptor phractophallus* Kritsky, Galli, & Yang, 2007, *Hal.* cf. *dempsteri*, *Hal. johnstoni* Bychowsky & Nagibina, 1970, *Hal. spirale*, Yamaguti, 1968 *Hamatopeduncularia thalassini* Bychowsky & Nagibina, 1969, *Lethrinitrema nebulosum* Sun, Li, & Yang, 2014, *Ligophorus kaohsianghsieni* (Gussev, 1962) Gussev, 1985, *Ligophorus parvicopulatrix* Soo & Lim, 2012, and *Neohaliotrema malayense* Lim & Gibson, 2010. Three new combinations were proposed: *Hal. spariense* Roubal, 1981 as *Euryhaliotrema spariense* (Roubal, 1981) n. comb.; and *Hal. arsiosa* Venkatanarasaiah, 1984 and *Hal. youngi* Venkatanarasaiah, 1984 as *Pleuronectitrema arsiosa* (Venkatanarasaiah, 1984) n. comb. and *Pleuronectitrema youngi* (Venkatanarasaiah, 1984) n. comb., respectively; *Haliotrema* sp. of Zhang is transferred to *Pleuronectitrema* as *Pleuronectitrema* sp. *Hal. ctenochaeti* Yamaguti, 1968 was replaced with *Hal. asymphylum* n. nom. to remove it from homonymy with *Hal. ctenochaeti* Young, 1968. *Pseudohaliotrematoides zancli* Yamaguti, 1968 was transferred to *Haliotrema* as *Haliotrema hawaiiense* n. nom. *Hal. zancli* Yamaguti, 1968 was considered a junior subjective synonym of *Hal. dempsteri*.

## Abstract (fr)

Vingt-neuf espèces de Dactylogyridae sont signalées chez des téléostéens collectés lors d’une étude des parasites des poissons de Moreton Bay, Queensland, Australie, en 2016. Deux nouveaux genres, *Ecnomotrema* n. gen. et *Pleuronectitrema* n. gen., sont proposés, et 13 nouvelles espèces sont illustrées et décrites : *Atherinicus difficilis* n. sp., *Diversohamulus similis* n. sp., et *Ecnomotrema cetiosum* n. sp. d’*Atherinomorus vaigiensis* ; *Haliotrema apiculum* n. sp. de *Pempheris schwenkii* ; *Haliotrema tugulduriforme* n. sp. de *Scarus ghobban* ; *Lethrinitrema australiense* n. sp. et *Lethrinitrema lituus* n. sp. de *Lethrinus nebulosus*, *Tetrancistrum siganioides* n. sp. de *Siganus fuscescens* ; *Ligophorus bostrychus* n. sp. de *Planiliza subviridis* ; *Neohaliotrema gemmula* n. sp. d’*Abudefduf vaigiensis* ; *Neohaliotrema moretonense* n. sp. d’*Ab. vaigiensis* et *Ab. bengalensis* ; et *Pleuronectitrema spirula* n. sp. de *Pseudorhombus arsius* (tous de Moreton Bay). *Pleuronectitrema hawaiiense* Kritsky & Sey n. sp. est décrit à partir de spécimens collectés de *P. arsius* au Koweït en 1996. Dix nouvelles combinaisons parasites-hôtes sont répertoriées : *Chauhanellus duriensis* Lim, 1994 et *Hamatopeduncularia thalassini* Bychowsky & Nagibina, 1969 de *Pararius proximus* ; *Diplectanotrema* sp. 1 et sp. 2, respectivement de *Sillago maculata* et *Goniistius vestitus* ; *Diversohamulus tricuspidatus* Bychowsky & Nagibina, 1969 d’*At. vaigiensis* ; *Hal.* cf. *dempsteri* (Mizelle & Price, 1964) Young, 1968 de *Prionurus microlepidotus* ; *Hal. spirale* Yamaguti, 1968 d’*Upeneus tragula* ; *Ligophorus kaohsianghsieni* (Gussev, 1962) Gussev, 1985 de *Planiliza subviridis* ; et *Neohaliotrema malayense* Lim & Gibson, 2010 d’*Ab. bengalensis* et *Ab. whitleyi*. Vingt-cinq nouveaux signalements fauniques pour la baie Moreton sont rapportés, y compris les nouvelles espèces énumérées ci-dessus et *C. duriensis*, *Diplectanotrema* sp. 1 et sp. 2, *Diversohamulus tricuspidatus* Bychowsky & Nagibina, 1969, *Glyphidohaptor phractophallus* Kritsky, Galli et Yang, 2007, *Hal.* cf. *dempsteri*, *Hal. johnstoni* Bychowsky et Nagibina, 1970, *Hal. spirale*, *Hamatopeduncularia thalassini* Bychowsky & Nagibina, 1969, *Lethrinitrema nebulosum* Sun, Li, & Yang, 2014, *Ligophorus kaohsianghsieni* (Gussev, 1962) Gussev, 1985, *Ligophorus parvicopulatrix* Soo & Lim, 2012, et *Neohaliotrema malayense* Lim et Gibson, 2010. Trois nouvelles combinaisons sont proposées : *Hal. spariense* Roubal, 1981 comme *Euryhaliotrema spariense* (Roubal, 1981) n. comb. ; *Hal. arsiosa* Venkatanarasaiah, 1984 et *Hal. youngi* Venkatanarasaiah, 1984, respectivement comme *Pleuronectitrema arsiosa* (Venkatanarasaiah, 1984) n. comb. et *Pleuronectitrema youngi* (Venkatanarasaiah, 1984) n. comb. ; *Haliotrema* sp. de Zhang est transféré à *Pleuronectitrema* sous le nom de *Pleuronectitrema* sp. *Hal. ctenochaeti* Yamaguti, 1968 est remplacé par *Hal. asymphylum* n. nom. pour supprimer l’homonymie avec *Hal. ctenochaeti* Young, 1968. *Pseudohaliotrematoides zancli* Yamaguti, 1968 est transféré à *Haliotrema* sous le nom de *Haliotrema hawaiiense* n. nom. *Hal. zancli* Yamaguti, 1968 est considéré comme un synonyme subjectif junior de *Hal. dempsteri*.

## Introduction

During 2016, the author participated in a comprehensive survey of the helminth parasites of the fishes of Moreton Bay, Queensland, Australia. Over a period of 12 days during January, the gill baskets of 278 marine fishes, representing 73 species, were excised and preserved for later study of the coexisting species of Monogenoidea. To date, 23 dactylogyrids [31–33, 41, 43], a gyrodactylid [32], a microcotylid [32], two bychowskicotylids [34], 18 monocotylids [10, 37], and a heteraxinid [42] have been recorded from the fishes collected during the survey.

Anticipated to be the final report on the dactylogyrids collected during the survey, an additional 29 species, including 12 new to science, are recorded from the bay in the present paper (Table 1). These findings and those of previous investigations demonstrate that the diversity of dactylogyrids, including potentially numerous other undescribed species in the bay, far exceeds that of all other monogenoidean families. Johnson [25] reported that a minimum of 1,190 species of marine fishes comprise the fish fauna of the Moreton Bay Marine Park, which suggests that the diversity of dactylogyrid species in the bay and surrounding area far exceeds that currently known.

**Table 1:** Dactylogyrid species known to occur on the marine fishes of Moreton Bay, Queensland, Australia.

Parent taxon	Species	Hosts*	References
*Atherinicus* Bychowsky & Nagibina, 1969	*A. difficilis* n. sp.**	*Atherinomorus vaigiensis* (Quoy & Gaimard)	[*ex nobis*]
*Chauhanellus* Bychowsky & Nagibina, 1969	*C. australis* (Young, 1967) Bychowsky & Nagibina, 1969	*Neoarius graeffei* (Kner & Steindachner)	[26]
	*C. duriensis* Lim, 1994*	*Pararius proximus* (Ogilby)***	[*ex nobis*]
	*C. youngi* Kearn & Whittington, 1994	*Neoarius graeffei* (Kner & Steindachner)	[26]
			
*Diplectanotrema* Johnston & Tiegs, 1922	*Diplectanotrema* sp. 1**	*Goniistius vestitus* (Castelnau)***	[*ex nobis*]
	*Diplectanotrema* sp. 2**	*Sillago maculata* Quoy & Gaimard***	[*ex nobis*]
*Diversohamulus* Bychowsky & Nagibina, 1969	*D. similis* n. sp.	*Atherinomorus vaigiensis* (Quoy & Gaimard)	[*ex nobis*]
	*D. tricuspidatus* Bychowsky & Nagibina, 1969**	*Atherinomorus vaigiensis* (Quoy & Gaimard)***	[*ex nobis*]
*Ecnomotrema* n. gen.	*E. cetiosum* n. sp.**	*Atherinomorus vaigiensis* (Quoy & Gaimard)	[*ex nobis*]
*Euryhaliotrema* Kritsky & Boeger, 2002	*E. solenophallus* Kritsky, 2019	*Monodactylus argenteus* (Linnaeus)	[33]
	*E. spariense* (Roubal, 1981) n. comb.	*Acanthopagrus australis* (Günther)	[8, *ex nobis*]
	*E. spirotubiforum* (Zhang, 2001) Wu, Zhu, Xie, & Li, 2006	*Lutjanus fulviflamma* (Forsskål)	[33]
		*Lutjanus russellii* (Bleeker)	[33]
*Glyphidohaptor* Kritsky, Galli, & Yang, 2007	*G. phractophallus* Kritsky, Galli, & Yang, 2007**	*Siganus fuscescens* (Houttuyn)	[*ex nobis*]
*Haliotrema* Johnston & Tiegs, 1922	*H. apiculum* n. sp.**	*Pempheris schwenkii* Bleeker	[*ex nobis*]
	*H.* cf. *dempsteri* (Mizelle & Price, 1964) Young, 1968**	*Prionurus microlepidotus* Lacepède***	[*ex nobis*]
	*H. lineati* Young, 1968	*Acanthurus lineatus* (Linnaeus)	[95]
	*H. johnstoni* Bychowsky & Nagibina, 1970**	*Upeneus tragula* Richardson	[*ex nobis*]
	*H. obesum* (Caballero, Bravo Hollis & Grocott, 1955) Young, 1968	*Arothron hispidus* (Linnaeus)	[41]
		*Arothron stellatus* (Anonymous)	[41]
	*H. spirale* Yamaguti, 1968**	*Upeneus tragula* Richardson***	[*ex nobis*]
	*H. tugulduriforme* n. sp.**	*Scarus ghobban* Fabricius	[*ex nobis*]
*Hamatopeduncularia* Yamaguti, 1953	*H. brisbanensis* Young, 1967	*Neoarius graeffei* (Kner & Steindachner)	[26, 93]
	*H. major* Kearn & Whittington, 1994	*Neoarius graeffei* (Kner & Steindachner)	[26]
	*H. pearsoni* Kearn & Whittington, 1994	*Neoarius graeffei* (Kner & Steindachner)	[26]
	*H. spiralis* Kearn & Whittington, 1994	*Neoarius graeffei* (Kner & Steindachner)	[26]
	*H. thalassini* Bychowsky & Nagibina, 1969**	*Pararius proximus* (Ogilby)***	[*ex nobis*]
*Hareocephalus* Young, 1968	*H. thaisae* Young, 1968	*Tylosurus gavialoides* (Castelnau)	[31, 96]
*Hemirhamphiculus* Bychowsky & Nagibina, 1969	*H. choanophallus* Kritsky, 2018	*Hyporhamphus regularis* (Günther)	[31]
	*H. exserocephalus* Kritsky, 2018	*Tylosurus gavialoides* (Castelnau)	[31]
	*H. flagrum* Kritsky, 2018	*Hyporhamphus regularis* (Günther)	[31]
	*H. incomptus* Kritsky, 2018	*Arrhamphus sclerolepis krefftii* (Steindachner)	[31]
	*H. krabsi* Kritsky, 2018	*Arrhamphus sclerolepis krefftii* (Steindachner)	[31]
	*H. perexiguus* Kritsky, 2018	*Arrhamphus sclerolepis krefftii* (Steindachner)	[31]
	*Hemirhamphiculus* sp. of Kritsky (2018)	*Hyporhamphus regularis* (Günther)	[31]
*Lethrinitrema* Lim & Justine, 2011	*L. australiense* n. sp.**	*Lethrinus nebulosus* (Forsskål)	[*ex nobis*]
	*L. fleti* (Young, 1968) Lim & Justine, 2011	*Lethrinus laticaudis* Alleyne & Macleay	[95, *ex nobis*]
		*Lethrinus miniatus* (Forster)	[95]
	*L. lituus* n. sp.**	*Lethrinus nebulosus* (Forsskål)	[*ex nobis*]
	*L. nebulosum* Sun, Li, & Yang, 2014**	*Lethrinus nebulosus* (Forsskål)	[*ex nobis*]
*Ligophorus* Euzet & Suriano, 1977	*L. bostrychus* n. sp.**	*Planiliza subviridis* (Valenciennes)	[*ex nobis*]
	*L. kaohsianghsieni* (Gussev, 1962) Gussev, 1985**	*Planiliza subviridis* (Valenciennes)***	[*ex nobis*]
	*L. parvicopulatrix* Soo & Lim, 2012**	*Planiliza subviridis* (Valenciennes)	[*ex nobis*]
*Metahaliotrema* Yamaguti, 1953	*M. filamentosum* Venkatanarasaiah, 1981	*Gerres oyena* (Fabricius)	[32]
		*Gerres subfasciatus* Cuvier	[32]
	*M. imparilis* Kritsky, 2018	*Gerres subfasciatus* Cuvier	[32]
*Neocalceostomoides* Kritsky, Mizelle, & Bilqees, 1978	*N. brisbanensis* Whittington & Kearn, 1995	*Neoarius graeffei* (Kner & Steindachner)	[27, 87]
*Neohaliotrema* Yamaguti, 1965	*N. gemmula* n. sp.**	*Abudefduf vaigiensis* (Quoy & Gaimard)	[*ex nobis*]
	*N. malayense* Lim & Gibson, 2010**	*Abudefduf vaigiensis* (Quoy & Gaimard)	[*ex nobis*]
		*Abudefduf bengalensis* (Bloch)***	[*ex nobis*]
		*Abudefduf whitleyi* Allen & Robertson***	[*ex nobis*]
	*N. moretonense* n. sp.**	*Abudefduf vaigiensis* (Quoy & Gaimard)	[*ex nobis*]
		*Abudefduf bengalensis* (Bloch)	[*ex nobis*]
*Parancylodiscoides* Caballero y C. & Bravo Hollis, 1961	*P. platacis* (Young, 1968) Kritsky, 2012)	*Platax pinnatus* (Linnaeus)	[96]
*Platycephalotrema* Kritsky & Nitta, 2019	*P. austrinum* Kritsky & Nitta, 2019	*Platycephalus endrachtensis* Quoy & Gaimard	[43]
	*P. koppa* Kritsky & Nitta, 2019	*Platycephalus fuscus* Cuvier	[43]
	*P. mastix* Kritsky & Nitta, 2019	*Platycephalus fuscus* Cuvier	[43]
	*P. platycephali* (Yin & Sproston, 1948) Kritsky & Nitta, 2019	*Platycephalus indicus* (Linnaeus)	[43]
		*Platycephalus fuscus* Cuvier	[43]
		*Platycephalus* sp.	[43]
	*P. thysanophrydis* (Yamaguti, 1965) Kritsky & Nitta, 2019	*Platycephalus fuscus* Cuvier	[43, 96]
		*Platycephalus endrachtensis* Quoy & Gaimard	[43]
*Pleuronectitrema* n. gen.	*P. spirula* n. sp.**	*Pseudorhombus arsius* (Hamilton)	[*ex nobis*]
*Protogyrodactylus* Johnston & Tiegs, 1922	*P. ichthyocercus* Kritsky, 2018	*Gerres oyena* (Forsskål)	[32]
	*P. monacanthus* Kritsky, 2018	*Gerres subfasciatus* Cuvier	[32]
	*P. scalmophorus* Kritsky, 2018	*Gerres oyena* (Forsskål)	[32]
	*P. similis* Kritsky, 2018	*Gerres oyena* (Forsskål)	[32]
	*P. vulgaris* Kritsky, 2018	*Gerres subfasciatus* Cuvier	[32]
*Tetrancistrum* Goto & Kikuchi, 1917	*T. sigani* Goto & Kikuchi, 1917	*Siganus fuscescens* (Houttuyn)	[*ex nobis*]
	*T. siganioides* n. sp.**	*Siganus fuscescens* (Houttuyn)	[*ex nobis*]
*Triacanthinella* Bychowsky & Nagibina, 1968	*T. falcanalis* (Young, 1968) Lim & Gibson, 2008	*Tripodichthys angustifrons* (Hollard)	[95, *ex nobis*]

* Names of hosts are as listed in Fricke *et al.* [14].

** New faunal records for Moreton Bay.

*** New host record.

Moreton Bay is situated off the southeastern tip of Queensland and represents the northern limit of the Peronian Marine Biogeographical Region of Australia. The bay was formed about 6,000 years ago from the rising sea level that inundated the flood plain of the Brisbane River of eastern Australia and is bounded to the east by Moreton and North and South Stradbroke Islands. The bay is considered subtropical, is generally shallow (predominantly about 20 m deep) with a sandy bottom, and harbors 753 species of fishes [25]. The bay and surrounding area are situated near the northern (64 species) and southern (310 species) limits of the natural distributions of many fish species [25], making for an uncommon and perhaps unique community of fishes and their parasites.

## Materials and methods

Fishes were collected from Moreton Bay, Queensland, Australia, from January 10 to 21, 2016. The fishes were transported alive to the Moreton Bay Research Station located in Dunwich, North Stradbroke Island, Queensland, where they were euthanized, identified using Johnson [25], and necropsied for parasitic infections. Common and scientific names of hosts were determined from Froese & Pauly [15] and verified in Fricke *et al.* [14], respectively. Methods for collection, preparation, illustration, and measurement of monogenoidean specimens collected in Moreton Bay were those of Kritsky [31]; see Sey & Nahhas [77] for methods for the collection and preservation of specimens from Kuwait. With the exception of *Diplectanotrema* spp., specimens of which were found only in the sediments of the vials holding the respective gill baskets, all other dactylogyrid specimens were found on the gills of their respective hosts as well as within sediments in the respective vials. Measurements, all in micrometers, represent straight-line distances between extreme points and are expressed as the mean followed by the range and number (*n*) of structures measured in parentheses; body length includes that of the haptor; the length of the copulatory complex or male copulatory organ (MCO) represents the distance between the two parallel lines depicted on the respective drawings for each species or were obtained (as total length) using a calibrated curvimeter on drawings; direction of the coil of the MCO, clockwise *vs.* counterclockwise, was determined using the convention proposed by Kritsky *et al.* [36]. Numbering (distribution) of haptoral hooks follows the convention proposed by Mizelle [57, see 59]. Descriptions of new species are based solely on designated type specimens, except fragments of dissected specimens mounted in Gray & Wess medium [20] were occasionally used to obtain drawings of haptoral and copulatory sclerites. All type and voucher specimens of helminths were deposited in the Queensland Museum (QM), Brisbane, Queensland, Australia; the Australian Museum (AM), Sydney, New South Wales, Australia; the Invertebrate Zoology Collection (USNM), National Museum of Natural History, Smithsonian Institution, Washington, DC., United States; and the University of Nebraska State Museum, Harold W. Manter Laboratory (HWML), Lincoln, Nebraska, United States. In addition, type and voucher specimens of some previously described species that were available from the USNM were examined for comparative purposes.

Ethical standards: Fishes were collected under Queensland General Fisheries Permits 187264 and 164063, and Moreton Bay Marine Park Permit QS2015/Man321. This study was conducted in compliance with all institutional, national and international guidelines on the care and use of animals.

## Results and discussion

Dactylogyrids currently known to occur in Moreton Bay, including those reported herein and those recorded in previously published studies, are listed in Table 1. Twenty-nine dactylogyrid species collected from marine fishes during the January 2016 survey are recorded below and include 12 new species, two new genera, and ten new host records and 13 new faunal records of previously described species for Moreton Bay. Descriptions of the new species, diagnoses of the new genera, and additional information concerning the remaining species follow.

Class Monogenoidea Bychowsky, 1937

Subclass Polyonchoinea Bychowsky, 1937

Order Dactylogyridea Bychowsky, 1937

Dactylogyridae Bychowsky, 1933

## *Atherinicus* Bychowsky & Nagibina, 1969

Type species: *Atherinicus cornutus* Bychowsky & Nagibina, 1969 from *Atherina forskali* (*sic*) Rüppell [now *Atherinomorus forskalii* (Rüppell)], Atheriniformes, Atherinidae [6], an apparent misidentification of the host (see Remarks). Also, from *Hepsetia pinguis* Nichols & Roemhild [now *Atherinomorus lacunosus* (Forster)], Atheriniformes, Atherinidae [63] and *Atherina bleekeri* Günther [now *Doboatherina bleekeri* (Günther)], Atheriniformes, Atherinidae [62]*.*

Other species: *Atherinicus bychowskyi* Zhukov, 1984 from *Atherina stipes* Müller & Troschel [now *Atherinomorus stipes* (Müller & Troschel)], Atheriniformes, Atherinidae [100]; *Atherinicus cubanus* Zhukov, 1984 from *Atherina stipes* Müller & Troschel [now *Atherinomorus stipes* (Müller & Troschel)], Atheriniformes, Atherinidae [100]; *Atherinicus hainanensis* Pan & Lu, 2005 from *Atherina bleekeri* Günther [now *Doboatherina bleekeri* (Günther)], Atheriniformes, Atherinidae [62]; *Atherinicus ophiocephalus* Zhukov, 1984 from *Atherina stipes* Müller & Troschel [now *Atherinomorus stipes* (Müller & Troschel)], Atheriniformes, Atherinidae [100]; *Atherinicus subserratus* Zhang, 2001 from *Atherina bleekeri* Günther [now *Doboatherina bleekeri* (Günther)], Atheriniformes, Atherinidae [97]; and *Atherinicus difficilis* n. sp. from *Atherinomorus vaigiensis* (Quoy & Gaimard), Atheriniformes, Atherinidae [*ex nobis*]*.*

### Emended diagnosis

Body fusiform, flattened dorsoventrally, comprising body proper (cephalic region, trunk, peduncle) and haptor. Tegument smooth. One or two terminal and two bilateral cephalic lobes; bilateral head organs present; bilateral groups of cephalic-gland cells posterolateral to pharynx. Eyespots present. Mouth subterminal, prepharyngeal; pharynx muscular; esophagus short; intestinal ceca two, confluent posterior to gonads, lacking diverticula. Genital pore midventral, near level of esophageal bifurcation. Gonads intercecal, completely overlapping (germarium ventral to testis). Testis entire; vas deferens dorsoventrally looping left intestinal cecum; seminal vesicle a simple dilation of distal vas deferens. One or two prostatic reservoirs. MCO a sclerotized tube arising from expanded proximal base; accessory piece present. Germarium entire; uterus delicate, lying along body midline. Ventral gonadal bar present or absent. Vaginal pore midventral; vagina lacking sclerotization. Seminal receptacle pregermarial. Vitellarium throughout trunk, except absent from regions of other reproductive organs. Haptor armed with dorsal and ventral anchor/bar complexes, seven pairs of similar hooks having normal dactylogyrid distribution. Dorsal and ventral anchors morphologically similar. Ventral and dorsal bars rod shaped. Each hook with comparatively large protruding, terminally blunt or flattened thumb and shank comprised of two subunits; proximal subunit of shank variable in length among hook pairs. Parasites of atheriniform fishes.

### Remarks

The emended diagnosis of *Atherinicus* was based on the published records of Bychowsky & Nagibina [6] and Zhang [97] and on available specimens of *Atherinicus difficilis* n. sp. The original descriptions of *A. hainanensis*, *A. bychowskyi*, *A. cubanus*, and *A. ophiocephalus* primarily involved the features of the haptoral hooks and anchor/bar complexes and were of minimal value in determining diagnostic features from internal anatomy [62, 100].

*Atherinicus* appears to be most similar to *Diversohamulus* Bychowsky & Nagibina, 1969 and *Hemirhamphiculus* Bychowsky & Nagibina, 1969 based on their species having overlapping gonads and haptoral hooks with flattened comparatively large protruding thumbs and shanks comprised of two subunits. *Atherinicus* differs from *Diversohamulus* by its species having a well-developed dorsal anchor/bar complex (dorsal bar absent, dorsal anchors reduced or vestigial in species of *Diversohamulus*). In addition, the copulatory complex of *Atherinicus* spp. is situated anteriorly in the trunk at level of the esophageal bifurcation, whereas in species of *Diversohamulus,* the copulatory complex lies ventrally near the midlength of the trunk. In species of *Hemirhamphiculus*, the proximal subunit of the hook shank is reduced, whereas in species of *Atherinicus*, it is well developed, often representing the majority of the total length of the hook.

The gonadal bar of *A. cornutus*, which at the time of the proposal of the genus was not known to occur in species of any other dactylogyrid genera, apparently influenced Bychowsky & Nagibina [6] to recognize *Atherinicus* as a distinct genus. One other dactylogyrid genus, *Gonocleithrum* Kritsky & Thatcher, 1983, contains species having a gonadal bar similar in shape and position within the body as that described for *A. cornutus*. *Atherinicus cornutus* differs from *Gonocleithrum* spp. by having a mid-ventral vaginal pore (sinistromarginal in species of *Gonocleithrum*), overlapping gonads (testis and germarium partially overlapping in *Gonocleithrum* spp.), and flattened protruding thumbs in the haptoral hooks (thumbs of hooks protruding and blunt, not flattened, in species of *Gonocleithrum*) [45]. Nonetheless, the presence of a gonadal bar in *A. cornutus* appears to have developed secondarily within the genus, and at this time, is not considered to be a diagnostic feature of the taxon.

Bychowsky & Nagibina [6] recorded the host of *A. cornutus* to be *Atherina forskali* (= *Atherinomorus forskalii*), clearly a misidentification of the host species. As many as seven other silversides and hardheads, at one time assigned to *Atherina*, are known to occur in the region of the South China Sea (type locality of *A*. *cornutus*), whereas *At. forskalii* as an endemic, is restricted to the Red Sea but now also occurring within the Mediterranean Sea as an invader *via* the Suez Canal [23, 83]. Although *A. cornutus* has subsequently been recorded from *At. pinguis* from the Red Sea [63] and from *Hypoatherina valenciennei* (Bleeker) [now *Doboatherina valenciennei* (Bleeker 1854)] off Hainan Island [62], additional collections of the atheriniform fishes occurring in these waters and the type locality will be required to determine the host range of this helminth species.

## *Atherinicus difficilis* n. sp.

urn:lsid:zoobank.org:act:2595CA1C-1DF9-405B-8819-1439D59E8642

Type host: Ogilby’s hardyhead, *Atherinomorus vaigiensis* (Quoy & Gaimard), Atheriniformes, Atherinidae.

Type locality: Moreton Bay off Dunwich, North Stradbroke Island, Queensland, Australia (27°29 ′S, 153°23 ′E), 17 January 2016.

Infection site: Gill lamellae.

Specimens studied: Holotype, QM G240828; 19 paratypes, QM G240829-240835, USNM 1692855-1692861, HWML 216999.

Etymology: The specific name is from Latin (*difficilis* = difficult) and refers to an uncertainty concerning the assignment of the species to *Atherinicus* because of the absence of a gonadal sclerite (present only in the type species of the genus).

### Description (Figs. 1–11)

Body robust. Cephalic region broad, with moderately developed cephalic lobes; single terminal cephalic lobe. Two bilateral pairs of head organs; bilateral groups of cephalic-gland cells posterolateral to pharynx; each group with 4–6 cells. One pair of eyespots; each eyespot with lens; eyespot granules small, ovate to subspherical; accessory chromatic granules comparatively large, ovate, scant throughout cephalic region. Mouth ventral, prepharyngeal on body midline. Pharynx ovate; esophagus short, bifurcating into intestinal ceca. Peduncle broad. Haptor hexagonal. Dorsal and ventral anchors similar in shape; each with elongate superficial root, short knob-like deep root, evenly curved shaft and elongate point. Ventral bar rod shaped, slightly bowed, with rounded ends directed laterally; dorsal bar generally straight or with slight bend at midlength, with truncated ends. Each hook with terminally flattened protruding thumb, delicate point and shaft; shank subunits uniform in width; proximal subunit variable in length among hook pairs; filamentous hook (FH) loop extending proximally from hooklet to level of union of shank subunits. Common genital pore near postpharyngeal margin or esophageal bifurcation. Gonadal sclerite absent. Testis elongate ovate; distal portion of vas deferens dilating to form fusiform seminal vesicle. Prostates not observed; prostatic reservoir lying to right of seminal vesicle. MCO a tubular shaft with perpendicular basal bend; accessory piece spatulate, with terminal hook. Germarium elongate, pyriform; oötype, oviduct, Mehlis’ gland not observed. Vaginal pore, vagina not observed; bilateral extracecal regions at level of seminal receptacle lacking vitelline folicles. Seminal receptacle submedial, immediately anterior to germarium. Vitellarium dense, coextensive with intestinal ceca and lateral to esophagus and posterior half of pharynx; bilateral vitelline ducts anterior to germarium, apparently overlying oviduct, oötype, Mehlis’ gland, base of uterus. Egg not observed.

**Figures 1–11:**
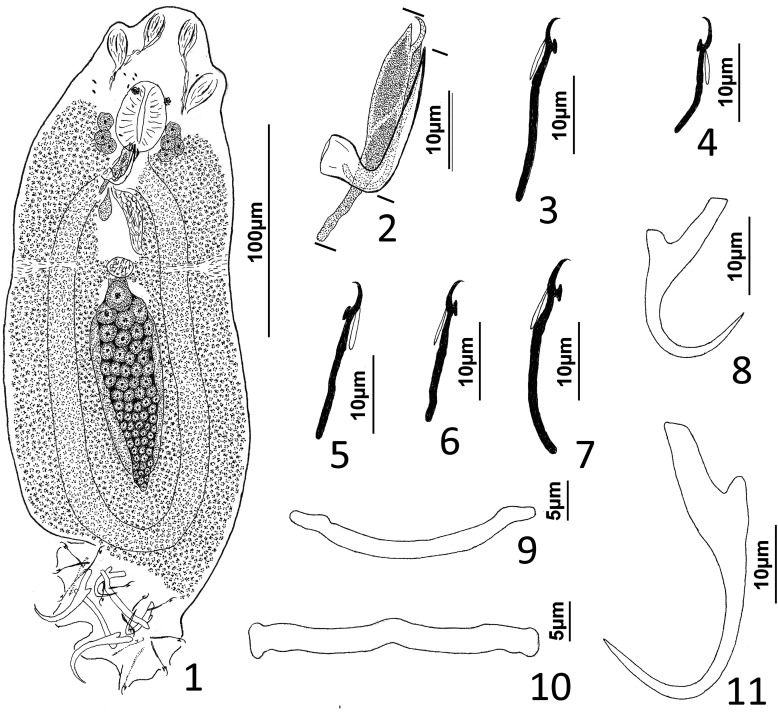
*Atherinicus difficilis* n. sp. from Ogilby’s hardyhead, *Atherinomorus vaigiensis*, Atherinidae. 1, Whole mount (ventral view, composite); 2, Copulatory complex (ventral view); 3, Hook (pair 7); 4, Hook (pair 5); 5, Hook (pair 2); 6, Hook (pair 1); 7, Hook (pair 6); 8, Ventral anchor; 9, Ventral bar; 10, Dorsal bar; 11, Dorsal anchor. Parallel lines on Fig. 2 indicate the limits of the dimensions measured.

Measurements: Body 251–368 (311; *n* = 11) long; greatest width (excluding haptor) 97–129 (113; *n* = 13). Haptor 77–100 (85; *n* = 8) wide. Ventral anchor 21–26 (23; *n* = 6) long; dorsal anchor 36–41 (38; *n* = 5) long. Ventral bar 29–34 (32; *n* = 6) long; dorsal bar 35–40 (38; *n* = 6) long. Hook pair 1, 19–22 (20; *n* = 5) long; pair 2, 23–27 (25; *n* = 5) long; pair 3, 20–26 (23; *n* = 4) long; pair 4, 20–25 (22; *n* = 4) long; pair 5, 16–18 (17; *n* = 3) long; pair 6, 23–28 (26; *n* = 6) long; pair 7, 23–27 (25; *n* = 4) long. Pharynx 25–34 (29; *n* = 12) long; 20–26 (23; *n* = 13) wide. Testis 44–70 (59; *n* = 4) long, 22–36 (30; *n* = 5) wide. Male copulatory organ 21–25 (22; *n* = 6) long; accessory piece 29–35 (32; *n* = 6) long. Germarium 63–111 (80; *n* = 10) long, 23–36 (28; *n* = 12) wide.

### Remarks

*Atherinicus difficilis* n. sp. is easily differentiated from *A. cornutus* by lacking a ventral gonadal sclerite (gonadal sclerite present in *A. cornutus* [6]). It differs from *A. cubanus*, *A. hainanensis, A. ophiocephalus*, and *A. subserratus*, by having the dorsal anchor noticeably larger than the ventral anchor (ventral anchors subequal or larger than the dorsal anchors in the latter species [62, 97, 100]). It is distinguished from *A. bychowskyi* by having shorter proximal subunits of the hook shanks compared to those of the respective homologs in *A. bychowskyi* [100]. In addition, the shaft of the MCO of *A. difficilis* has a nearly perpendicular basal bend and an distal acute tip, whereas in *A. cornutus* and *A. subserratus*, the shaft is U-shaped with an expanded tip; in *A. cubanus*, the shaft is delicate and forms a coil of approximately one ring; and in *A. hainanensis*, the shaft resembles that of *A. difficilis* n. sp., except that it has an expanded distal tip (acute tip in *A. difficilis*). The copulatory complexes of *A. bychowskyi* and *A. ophiocephalus* were not figured by Zhukov [100].

## *Chauhanellus duriensis* Lim, 1994

(Figs. 12–17)

**Figures 12–17:**
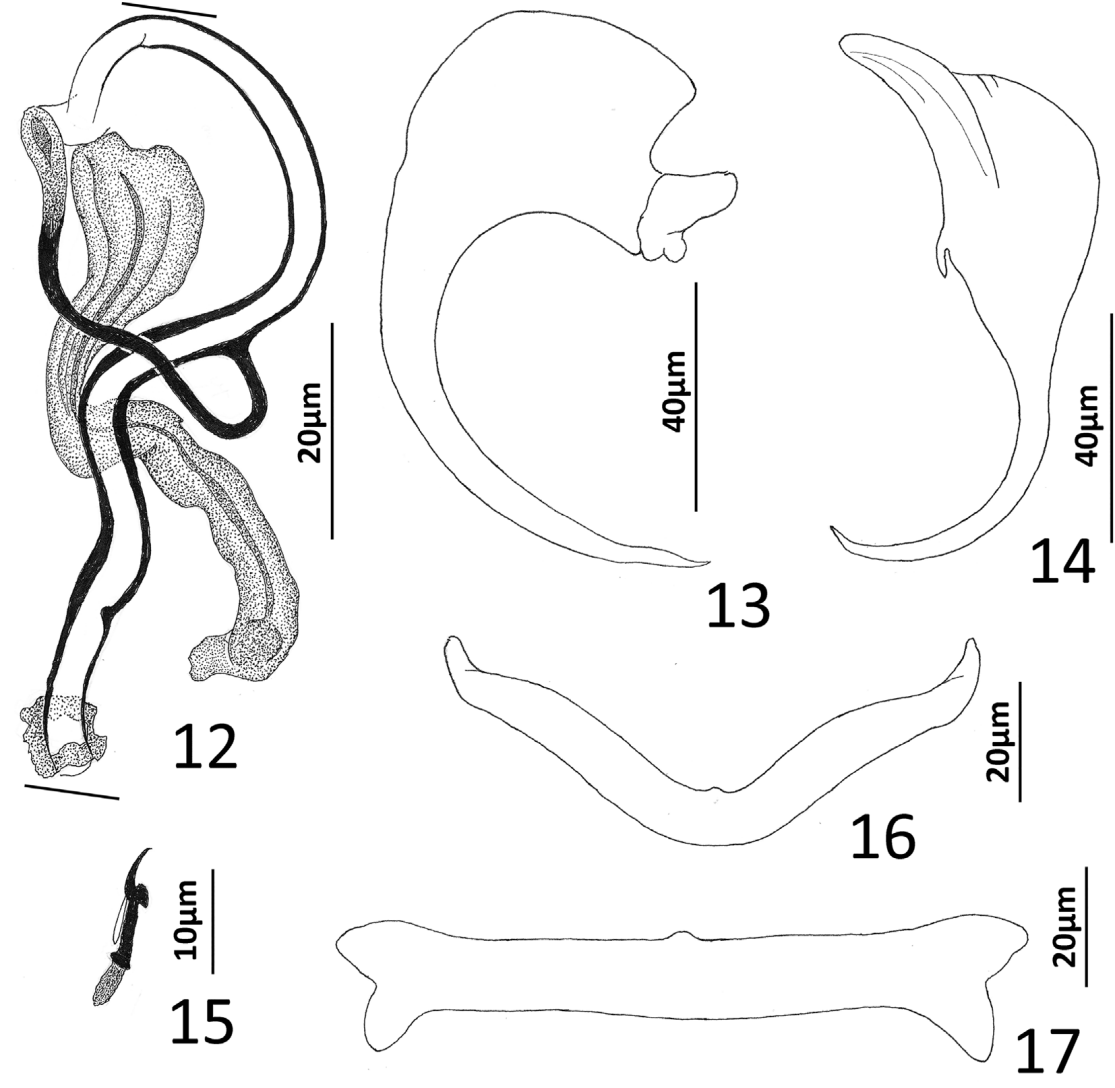
*Chauhanellus duriensis* Lim, 1994 from the Arafura catfish, *Pararius proximus*, Ariidae. 12, Copulatory complex (dorsolateral view); 13, Ventral anchor; 14, Dorsal anchor; 15, Hook; 16, Dorsal bar; 17, Ventral bar. Parallel lines on Fig. 12 indicate the limits of the dimension measured.

Type host: Giant catfish, *Arius thalassinus* (Rüppell) [now *Netuma thalassina* (Rüppell)], Siluriformes, Ariidae.

Type locality: Sungai Buloh, Selangor, Malaysia.

Current record: Arafura catfish, *Pararius proximus* (Ogilby), Siluriformes, Ariidae: Moreton Bay off Green Island, Queensland, Australia (27°25′ S, 153°14′ E), 16 January 2016.

Previous record: *N. thalassina* (as *A. thalassinus*): Sungai Buloh, Selangor, Malaysia [50].

Infection site: Gill lamellae.

Specimens studied: 9 voucher specimens, QM G240847–240850, USNM 1692871-1692874, HWML 217001.

Measurements (respective dimensions provided by Lim [50] follow in brackets those of present specimens): Body 831–989 (915; *n* = 4) [1,218–1,436 (1,294)] long; greatest width (excluding haptor) 138–165 (150; *n* = 4) [151–193 (176)]. Haptor 167–210 (188; *n* = 3) [227–319 (288)] wide. Ventral anchor 80–92 (87; *n* = 5) [78–80 (80)] long; dorsal anchor 92–102 (99; *n* = 5) [90–105 (95)] long. Ventral bar 115–123 (119; *n* = 4) [110–120 (117)] long; dorsal bar 88–96 (92; *n* = 5) [80–90 (84)] long. Hook 15–19 (17; *n* = 14) [12–16 (15)] long. Pharynx 54–71 (61; *n* = 4) wide. Testis 146–174 (161; *n* = 4) long, 63–79 (74; *n* = 4) wide. Copulatory complex 63–73 (69; *n* = 3) [70–84 (76)] long. Germarium 92–106 (98; *n* = 4) long, 44–65 (56; *n* = 4) wide.

### Remarks

The original description of *C. duriensis* by Lim [50] was adequate for identification of the species; the haptoral and copulatory sclerites of current specimens corresponded closely with those depicted in Lim’s figure 16. Lim [50] used specimens, fixed and mounted in ammonium-picrate glycerin (APG), for obtaining the measurements of the *Chauhanellus* spp. described in her paper. However, specimens fixed and mounted in APG are often placed under excessive coverslip pressure, resulting in considerable distortion and exaggeration of soft-body parts. Thus, comparison of the latter features was not useful in the differential analysis of the respective specimens.

The finding of *C. duriensis* on the gill lamellae of *Pararius proximus* in Moreton Bay is a new host record for the helminth and a new faunal record for the bay.

## *Diplectanotrema* sp. 1

(Figs. 18–21)

**Figures 18–21:**
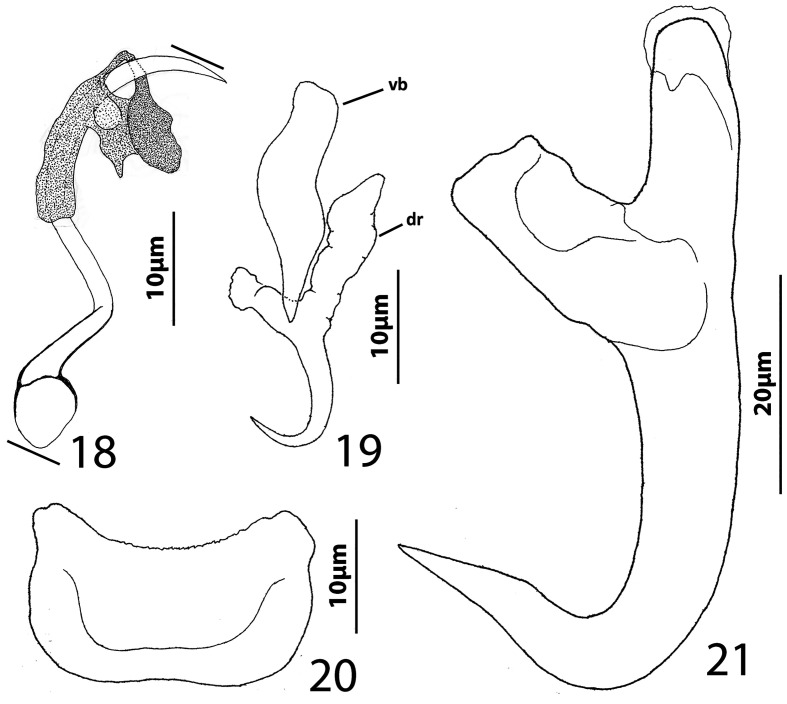
*Diplectanotrema* sp. 1 from the trumpeter whiting, *Sillago maculata*, Sillaginidae. 18, Copulatory complex (dorsal view); 19, Ventral anchor and ventral bar; 20, Dorsal bar; 21, Dorsal anchor. Abbreviations: dr, deep root of ventral anchor; vb, ventral bar. Parallel lines in Figure 18 indicate the limits of the dimension measured.

Probable syn. *Pseudempleurosoma* sp. of Hayward [19]

Host: Trumpeter whiting, *Sillago maculata* Quoy & Gaimard, Series Eupercaria, Sillaginidae.

Locality: Chain Banks Reef, Moreton Bay, Queensland, Australia (27°24′ S, 153°21′ E), 28 June 2016, 5 July 2016.

Infection site: Probably pharynx and/or esophagus (specimens found only in sediments).

Specimens studied: 4 voucher specimens, QM G241015–241018.

Measurements (For comparison, respective dimensions provided by Hayward [19] for *Pseudempleurosoma* sp. from *Sillago ingenuua* McKay follow in brackets those of present specimens): Body 689–779 (721; *n* = 3) [1,180] long; greatest width (excluding haptor) 224–275 (249; *n* = 3) [256]. Haptor 146–154 (150; *n* = 2) [113] wide. Ventral anchor 27–28 (*n* = 1) [24] long; dorsal anchor 62–70 (66; *n* = 2) [61] long. Ventral bar 26–27 (*n* = 1) [16] long; dorsal bar 26–27 (*n* = 1) [21] long. Hook 15–16 (*n* = 2) [13] long. Pharynx 62–66 (64; *n* = 2) [80] long, 72–80 (76; *n* = 2) [80] wide. Testis 46–63 (54; *n* = 2) long, 40–41 (*n* = 2) wide. Copulatory complex 39–40 (*n* = 1) [51] long. Germarium 71–77 (74; *n* = 2) long, 71–74 (73; *n* = 2) wide.

## *Diplectanotrema* sp. 2

Host: Crested morwong, *Goniistius vestitus* (Castelnau), Centrarchiformes, Latridae.

Locality: Moreton Bay off Amity Point, North Stradbroke Island, Queensland, Australia (27°23′ S, 153°26′ E), 16 November 2015.

Infection site: Probably pharynx and/or esophagus (specimens found in sediments).

Specimen studied: Voucher specimen, QM G241019.

Measurements: Body 903–904 long; greatest width (excluding haptor) 179–180. Haptor 93–94 wide. Dorsal anchor 53–54 long. Pharynx 74–75 long, 70–71 wide. Testis 40–41 long, 34–35 wide. Copulatory complex 40–41 long. Germarium 83–84 long, 68–69 wide.

### Remarks

Five dactylogyrid genera, *Diplectanotrema* Johnston & Tiegs, 1922, *Pseudempleurosoma* Yamaguti, 1965, *Neodiplectanotrema* Gerasev, Gaevskaja & Kovaleva, 1987, *Paradiplectanotrema* Gerasev, Gaevskaja, & Kovaleva, 1987, and *Pseudodiplectanotrema* Gerasev, Gaevskaja, & Kovaleva, 1987 comprise a subordinate, apparently monophyletic but unnamed taxon within the Dactylogyridae [16]. A sixth genus of this “*Diplectanotrema* group,” *Metadiplectanotrema* Gerasev, Gaevskaja, & Kovaleva, 1987, was placed in subjective synonymy with *Pseudempleurosoma* [75]. Species of the “*Diplectanotrema* group” generally parasitize the pharynx and esophagus of their fish hosts, which currently represent 16 orders, two so-called series (the Eupercaria and Carangaria), and about 35 families of marine teleosts. These helminths are highly redundant morphologically and have often been differentiated and described based on their respective hosts, misinterpretation of structure, and/or on small morphological differences that likely represent intraspecific variation and/or secondarily evolved characters. As a result, the group probably contains synonyms at both the specific and generic levels, with some species, at least, having wide host ranges.

The assignments of current specimens of the “*Diplectanotrema* group” from Moreton Bay to *Diplectanotrema* are both provisional. Only four specimens of *Diplectanotrema* sp. 1 and one of *Diplectanotrema* sp. 2 were found in their respective hosts; all specimens were insufficient for identification, description, or generic assignment. Based on their occurrence on a species of *Sillago*, the four specimens of *Diplectanotrema* sp. 1 may be conspecific with *Pseudempleurosoma* sp. of Hayward [19], which was reported from the bay sillago, *Sillago ingenuua* McKay, in Malaysia [19]. Conspecificity requires confirmation.

The occurrences of both *Diplectanotrema* spp. 1 and 2 in the fishes of Moreton Bay represent new fauna records for the bay, and the presence of *Diplectanotrema* sp. 1 in *Sillago maculata* and *Diplectanotrema* sp. 2 in *Goniistius vestitus* represents new host records for the genus group. The finding of *Diplectanotrema* sp. 2 infecting *G. vestitus* represents the first occurrence of a member of the group infecting a latrid host.

## *Diversohamulus similis* n. sp.

urn:lsid:zoobank.org:act:38D03BD2-DFAD-4B92-B3C4-267ADF7E10DB

Type host: Ogilby’s hardyhead, *Atherinomorus vaigiensis* (Quoy & Gaimard), Atheriniformes, Atherinidae.

Type locality: Moreton Bay off Dunwich, North Stradbroke Island, Queensland, Australia (27°29′ S, 153°23′ E), 17 January 2016.

Infection site: Gill lamellae.

Specimens studied: Holotype, QM G240836; 13 paratypes, QM G240837–240841, USNM 1692862-1692867, HWML 217000; 2 vouchers (immature specimens as *Diversohamulus* sp.), QM G240842–240843.

Etymology: The specific name (an adjective) is from Latin (*similis* = somewhat like, resembling) and refers to the morphological similarity of the species to *Diversohamulus tricuspidatus* Bychowsky & Nagibina, 1969.

### Description (Figs. 22–30)

Body slender, elongate, flattened dorsoventrally, with nearly parallel lateral margins. Tegument smooth. Cephalic region broad, with poorly developed cephalic lobes. Three bilateral pairs of head organs; pair of ducts located between two anterior-most head organs draining small medial prepharyngeal glands. Bilateral groups of large cephalic-gland cells posterolateral to pharynx; each group with 4–6 cells. Two pairs of eyespots; lenses in members of both pairs; eyespot granules small, ovate to subspherical; accessory chromatic granules comparatively large, ovate, scattered throughout cephalic region from just posterior to pharynx anteriorly to head organs. Mouth ventral, prepharyngeal on body midline. Pharynx ovate; esophagus long, extending posteriorly from pharynx to about 1/3 body length before bifurcating to two intestinal ceca; intestinal ceca lacking diverticula, united in trunk posterior to gonads. Peduncle broad, undifferentiated from trunk, slightly tapered posteriorly. Haptor armed with ventral anchor/bar complex, pair of dorsal anchors, 14 (7 pairs) of hooks with normal dactylogyrid distribution. Ventral anchor with curved shaft and point, and enlarged base having elongate superficial root and short knob-like deep root. Dorsal anchor reduced, with perpendicular superficial root, short knob-like deep root, evenly curved shaft and point; each dorsal anchor associated with non-descript sclerite. Ventral bar rod shaped, with rounded ends; dorsal bar absent. Each hook with enlarged, protruding, terminally depressed and flattened thumb, delicate point and shaft, uniform shank composed of two poorly differentiated subunits; proximal subunit variable in length among hook pairs; FH loop extending proximally from hooklet to level of union of shank subunits. Gonads overlapping; testis dorsal to germarium. Genital pore not observed. Testis ovate; vas deferens apparently looping left intestinal cecum, distal portion of vas deferens apparently dilating to form fusiform seminal vesicle. Prostates not observed; prostatic reservoir lying to right of copulatory complex. Copulatory complex and small reservoir containing granular material located in external pouch protruding from sinistroventral body surface near midlength of trunk. MCO an arcing tubular shaft arising from slightly expanded base; tip of shaft recurved. Accessory piece proximally spatulate, with heavy lateral margins, articulated to base of MCO (Fig. 23). Germarium pyriform; oötype, oviduct, Mehlis’ gland not observed; uterus delicate, usually empty or sometimes containing single egg. Vaginal pore not observed, apparently associated with plate-like vaginal/gonadal sclerite having delicate spine at each end; vaginal canal not observed. Seminal receptacle submedial, immediately internal to vaginal sclerite. Vitellarium dense, coextensive with intestinal ceca and lateral to esophagus; bilateral vitelline ducts anterior to germarium, apparently overlying oviduct, oötype, Mehlis’ gland, base of uterus. Egg subspherical, lacking filaments.

**Figures 22–31:**
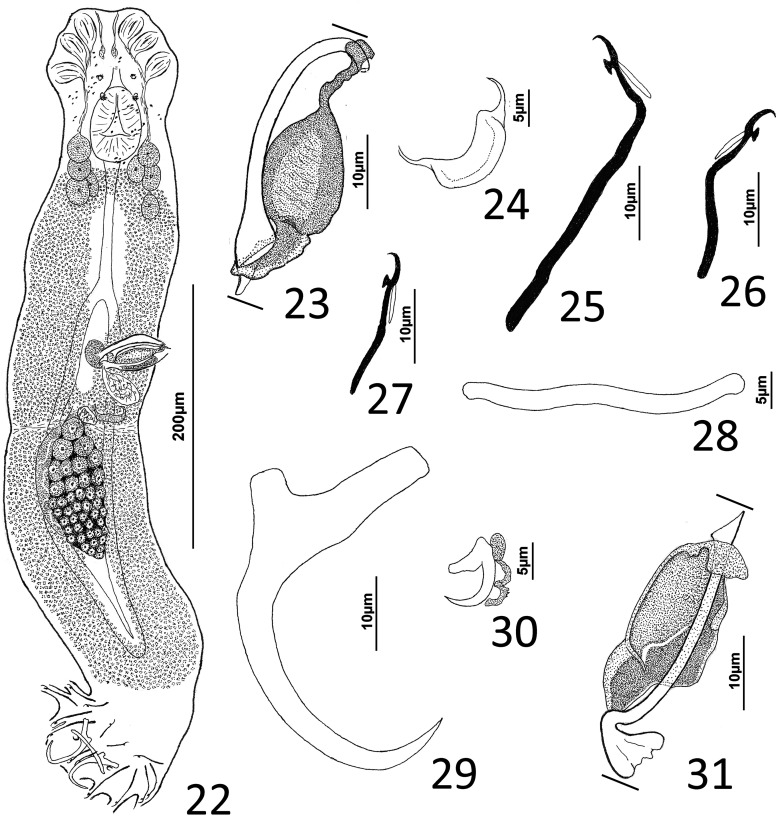
Species of *Diversohamulus* infecting Ogilby’s hardyhead, *Atherinomorus vaigiensis*, Atherinidae. Figures 22–30 *Diversohamulus similis* n. sp. 22, Whole mount (ventral view, composite); 23, Copulatory complex (ventral view); 24, Gonadal sclerite; 25, Hook (pair 6); 26, Hook (pair 3); 27, Hook (pair 1); 28, Ventral bar; 29, Ventral anchor; 30, Dorsal anchor. Figure 31 Copulatory complex of *Diversohamulus tricuspidatus* Bychowsky & Nagibina, 1969 (ventral view). Parallel lines on Figures 23 and 31 indicate the limits of the dimensions measured.

Measurements: Body 507–738 (613; *n* = 4) long; greatest width (excluding haptor) 80–116 (96; *n* = 5). Haptor 96–127 (116; *n* = 5) wide. Ventral anchor 40–46 (43; *n* = 7) long; dorsal anchor 9–12 (10; *n* = 6) long. Ventral bar 39–45 (42; *n* = 5) long. Hook pair 1, 18–23 (21; *n* = 4) long; pair 2, 25–31 (27; *n* = 5) long; pair 3, 24–29 (26; *n* = 6) long; pair 4, 25–33 (28; *n* = 6) long; pair 5, 16–20 (18; *n* = 5) long; pair 6, 38–51 (46; *n* = 7) long; pair 7, 29–34 (32; *n* = 6) long. Pharynx 53–66 (61; *n* = 6) long; 34–51 (43; *n* = 6) wide. Testis 53–63 (58; *n* = 3) long, 40–45 (43; *n* = 3) wide. Copulatory complex 36–40 (38; *n* = 7) long. Germarium 69–95 (78; *n* = 4) long, 23–36 (32; *n* = 4) wide.

### Remarks

*Diversohamulus* was proposed for *Diversohamulus tricuspidatus* Bychowsky & Nagibina, 1969 occurring on the gills of the Red Sea hardyhead silverside, *Atherina forskali* Rüppell (*sic*) [now *Atherinomorus forskalii* (Rüppel)], from the South China Sea off Hainan, China [6]. The genus was primarily characterized by having reduced (vestigial?) dorsal anchors and by lacking a dorsal bar. Specimens identified as *D. tricuspidatus* were reported from the Samoan silverside, *Atherina afra* Peters (now *Hypoatherina temminckii* (Bleeker)], and the wide-banded hardyhead silverside, *Hepsetia pinguis* Nickols & Roemhild [a synonym of *Atherinomorus lacunosus* (Forster)], from Gulf of Aqaba off Eilat, Israel, and the Gulf of Suez [63]. However, Paperna’s [63] figures 10, 11, 13, & 14 of the copulatory complex and dorsal anchors suggest that his specimens likely represented an undescribed species of the genus. Finally, Zhang’s [97] report of *D. tricuspidatus* from the gills of the Sumatran silverside, *Atherina bleekeri* Günther [now *Doboatherina bleekeri* (Günther)], from Huiyang, Guangdong Province, China, is apparently the last published record of *D. tricuspidatus*. Only one other species, *Diversohamulus curvionchus* Zhukov, 1984 from the hardhead silverside, *Atherina stipes* Müller & Troschel [now *Atherinomorus stipes* (Müller & Troschel)], off Havana, Cuba, was subsequently described [100].

*Diversohamulus similis* n. sp. is most similar to *D. tricuspidatus* based on the comparative morphology of the haptoral sclerites. It differs from *D. tricuspidatus* by lacking a proximal twist and an acute distal tip of the shaft of the MCO (compare Figs. 23, 31). It differs from *D. curvionchus* by having a comparatively short shaft and point of the dorsal anchor, ventral anchors with comparatively short and robust shafts and points, and an arcing shaft of the MCO (shaft of MCO with a loose coil of one or two rings in *D. curvionchus*; see figure 2б in Zhukov [100]).

## *Diversohamulus tricuspidatus* Bychowsky & Nagibina, 1969

(Fig. 31)

*Type host*: Red Sea hardyhead silverside, *Atherina forskali* (*sic*) Rüppell, an apparent misidentification and misspelling of the host [now *Atherinomorus forskalii* (Rüppell)] (see Remarks for *Atherinicus*), Atheriniformes, Atherinidae.

*Type locality*: South China Sea off Hainan, China.

*Current record*: *Atherinomorus vaigiensis*: Moreton Bay off Dunwich, North Stradbroke Island, Queensland, Australia (27°29′ S, 153°23′ E), 17 January 2016.

*Previous records*: *Atherinomorus* sp. (as *Atherina forskali*): South China Sea off Hainan, China [6]. *Hypoatherina temminckii* (as *Atherina afra* Peters): Red Sea, NW of Gulf of Eilat (Eilat port area); and Suez Gulf, El Blaim lagoon [63]. *Atherinomorus lacunosus* (as *Hepsetia pinguis*): Red Sea, NW of Gulf of Eilat [63]. *Doboatherina bleekeri* (Günther) (as *Atherina bleekeri*): Huiyang, Guangdong Province, China [97].

*Infection site*: Gill lamellae.

*Specimens studied*: 6 voucher specimens, QM G240844–240846, USNM 1692869-1692870.

Measurements (Respective dimensions provided by Bychowsky & Nagibina [6] follow in brackets those of present specimens): Body 527–528 (*n* = 1) [510–690] long; greatest width (excluding haptor) 98–99 (*n* = 1) [150–170]. Haptor 109–110 (*n* = 1) wide. Ventral anchor 42–48 (45; *n* = 4) long [34–39]; dorsal anchor 10–12 (11; *n* = 2) [6–8] long. Ventral bar 42–47 (45; *n* = 4) long. Hook pair 1, 18–22 (20; *n* = 2) long; pair 2, 25–29 (27; *n* = 3) long; pair 3, (23–27 (25; *n* = 3) long; pair 4, 27–29 (28; *n* = 2) long; pair 5, 18–21 (19; *n* = 3) long; pair 6, 42–51 (47; *n* = 4) long; pair 7, 28–33 (31; *n* = 3) long [hooks 15–38 long]. Pharynx 62–63 (*n* = 1) long; 46–47 (*n* = 1) wide [50–70 in diameter]. Copulatory complex 36–41 (38; *n* = 5) long.

### Remarks

Only six specimens of *D. tricuspidatus* were collected during the present study; all were insufficient to develop a redescription of the species. Nonetheless, the specimens were easily identified as *D. tricuspidatus* based on the morphology of the copulatory complex (Fig. 31), the shaft of the MCO of which possessed a small proximal loop or bend and an acute distal tip with a diagonal opening. The finding of *D. tricuspidatus* on *A. vaigiensis* in Moreton Bay represented a new host record for the helminth and a new fauna record for the bay.

## *Ecnomotrema* n. gen.

urn:lsid:zoobank.org:act:F31924F4-31F6-4692-964B-8FCF79C20AB7

Type species: *Ecnomotrema cetiosum* n. sp. from Ogilby’s hardyhead, *Atherinomorus vaigiensis* (Quoy & Gaimard), Atheriniformes, Atherinidae. The genus is monotypic.

Etymology: The generic name is from Greek (*eknomos* = marvelous, wondrous) appended to the commonly used ending (-trema) for parasitic platyhelminths.

### Diagnosis

Body broad, dorsoventrally flattened, comprising body proper (cephalic region, trunk, peduncle) and haptor. Tegument smooth. One or two terminal and two bilateral cephalic lobes; bilateral head organs present; bilateral groups of cephalic-gland cells posterolateral to pharynx. Eyespots present. Mouth subterminal, prepharyngeal; pharynx muscular; esophagus short; intestinal ceca two, confluent posterior to gonads, with thick walls, lacking diverticula. Genital pore midventral. Gonads intercecal, tandem (testis postgermarial). Testis entire or lobed; vas deferens apparently looping left intestinal cecum dorsoventrally; seminal vesicle a simple dilation of distal vas deferens. Prostatic reservoir present. Copulatory complex comprising articulated MCO and accessory piece. MCO a sclerotized tube arising from expanded proximal base; accessory piece articulated to base of MCO. Germarium entire; uterus delicate, lying along body midline. Vaginal pore midventral; vagina with distal sclerotization. Seminal receptacle pregermarial. Vitellarium throughout trunk, except absent from regions of other reproductive organs. Haptor plate-like, with posterior flap directed ventrally from posterior haptoral margin, armed with ventral anchor/bar complex; five pairs of similar hooks observed, representing hook pairs 2, 3, 4, 6, and 7 normally distributed in haptor. Hook morphology undetermined. Parasites of atheriniform fishes.

### Remarks

*Ecnomotrema* is distinguished from most marine dactylogyrid genera by its species having a mid-ventral vaginal pore, a distally sclerotized vaginal canal, a lobed testis, a posterior flap overlying the posterior margin of the haptor, and by lacking a dorsal anchor/bar complex. Most marine dactylogyrid genera have species with a dextromarginal vaginal pore, a vaginal canal that may or may not have sclerotized components, an entire testis lacking lobes, and a simple lobulate haptor having both dorsal and ventral anchor/bar complexes. The genus is probably most similar to *Hareocephalus* Young, 1969, which includes species with similar body shapes, dextroventral vaginal pores, and lobate testes. *Ecnomotrema* is easily differentiated from *Hareocephalus* by its species having only a ventral anchor/bar complex in the haptor (both dorsal and ventral complexes present in species of *Hareocephalus*) and in the morphology of the haptor (a non-muscular prehaptoral saucer-shaped flap from which the haptor descends in *Hareocephalus* species, see [31]).

## *Ecnomotrema cetiosum* n. sp.

urn:lsid:zoobank.org:act:64A8D817-C622-46CA-96E4-28F6D3F0DDC8

Type host: Ogilby’s hardyhead, *Atherinomorus vaigiensis* (Quoy & Gaimard), Atheriniformes, Atherinidae.

Type locality: Moreton Bay off Dunwich, North Stradbroke Island, Queensland, Australia (27°29′ S, 153°23′ E), 17 January 2016.

Infection site: Gill lamellae.

Specimens studied: Holotype, QM G241020; paratype, USNM 1693012.

Etymology: The specific name (an adjective) is from Greek (*ketios* = large) and refers to the species’ size compared to that of many other dactylogyrid species.

### Description (Figs. 32–35)

Cephalic region broad, with poorly developed cephalic lobes; terminal cephalic lobe single. Three bilateral pairs of head organs; bilateral groups of cephalic-gland cells posterolateral to pharynx; each group with 4–6 cells. Two pairs of eyespots; each eyespot with lens; eyespot granules small, ovate to subspherical; accessory chromatic granules comparatively large, ovate, scattered throughout cephalic region. Mouth midventral, prepharyngeal within small ventral concavity. Pharynx ovate; esophagus short. Peduncle broad, slightly tapered posteriorly. Haptor rounded, plate-like; posterior flap with thick anterior margin, extending anteriorly to level of posterior margin of ventral bar. Ventral anchor with well-developed basal roots, elongate base, evenly curved short shaft, short point. Ventral bar rod shaped, slightly bowed, with anteromedial narrowing and rounded ends. Common genital pore on body midline near midlength of trunk. Testis hemispherical, with about six lobes; seminal vesicle lying ventral to left intestinal cecum at level of MCO. Prostates not observed; prostatic reservoir small, lying to left of genital pore. MCO a straight tubular shaft arising from bulbous base; accessory piece branched near midlength, branches converging distally. Germarium tear-drop shaped; oötype, oviduct, Mehlis’ gland not observed. Vaginal pore simple; distal vaginal sclerotization funnel shaped. Seminal receptacle submedial, small, spherical. Vitellarium comprised of sparse follicles coextensive with intestinal ceca, with laterally branched digitiform processes; bilateral vitelline ducts anterior to germarium. Egg not observed.

**Figure 32:**
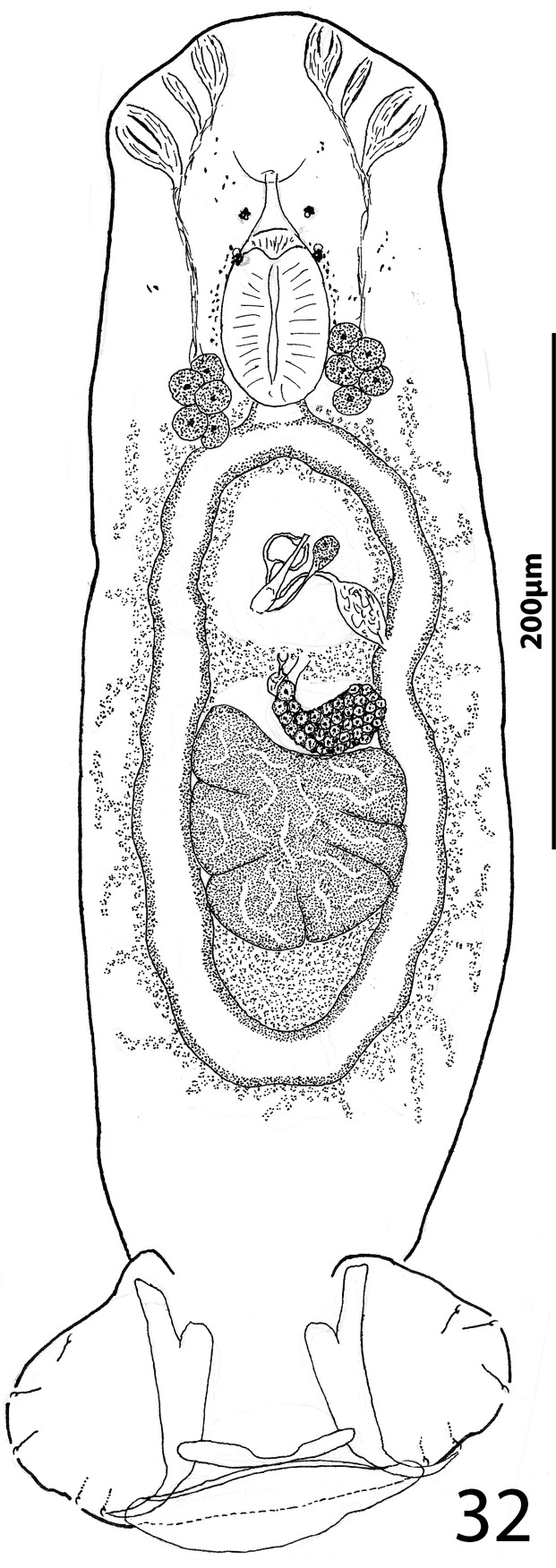
Whole mount (ventral view, composite) of *Ecnomotrema cetiosum* n. sp. from Ogilby’s hardyhead, *Atherinomorus vaigiensis*, Atherinidae.

**Figures 33–35:**
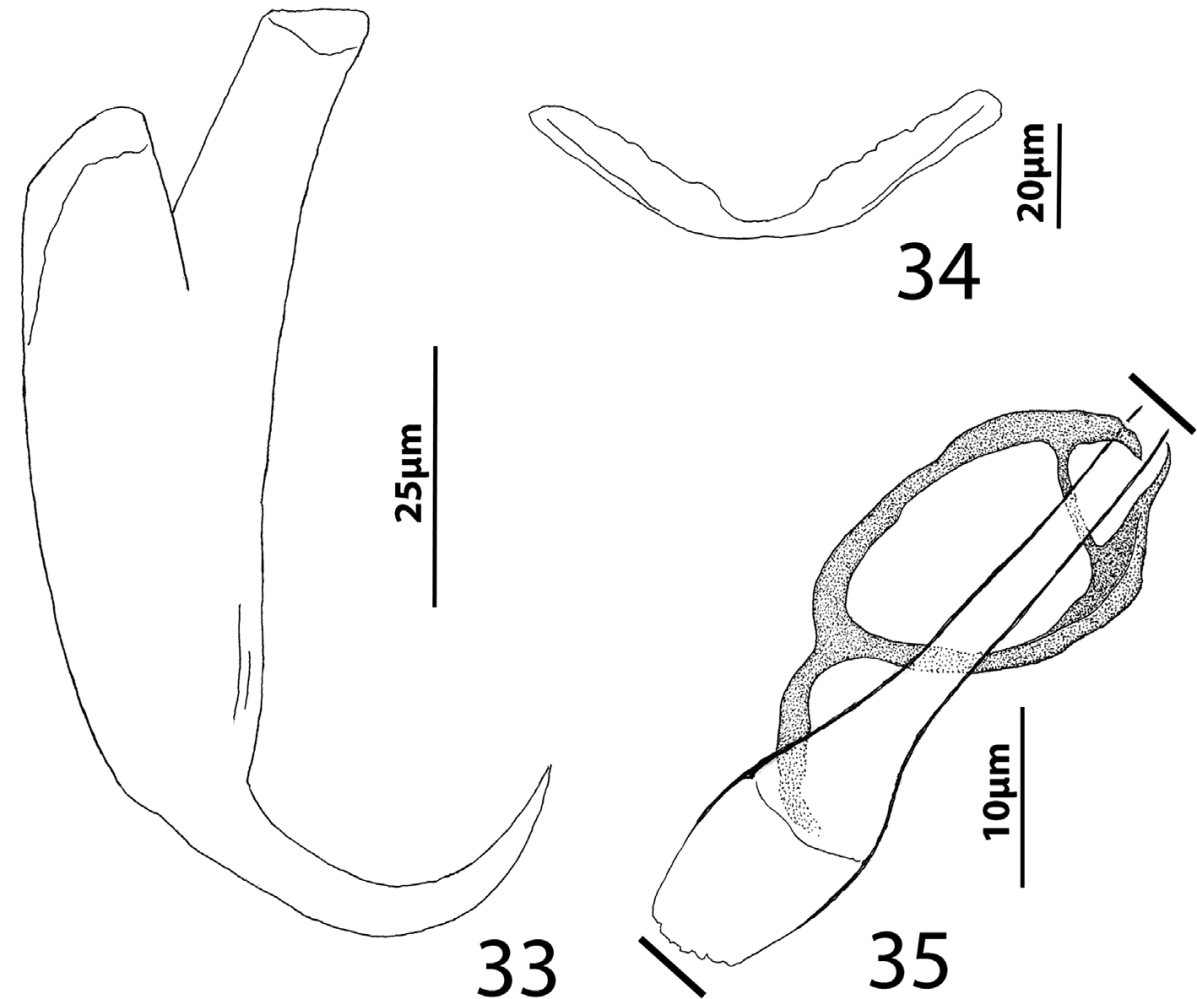
Haptoral and copulatory sclerites of *Ecnomotrema cetiosum* n. sp. 33, Ventral anchor; 34, Ventral bar; 35, Copulatory complex (ventral view). Parallel lines on Figure 35 indicate the limits of the dimension measured.

Measurements: Body 590–605 (598; *n* = 2) long; greatest width (excluding haptor) 168–184 (176; *n* = 2). Haptor 195–216 (206; *n* = 2) wide. Ventral anchor 91–96 (94; *n* = 2) long. Ventral bar 61–78 (69; *n* = 2) long. Hook 13–15 (14; *n* = 6) long. Pharynx 67–68 (*n* = 2) long; 43–45 (44; *n* = 2) wide. Testis 85–101 (93; *n* = 2) long, 82–94 (88; *n* = 2) wide. Copulatory complex 42–49 (45; *n* = 2) long. Germarium 43–64 (53; *n* = 2) long, 21–25 (23; *n* = 2) wide.

### Remarks

*Ecnomotrema cetiosum* n. sp. is the type species of the genus.

## *Euryhaliotrema spariense* (Roubal, 1981) n. comb.

Syns *Haliotrema spariensis* Roubal, 1981; *Haliotrema spariense* Roubal, 1981

(Figs. 36–42)

**Figures 36–42:**
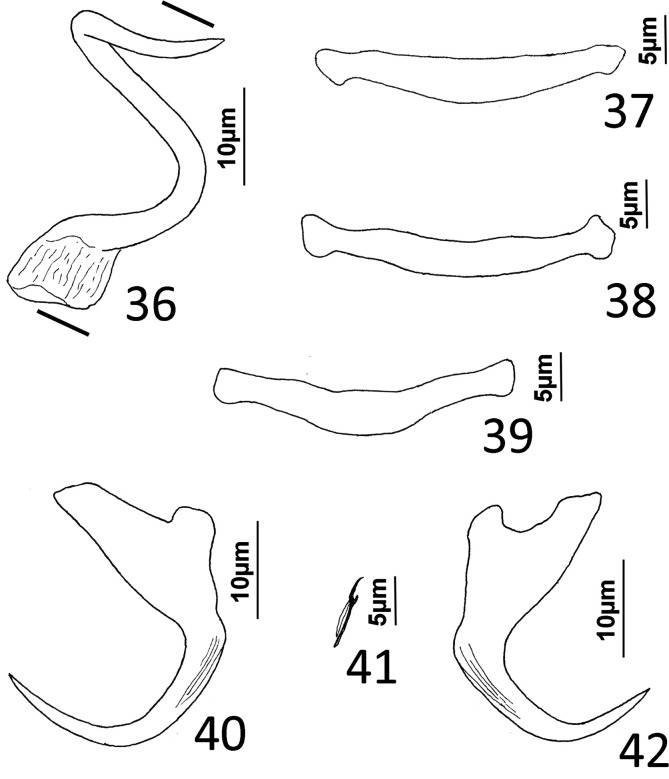
*Euryhaliotrema spariense* (Roubal, 1981) n. comb. from the yellowfin bream, *Acanthopagrus australis*, Sparidae. 36, Male copulatory organ (ventral view); 37, 38, Dorsal bars; 39, Ventral bar; 40, Ventral anchor; 41, Hook; 42, Dorsal anchor. Parallel lines on Figure 36 indicate the limits of the dimension measured.

Type host: Yellowfin bream, *Acanthopagrus australis* (Günther), Series Eupercaria, Sparidae.

Type locality: Coffs Harbour region, New South Wales, Australia.

Current record: *A. australis*: Moreton Bay off Peel Island, Queensland, Australia (27°30′ S, 153°20′ E), 14 January 2016.

Previous records (all as *H. spariensis*): *A. australis*: Coffs Harbour region, New South Wales, Australia [67]; unnamed estuary at Red Rock, New South Wales, Australia (29°59′ S, 153°13′ E) and Clarence River, 100 km N of Red Rock (coordinates not available) [68–71]; Brisbane, Townsville, & Gladstone, Queensland, Australia [8]; Newcastle and Coffs Harbor, New South Wales, Australia [8]; coastal waters off eastern Australia (Bandsian, Peronian, and Maugean Marine Biogeographical Regions of Australia) [9]; Moreton Bay, Queensland, Australia (27°20′ S, 153°10′ E) [8, 71–74]; Gold Coast, Queensland, Australia (29°58′ S, 153°25′ E) [73]. *Acanthopagrus berda* (Forsskål): Broome, Western Australia [8]; northern coastal waters off Australia (Dampierian, Banksian Regions) [9]. *Acanthopagrus butcheri* (Munro): Perth and Stokes Inlet, Western Australia [8]; Coorong, Southern Australia [8]; Melbourne and Lakes Entrance, Victoria, Australia [8]; Swansea and Eden, New South Wales, Australia [8]; southern coastal waters off Australia (Flindersian and Maugean Regions off Australia) [9]. *Acanthopagrus latus* (Houttuyn): Point Sampson and Stokes Inlet, Western Australia [8]; northwestern coastal waters off Australia (Dampierian Region) [9].

Infection site: Gill lamellae.

Specimens studied: 5 voucher specimens, QM G240851–240853, USNM 1692875-1692876.

Measurements (respective measurements from the original description follow in brackets those of current specimens): Body 515–516 (*n* = 1) [433–730 (573)] long; greatest width (excluding haptor) 76–77 (*n* = 1) [97–151 (103)]. Haptor 71–72 (*n* = 1) [56–97 (78)] wide. Ventral anchor 24–28 (26; *n* = 4) long; dorsal anchor 24–28 (25; *n* = 4) long [comparable measurements for anchors not available in Roubal (1981)]. Ventral bar 21–31 (28; *n* = 4) [24–33 (28)] long; dorsal bar 28–34 (31; *n* = 4) [30–59 (37)] long. Hook 10–12 (11; *n* = 4) long. Pharynx 35–36 (*n* = 1) [24–38 (34)] wide. Male copulatory organ 29–39 (33; *n* = 4) [17–33 (22)] long.

### Remarks

Roubal [67] described this species as *Haliotrema spariensis* from the gills of yellowfin bream from New South Wales, Australia. The helminth is a common parasite of Australian bream, having been reported from four species of *Acanthopagrus* occurring in all coastal regions of Australia [8, 9]. During the present survey, however, the intensity of the parasite on the gills of *A. australis* was low, with only five specimens collected from two of nine yellowfin bream examined (prevalence = 22%). Although the five specimens, four mounted unstained in Gray & Wess medium and one poorly stained and mounted in Canada balsam, were insufficient for a redescription of the species, they differed from the original description by Roubal [67] by possessing dorsal and ventral anchors having pronounced deep humps at the unions of the shafts and anchor bases, and by having hooks with well-defined upright acute thumbs; Byrnes [8], however, depicted the dorsal and ventral anchor humps in his figures 15, 16. These features along with others associated with the copulatory complex (MCO a loose coil and accessory piece absent) and the internal anatomy indicated that the species belonged in *Euryhaliotrema* Kritsky & Boeger, 2002, to which it is transferred as *Euryhaliotrema spariense* (Roubal, 1981) n. comb.

*Euryhaliotrema spariense* belongs to a group of congeners that lack an accessory piece in the copulatory complex and possess an MCO with a delicate coiled or meandering tube arising from an expanded base. In addition to *E. spariense*, the group includes *Euryhaliotrema adelpha* Kritsky & Justine, 2012, *Euryhaliotrema nanaoense* (Li, Yan, Lan, & Huang, 2005) Kritsky, 2012, *Euryhaliotrema paululum* Kritsky & Justine, 2012, *Euryhaliotrema spirotubiforum* (Zhang, Yang, & Liu, 2001) Wu, Zhu, Xie, & Liu, 2006, and *Euryhaliotrema youngi* Kritsky, 2012 all from lutjanid fishes; *Euryhaliotrema cribbi* (Plaisance & Kritsky) Kritsky, 2012 from chaetodontid fishes; *Euryhaliotrema kurodai* (Ogawa & Egusa, 1978) Kritsky, 2012 from sparid fishes; and *Euryhaliotrema solenophallus* Kritsky, 2019 from a monodactylid (see [30, 33]). *Euryhaliotrema spariense* is most similar to *E. kurodai*, from which it differs by having a counterclockwise coiled shaft of the MCO (shaft simple, arched in *E. kurodai*) (compare Fig. 36 with figures 2, 3 in Ogawa & Egusa [61]).

## *Glyphidohaptor phractophallus* Kritsky, Galli, & Yang, 2007

Type host: Mottled spinefoot, *Siganus fuscescens* (Houttuyn), Acanthuriformes, Siganidae.

Type locality: Great Barrier Reef off Heron Island, Queensland, Australia (23°27′ S, 151°55′ E), 16 July 2001.

Current records: *S. fuscescens*: Moreton Bay off Green Island, Queensland, Australia (27°25′ S, 153°14′ E), 11, 12 January 2016; Moreton Bay off Peel Island, Queensland, Australia (27°30′ S, 153°20′ E), 14 January 2016.

Previous records: The only previous record for *G. phractophallus* is that reported in the original description of the species [39].

Infection site: Gill lamellae.

Specimens studied: 13 voucher specimens (from Green Island), QM G240955–240959, USNM 1692962-1692965, HWML 217020; voucher specimen (from Peel Island), QM G240954.

Measurements (respective measurements from the original description follow in brackets those of current specimens): Body 634–789 (742; *n* = 8) [608–876 (731; *n* = 26)] long; greatest width (excluding haptor) 116–244 (183; *n* = 8) [141–154 (186; *n* = 27)]. Haptor 80–104 (94; *n* = 7) [90–112 (99; *n* = 20)] wide. Ventral anchor 47–55 (49; *n* = 5) [47–57 (52; *n* = 10)] long; dorsal anchor 47–62 (52; *n* = 4) [50–56 (53; *n* = 9)] long. Ventral bar 30–36 (32; *n* = 4) [30–39 (36; *n* = 9)] long; dorsal bar 30–36 (33; *n* = 3) [33–40 (36; *n* = 5)] long. Hook 12–14 (13; *n* = 9) [11–13 (12; *n* = 9)] long. Pharynx 47–53 (51; *n* = 5) long, 35–42 (39; *n* = 5) [29–45 (39; *n* = 27)] wide. Testis 113–148 (130; *n* = 5) [119–193 (155; *n* = 24)] long, 47–83 (61; *n* = 5) [37–70 (53; *n* = 24)] wide. Copulatory complex 51–59 (53; *n* = 4) [44–60 (52; *n* = 12)] long. Germarium 55–65 (61; *n* = 5) long, 34–48 (43; *n* = 5) [26–39 (31; *n* = 25)] wide.

### Remarks

Current specimens of *G. phractophallus* corresponded with the original description of the species [39]. Its occurrence on the gill lamellae of *S. fuscescens* in Moreton Bay represented a new fauna record for the bay.

## *Haliotrema* Johnston & Tiegs, 1922 (*sensu lato*)

### Remarks

*Haliotrema* (*sensu lato*) has been frequently shown to be non-monophyletic ([28, 78, 88, 89] among others] and to represent a group of morphologically similar species infecting a wide array of marine teleost fishes worldwide [44]; see also, World Register of Marine Species (WoRMS), at https://www.marinespecies.org/aphia.php?p=taxdetails&id=119284 for a list of species currently assigned to the genus. Unfortunately, a definition of the genus in the strict sense, *i.e.*, *Haliotrema* (*sensu stricto*), is lacking. As a result, the following five species are placed in *Haliotrema* (*sensu lato*), pending determination of the limits of the genus as a natural taxon.

## *Haliotrema apiculum* n. sp.

urn:lsid:zoobank.org:act:BCF6083A-21B9-48A3-8743-C35C82ACFCF8

Type host: Silver sweeper, *Pempheris schwenkii* Bleeker, Acropomatiformes, Pempheridae.

Type locality: Moreton Bay off Amity Point, North Stradbroke Island, Queensland, Australia (27°23′ S, 153°26′ E), 17 January 2016.

Infection site: Gill lamellae.

Specimens studied: Holotype, QM G240976; 26 paratypes, QM G240977–240987, AM W54754, USNM 1692983-1692990, HWML 217024; 15 vouchers, HWML 217025.

Etymology: The specific name (a noun) is from Latin (*apiculus* = a small point or spur) and refers to the small pointed spur at the proximal end of the deep root of the ventral anchor.

### Description (Figs. 43–50)

Body fusiform, flattened somewhat dorsoventrally. Cephalic region broad; two bilateral, two terminal cephalic lobes moderately to poorly developed. Eyespots absent; chromatic granules minute, ovate to subspherical; numerous granules scattered throughout cephalic region. Pharynx subspherical to ovate, with dorsoposterior indentation. Peduncle broad. Haptor subquadrate in outline, a simple extension of peduncle, poorly differentiated from body proper. Ventral anchor with broad base having elongate superficial root, wide deep root with small proximal spur directed toward superficial root; anchor shaft slightly arced, point recurved. Dorsal anchor robust, with broad base having comparatively long superficial root and short deep root, slightly arcing shaft, short straight point. Ventral bar rodshaped, with anteromedial shield flanked by two bilateral pockets. Dorsal bar robust, with expanded ends directed posteriorly. Hook distribution normal; each hook delicate, with short point, arcing shaft, protruding terminally depressed thumb, short shank comprised of single subunit; FH loop nearly shank length. Genital pore ventral, posterior to esophageal bifurcation. Testis ovate; proximal portion of vas deferens not observed, distal portion dilating to form seminal vesicle; seminal vesicle lying to left of MCO, folded into inverted U before giving rise to ejaculatory duct; ejaculatory duct entering base of MCO. Prostates, prostatic reservoirs not observed. MCO comprising slightly arcing shaft arising from base having short proximal flange; shaft of MCO terminally spatulate. Germarium subovate; oötype, Mehlis’ glands not observed; uterus delicate, directed anteriorly along body midline toward common genital pore. Vaginal pore dextromarginal; vaginal canal with thick wall distally giving rise to slightly expanded portion with wide ring before becoming tubular and uniting with seminal receptacle. Seminal receptacle spherical, lying immediately anterior to germarium. Vitellarium dense, coextensive with intestinal ceca; bilateral vitelline ducts extend toward body midline anterodorsal to seminal receptacle. Egg (observed in single specimen) ovate, with short robust proximal filament.

**Figures 43–50:**
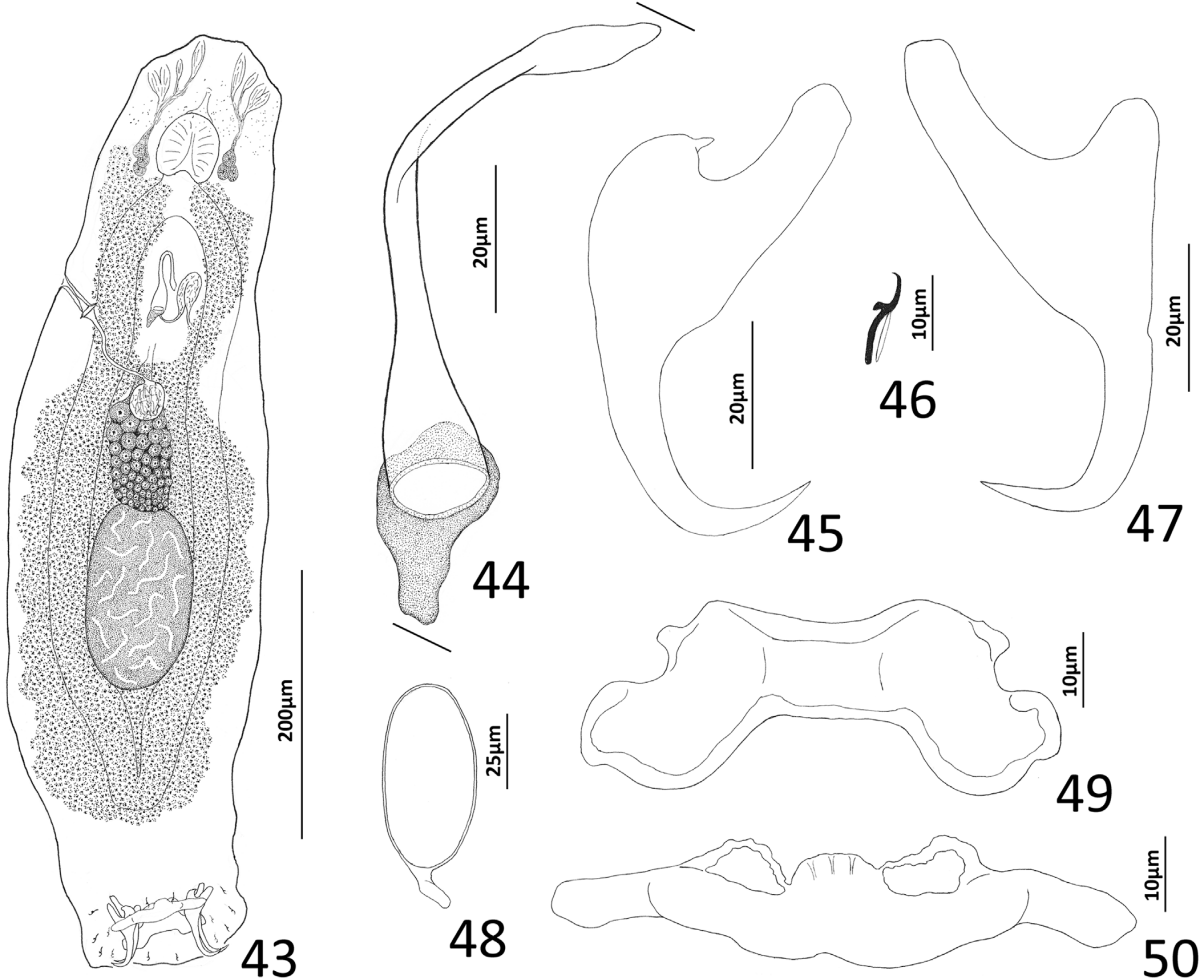
*Haliotrema apiculum* n. sp. from the silver sweeper, *Pempheris schwenkii*, Pempheridae. 43, Whole mount (ventral view, composite); 44, Male copulatory organ (ventral view); 45, Ventral anchor; 46, Hook; 47, Dorsal anchor; 48, Egg; 49, Dorsal bar; 50, Ventral bar. Parallel lines on Figure 44 indicate the limits of the dimension measured.

Measurements: Body 659–864 (744; *n* = 11) long; greatest width (excluding haptor) 173–227 (197; *n* = 12). Haptor 117–157 (135; *n* = 11) wide. Ventral anchor 57–69 (60; *n* = 12) long; dorsal anchor 57–64 (60; *n* = 12) long. Ventral bar 60–72 (67; *n* = 7) long; dorsal bar 61–73 (67; *n* = 13) long. Hook 12–14 (13; *n* = 29) long. Pharynx 51–74 (60; *n* = 12) long, 46–59 (54; *n* = 12) wide. Testis 124–171 (147; *n* = 12) long, 73–108 (86; *n* = 12) wide. Male copulatory organ 70–85 (77; *n* = 7) long. Germarium 68–98 (84; *n* = 11) long, 51–68 (57; *n* = 11) wide. Egg 57–58 (*n* = 1) long (excluding filament); 28–29 (*n* = 1) wide.

### Remarks

Four species of *Haliotrema* have previously been described parasitizing permpherid fishes in the western Pacific Ocean: *Haliotrema hatampo* Machida & Araki, 1977 from *Pempheris xanthoptera* Tominaga off southern Japan [55]; *Haliotrema flagellocirrus* Bychowsky & Nagibina, 1970 and *Haliotrema pempherii* Bychowsky & Nagibina, 1970, both from *Pempheris molucca* Cuvier in the South China Sea off Hainan, China [7]; and *Haliotrema umbraculiferum* Zhang, 2001 from *Pempheris oualensis* Cuvier off Huiyang (South China Sea), China [97]. The original descriptions of these species did not include whole-mount illustrations but were based primarily on the comparative morphology of the haptoral and copulatory sclerites. That *Haliotrema apiculum* n. sp. is morphologically close to these species is suggested by all five species possessing similar features of the haptoral armament and copulatory complex. The new species most closely resembles *H. hatampo* and *H. umbraculiferum* by having a simple arcing tubular shaft of the MCO. It differs from these species by having pockets on each side of an anteromedial shield in the ventral bar (pockets absent in *H. hatampo*) and by the presence of a small spur on the deep root of the ventral anchor (spur on the deep root of the ventral anchor lacking in both *H. hatampo* and *H. umbraculiferum*). The ventral bars of *H. flagellocirrus* and *H. pempherii* also have anterior pockets, but these species are easily differentiated from *H. apiculum* by having a branched shaft of the MCO in *H. flagellocirrus* (branches absent in *H. apiculum*), by the presence of fracture lines between the bases and shafts of the dorsal anchors in both *H. flagellocirrus* and *H. pempherii* (fracture lines absent in *H. apiculum*), and by lacking spurs on the deep roots of the ventral anchors (present in *H. apiculum*). *Haliotrema apiculum* differs from all other species currently assigned to *Haliotrema* (*sensu lato*) by having a spatulate tip of the MCO.

## *Haliotrema* cf. *dempsteri* (Mizelle & Price, 1964) Young, 1968

Syns *Parahaliotrema dempsteri* Mizelle & Price, 1964; *Haliotrema zancli* Yamaguti, 1968

(Figs. 51–56)

**Figures 51–56:**
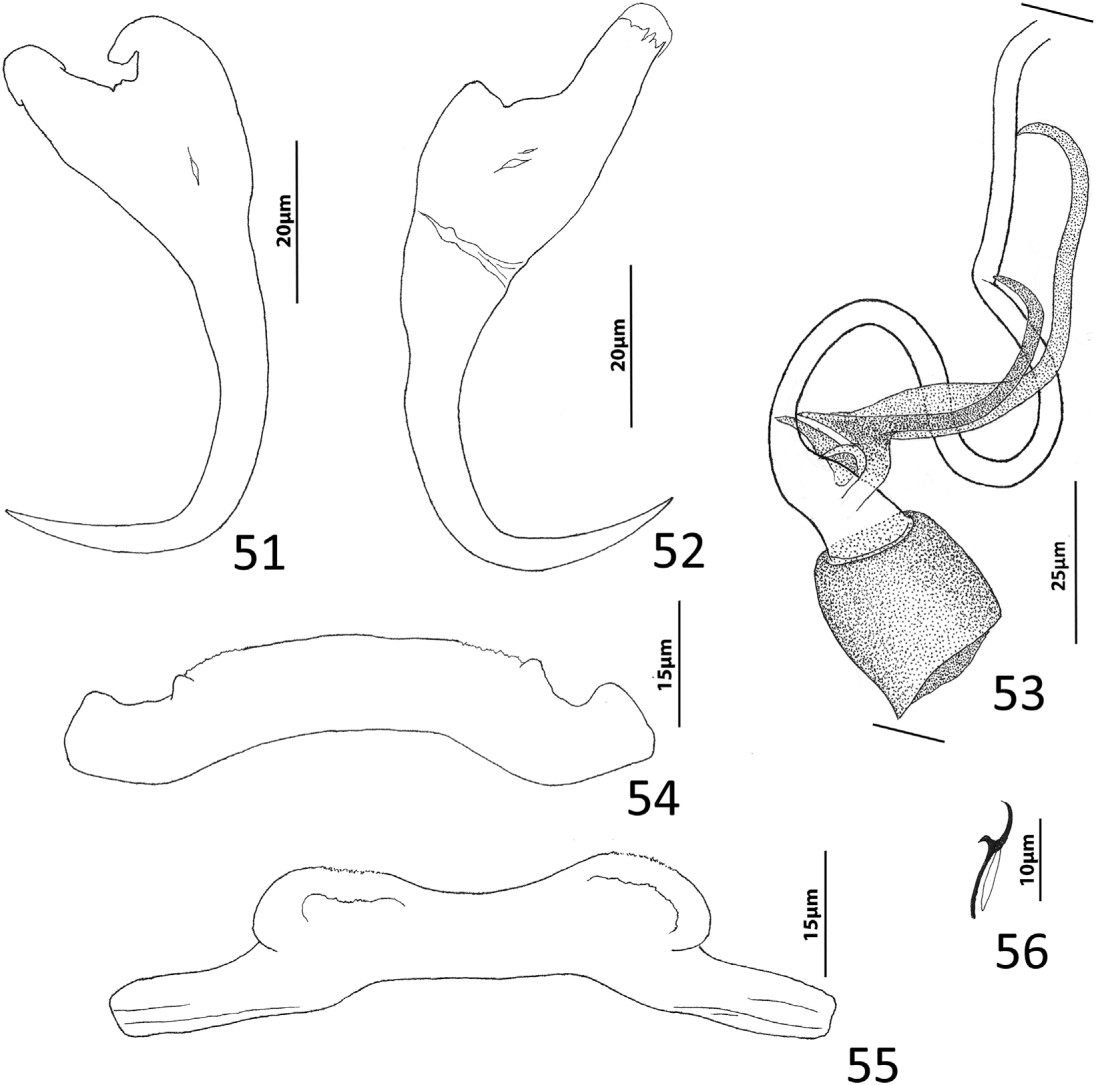
*Haliotrema* cf. *dempsteri* (Mizelle & Price, 1964) Young, 1968 from the sixplate sawtail, *Prionurus microlepidotus*, Acanthuridae. 51, Ventral anchor; 52, Dorsal anchor; 53, Copulatory complex (ventral view); 54, Dorsal bar; 55, Ventral bar; 56, Hook. Parallel lines on Figure 53 indicate the limits of the dimension measured.

Type host: Moorish idol, *Zanclus canescens* (Linnaeus) [now *Zanclus cornutus* (Linnaeus)], Acanthuriformes, Zanclidae.

Type locality: Southwest Pacific Ocean.

Current record: Sixplate sawtail, *Prionurus microlepidotus* Lacepède, Acanthuridae: Moreton Bay off Amity Point, Queensland, Australia (27°23′ S, 153°26′ E), 21 January 2016.

Previous records: *Z. cornutus* (as *Z. canescens*): Southwest Pacific Ocean [60]; Hawaii [92 (as *Haliotrema zancli*). *Acanthurus dussumieri* Valenciennes, Acanthuridae: Heron Island, Queensland, Australia [95]; Hawaii [92] (as *Haliotrema zancli*). *Acanthurus olivaceus* Bloch & Schneider, Acanthuridae: Hawaii [92] (as *Haliotrema zancli*). *Acanthurus mata* (Cuvier), Acanthuridae: Heron Island, Queensland, Australia [95]); Hawaii [92] (as *Haliotrema zancli*). *Acanthurus xanthopterus* Valenciennes, Acanthuridae: Green Island off Cairns (approximately 16.76°S, 145.97°E; see [41] for information on the locality), Queensland, Australia [95]); Heron Island, Queensland, Australia [95]; Central Pacific Ocean off Palmyra Atoll (27°23′ S, 153°26′ E), USA [85]. *Ctenochaetus strigosus* (Bennett), Acanthuridae: Hawaii ([92] as *Haliotrema zancli*).

Infection site: Gill lamellae.

Specimens studied: 11 voucher specimens, QM G241021–241024; AM W54757; USNM 1693013-1693016; HWML 217028.

Museum specimens examined: Holotype, *Parahaliotrema dempsteri* Mizelle & Price, 1964, USNM 1355798; paratype, *P. dempsteri*, USNM 1355798; voucher specimen, *Haliotrema dempsteri* (from *Acanthurus mata*, Australia), USNM 1356988; 3 voucher specimens, *H. dempsteri* (from *Acanthurus dussumieri*, Australia), USNM 1356989; voucher specimen, *H. dempsteri* (from *Acanthurus xanthopterus*, Palmyra Atoll), USNM 1459846; holotype, numerous paratypes, *Haliotrema zancli* Yamaguti, 1968 (from *Zanclus canescens* [now *Z. cornutus*], Hawaii), USNM 1359349; holotype, several paratypes, *Pseudohaliotrematoides zancli* Yamaguti, 1968 (from *Z. canescens*, Hawaii), USNM 1359361 (this slide contained many specimens of *H. zancli* which were used below for the measurements of the haptoral sclerites, see Remarks); holotype, paratype*, Parahaliotrema zebrasoma* Mizelle & Kritsky, 1969 (from *Zebrasoma velifera*, Hawaii), USNM 1366553; holotype, paratype, *Haliotrema ctenochaeti* Young, 1968 (from *Ctenochaetus strigosus*, Australia), USNM 1356991, 1356992; holotype, *Haliotrema ctenochaeti* Yamaguti, 1968, USNM 1359329 (from *C. strigosus*, Hawaii); holotype, *Haliotrema sigmoidocirrus* Yamaguti, 1968 (from *A. mata*, Hawaii), USNM 1359345.

Measurements: Body 512–633 (585; *n* = 4) long; greatest width (excluding haptor) 125–163 (142; *n* = 5). Haptor 135–149 (142; *n* = 5) wide. Ventral anchor 58–64 (62; *n* = 4) long; dorsal anchor 64–70 (68; *n* = 4) long. Ventral bar 84–90 (87; *n* = 4) long; dorsal bar 69–74 (72; *n* = 4) long. Hook 13–15 (14; *n* = 8) long. Pharynx 35–40 (38; *n* = 4) long, 35–40 (38; *n* = 10) wide. Testis 79–101 (92; *n* = 5) long, 49–72 (60; *n* = 5) wide. Copulatory complex 90–128 (113; *n* = 5) long. Germarium 35–54 (44; *n* = 5) long, 34–50 (40; *n* = 5) wide.

### Remarks

Since its description as *Parahaliotrema dempsteri* from the zanclid, *Zanclus canescens* (now *Z. cornutus*), in the western Pacific Ocean [60], the helminth has been reassigned to *Haliotrema* as *Haliotrema dempsteri* (Mizelle & Price, 1964) Young, 1968 and reported as a parasite of acanthurid fishes, including *Acanthurus dussumieri* and *A. mata* in Australia [95] and *A. xanthopterus* [85, 95] from Australia and the Palmyra Atoll, respectively. The occurrence of the species on *Prionurus microlepidotus* (Acanthuridae) and the placement herein of *Haliotrema zancli* Yamaguti, 1968 from *Z. canescens* (type host) and the acanthurids *A. dussumieri*, *A. olivaceus*, *A. mata* and *Ctenochaetus strigosus* as a junior subjective synonym of *H. dempsteri* (see below), further indicated a comparatively low host specificity for the helminth.

Examination of the museum specimens listed above revealed that the morphological features of the haptoral and copulatory sclerites of the specimens identified as *H. dempsteri* from the acanthurid hosts did not differ significantly from those of specimens from *Z. cornutus*, supporting the conspecificity of these helminths. The morphological differences among specimens from the various hosts were likely due to intraspecific variation and/or the orientations of structures of individual specimens within the microscopic plane of view. What did appear to be potentially important were the metrical differences of the haptoral sclerites among specimens from the various host species, where dimensions of the sclerites of specimens collected independently by Mizelle & Price [60] and Yamaguti [92] from *Z. cornutus* were comparable but noticeably smaller than those of specimens from acanthurid hosts (Table 2). While the differences in metrics among the parasites infecting different hosts may be a result of variables associated with methodology and/or the macro- and host environments in which the parasites occur, they might indicate that a species complex exists.

**Table 2:** Comparative measurements (in micrometers) of haptoral sclerites by host and locality of specimens identified herein as *Haliotrema* cf. *dempsteri*.

Host	*Prionurus microlepidotus*	*Zanclus cornutus*	*Zanclus cornutus*	*Acanthurus xanthopterus*	*Acanthurus mata*	*Acanthurus dussumieri*
Locality	Moreton Bay, Australia	Western Pacific Ocean	Hawaii, USA	Palmyra Atoll, USA	Hawaii, USA	Heron Island, Australia
Reference	[*ex nobis*]	[60]	[92]*	[85]	[92]	[95]
Museum Numbers**	QM G241021-024; USNM 1693013-016; HWML 217028; AM W54757	USNM 1355798	USNM 1359349, 1359361	USNM 1459846	USNM 1356988	USNM 1356989
Ventral anchor						
Length	58–64 (62; *n* = 4)	36–37 (*n* = 2)	35–39 (37; *n* = 10)	52–53 (*n* = 1)	69–70 (*n* = 1)	66–92 (75; *n* = 3)
Dorsal anchor						
Length	64–70 (68; *n* = 4)	38–42 (40; *n* = 2)	38–43 (41; *n* = 11)	60–61 (*n* = 1)	81–82 (*n* = 1)	67–114 (90; *n* = 2)
Ventral bar						
Length	84–90 (87; *n* = 4)	45–49 (47; *n* = 2)	44–48 (46; *n* = 9)	61–62 (*n* = 1)	67–68 (*n* = 1)	77–80 (79; *n* = 2)
Dorsal bar						
Length	69–74 (72; *n* = 4)	40–43 (42; *n* = 2)	39–46 (43; *n* = 11)	47–48 (*n* = 1)	59–60 (*n* = 1)	66–76 (71; *n* = 3)
Hook						
Length	13–15 (14; *n* = 8)	11–12 (*n* = 2)	11–13 (12; *n* = 10)	14–15 (*n* = 1)	13–14 (*n* = 1)	12–15 (14; *n* = 3)

*Helminth specimens reported by Yamaguti [91] from Hawaii were originally described as *Haliotrema zancli* Yamaguti, 1968, but considered herein to represent a junior subjective synonym of *H. dempsteri*.

**QM = Queensland Museum; USNM = Smithsonian Institution, Invertebrate Zoology Collection; HWML = Harold W. Manter Laboratory; AM = Australian Museum.

Although far from a comprehensive survey, museum specimens of three other *Haliotrema* spp. infecting acanthurid hosts were also examined. These species included *Haliotrema zebrasoma* (Mizelle & Kritsky, 1969) Vala, Maillard, & Overstreet, 1982; *Haliotrema sigmoidocirrus* Yamaguti, 1968; and *Haliotrema ctenochaeti* Young, 1968, all of which possessed haptoral and copulatory sclerites morphologically similar to those of *H. dempsteri* [58, 92, 95], suggesting further that *H. dempsteri* and some of the other species infecting acanthurid hosts may represent a species complex, with the helminths infecting *Z. cornutus* representing *H. dempsteri* (*sensu stricto*). A determination of whether a species complex does exist, however, may depend on comparison of molecular data from dactylogyrids infecting zanclids and various acanthurid hosts. The present specimens are therefore conditionally assigned to the species as *Haliotrema* cf. *dempsteri*.

As a result of the examination of the holotype and paratype of *Parahaliotrema dempsteri* Mizelle & Price, 1964 (now *H. dempsteri*) and the holotype and numerous other specimens of *Haliotrema zancli* Yamaguti, 1968 (all from *Z. cornutus*) and based on the comparative morphology of the copulatory organs and haptoral sclerites of the respective specimens, it became apparent that the two species are synonyms, with the former having priority. Thus, *H. zancli* Yamaguti, 1968 is placed in junior subjective synonymy with *H. dempsteri* (Mizelle and Price, 1964) Young, 1968. Yamaguti [92] had previously suspected but did not formally propose the synonymy of the two species (see the footnote on page 94 of Yamaguti [92]). Specimens of *H. zancli* reported by Yamaguti [92] from the acanthurid hosts were not available for study.

Yamaguti [92] described *Pseudohaliotrematoides zancli* Yamaguti, 1968 from the gills of *Z. cornutus* from Hawaii. Based on the examination of the type specimens of *P. zancli*, the species is here transferred to *Haliotrema* (*sensu lato*) as *Haliotrema hawaiiense* n. nom. *Pseudohaliotrematoides* Yamaguti, 1953 was placed as a junior subjective synonym of *Tetrancistrum* Goto & Kikuchi, 1917 by Kritsky *et al.* [38]. At that time (2007), *Pseudohaliotrematoides* contained six species, three of which were considered “species inquirenda” and the other three, *Pseudohaliotrematoides falcatus* Yamaguti, 1968, *Pseudohaliotrematoides recurvatus* Yamaguti, 1968, and *Pseudohaliotrematoides zancli* Yamaguti, 1968, were not transferred but retained in the invalid *Pseudohaliotrematoides* as species with uncertain taxonomic positions [38]. As a result of the transfer of *P. zancli* to *Haliotrema*, renaming of the species as *H. hawaiiense* n. nom. was necessary to avoid homonymy of the species with *Haliotrema zancli* Yamaguti, 1968, the description of which occurred earlier than that of *P. zancli* within Yamaguti’s [92] book.

The present survey of the *Haliotrema* spp. that are morphologically similar to *H. dempsteri* also included the examination of the type specimens of *H. ctenochaeti* Young, 1968 and *H. ctenochaeti* Yamaguti, 1968, which showed that both represented valid species of *Haliotrema* (*sensu lato*) (compare figure 2 in Young [95] and figure 49 in Yamaguti [92]). As a result, the two species are homonyms, which required the re-naming of the junior taxon. *Haliotrema ctenochaeti* Young, 1968 has priority over *H. ctenochaeti* Yamaguti, 1968 based on Article 21.3 of the International Code of Zoological Nomenclature (ICZN) [22]. Thus, *H. ctenochaeti* Yamaguti, 1968 is renamed *H. asymphylum* n. nom. The specific name, an adjective, was derived from Greek (asymphylus = not of the same race).

The finding of *H. dempsteri* on *Prionurus microlepidotus* represented new host and geographical records for the helminth and a new faunal record for Moreton Bay.

## *Haliotrema johnstoni* Bychowsky & Nagibina, 1970

Type host: Freckled goatfish, *Upeneus tragula* Richardson, Mulliformes, Mullidae.

Type locality: South China Sea of Hainan, China.

Current record: *U. tragula*: Moreton Bay off Amity Point, Queensland, Australia (27°23′ S, 153°26′ E), 18 January 2016.

Previous records: *U. tragula*: South China Sea [7, 97]. *Upeneus luzonius* Jordon & Seale, Mullidae: Haikou, Hainan Province, China [88, 97]. *Parupeneus chrysopleuron* (Temminck & Schlegel): China [97].

Infection site: Gill lamellae.

Specimens studied: 15 voucher specimens, QM G240967–240971, AM W54758, USNM 1692970-1692975, HWML 217022.

Museum specimens examined: 2 cotypes, *Haliotrema australe* Johnston & Tiegs, 1922, AM W 883.001.

### Redescription (Figs. 57–63)

Body flattened dorsoventrally, with nearly parallel lateral margins. Tegument smooth except for a dextrolateral zone of papillae-like folds on the trunk immediately posterior to vaginal pore. Cephalic region broad; cephalic lobes moderately developed. Two pairs of eyespots, anterior pair occasionally dissociated; members of posterior pair larger, slightly farther apart than those of anterior pair; lenses apparently absent; chromatic granules minute, ovate; accessory granules sparse in cephalic region. Pharynx subspherical to ovate, with indentation of posterior end; esophagus short to absent; intestinal ceca dilated, confluent posterior to testis. Peduncle broad, tapered posteriorly. Haptor subhexagonal in outline, with lateral lobes containing hook pairs 3, 4, 6. Ventral and dorsal anchors similar in shape; each anchor robust, with large base having elongate superficial root and well-developed but shorter deep root, slightly curved shaft and point; deep roots of ventral and dorsal anchors with spine-like termination directed toward tip of superficial root; spine of dorsal anchor small, often inconspicuous. Ventral bar gently arced or broadly V shaped, with rounded slightly enlarged ends; dorsal bar with bifurcated ends. Hook distribution normal; each hook with protruding terminally depressed thumb; FH loop nearly shank length. Testis subspherical to ovate. Proximal portion of vas deferens not observed, distal portion dilating to form small fusiform seminal vesicle; seminal vesicle lying to left of body midline; ejaculatory duct entering base of MCO. Prostates not observed; single prostatic reservoir elongate, often sigmoid, lying dorsal to copulatory complex, with distal duct entering base of MCO (Bychowsky & Nagibina, 1970 observed two prostatic reservoirs). Copulatory complex comprising articulated MCO and accessory piece. MCO with large inverted-cup-shaped base and lightly sclerotized shaft; accessory piece lightly sclerotized, with rounded distal end. Germarium pyriform; oötype not observed, apparently lying dorsal to seminal receptacle; Mehlis’ glands not observed; uterus delicate, minimally dilated, extending anteriorly along body midline. Vaginal pore dextromarginal; unsclerotized vagina canal arising from seminal receptacle, meandering to large thick-walled vaginal vestibule. Seminal receptacle spherical, lying on body midline immediately anterior to germarium. Vitellarium dense, coextensive with intestinal ceca; bilateral vitelline ducts extend toward body midline at level of seminal receptacle. Egg not observed.

**Figures 57–63:**
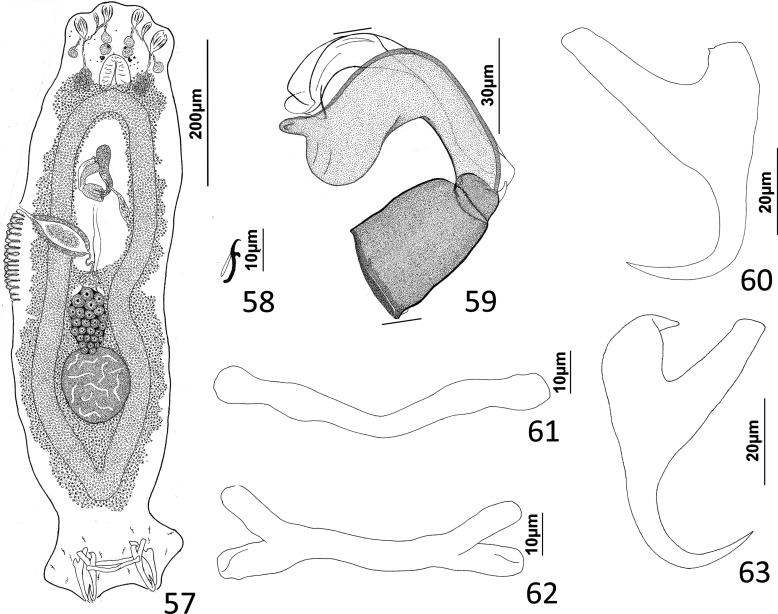
*Haliotrema johnstoni* Bychowsky & Nagibina, 1970 from the freckled goatfish, *Upeneus tragula*, Mullidae. 57, Whole mount (ventral view, composite); 58, Hook; 59, Copulatory complex (dorsal view); 60, Dorsal anchor; 61, Ventral bar; 62, Dorsal bar; 63, Ventral anchor. Parallel lines on Figure 59 indicate the limits of the dimension measured.

Measurements (respective measurements from the original description [7] follow in brackets those of current specimens): Body 660–822 (726; *n* = 11) [640–1,000] long; greatest width (excluding haptor) 169–223 (190; *n* = 12) [160–230]. Haptor 125–186 (163; *n* = 7) wide. Ventral anchor 55–58 (57; *n* = 3) [53–60] long; dorsal anchor 59–64 (62; *n* = 3) [60–65] long. Ventral bar 63–72 (69; *n* = 3) [68–72] long; dorsal bar 63–71 (66; *n* = 3) [62–70] long. Hook 10–12 (11; *n* = 5) [10–12] long. Pharynx 43–54 (49; *n* = 10) long, 39–45 (42; *n* = 10) wide [Pharyngeal diameter 36–44]. Testis 88–130 (105; *n* = 11) long, 46–99 (82; *n* = 10) [103–136 × 58–122] wide. Copulatory complex 85–94 (90; *n* = 3) [~100] long. Germarium 58–76 (67; *n* = 11) long, 41–67 (59; *n* = 10) [41–50 × 58–122] wide.

### Remarks

The redescription of *H. johnstoni* is provided as a complement to the original description of the species by Bychowsky & Nagibina [7]; specimens from Moreton Bay closely conform with the original description. Whereas the dextral tegumental patch of papilliform folds of the tegument was clearly evident in stained specimens from Moreton Bay, it was less obvious in unstained specimens mounted in Gray & Wess medium, where it often appeared as detritus occurring along the right side of the trunk. Bychowsky & Nagibina [7] also depicted the patch in their whole-mount drawing where it was shown to be well posterior of the vaginal pore and its extent considerably less than that of specimens from Moreton Bay (compare Fig. 57 with figure 1 in Bychowsky & Nagibina [7]). For clarification, the labels for the whole-mount figures of *H. johnstoni* and *Haliotrema australe* Johnston & Tiegs, 1922 were reversed in the plate provided by Bychowsky & Nagibina [7].

The occurrence of *H. johnstoni* in Moreton Bay represents a new faunal record for the bay.

## *Haliotrema spirale* Yamaguti, 1968

(Figs. 64–69)

**Figures 64–69:**
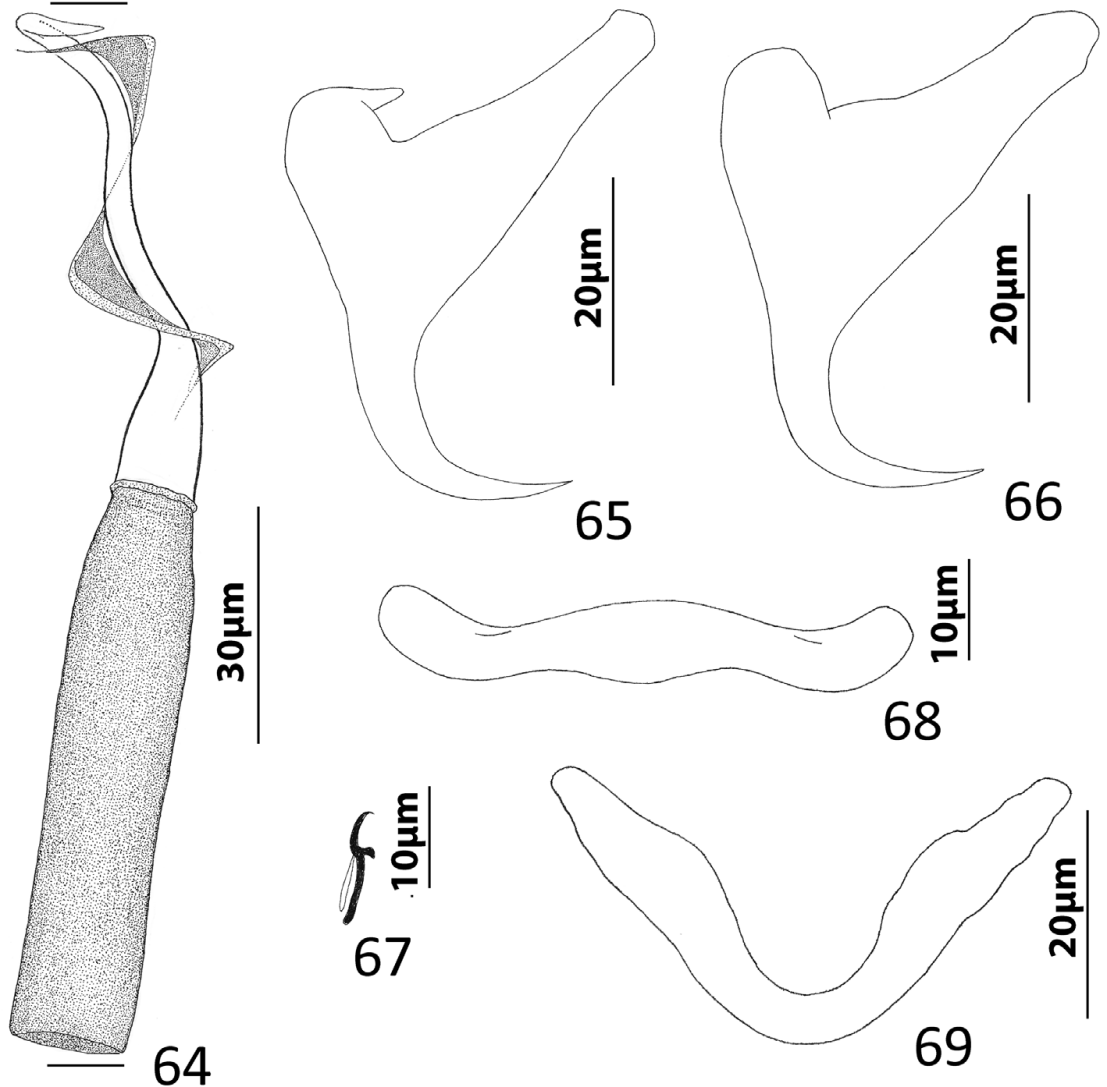
*Haliotrema spirale* Yamaguti, 1968 from the freckled goatfish, *Upeneus tragula*, Mullidae. 64, Male copulatory organ (dorsal view); 65, Ventral anchor; 66, Dorsal anchor; 67, Hook; 68, Dorsal bar; 69, Ventral bar. Parallel lines on Figure 64 indicate the limits of the dimension measured.

Type host: Orange goatfish, *Mulloidichthys pfluegeri* (Steindachner), Mulliformes, Mullidae.

Type locality: Hawaii.

Current record: *Upeneus tragula* Richardson, Mullidae: Moreton Bay off Amity Point, Queensland, Australia (27°23′ S, 153°26′ E), 18 January 2016.

Previous records: *M. pfluegeri*: Hawaii [92]. *Mulloidichthys auriflamma* (Forsskål) [now *Parupeneus forsskali* (Fourmanoir & Guézé)]: Hawaii [92]. *Mulloidichthys samoensis* (Günther) (now *Mulloidichthys flavolineatus* (Lacepède): Hawaii [92]. *Parupeneus pleurostigma* (Bennett): Hawaii [92]. *Parupeneus chryserydros* (Lacepède) [now *Parupeneus cyclostomus* (Lacepède)]: Hawaii [92].

Infection site: Gill lamellae.

Specimens studied: 15 voucher specimens, QM G240972–240975, AM W54756, USNM 1692976-1692982, HWML 217023.

Measurements: (respective measurements from the original description follow in brackets those of current specimens): Body 499–586 (538; *n* = 10) [600–870] long; greatest width (excluding haptor) 131–175 (155; *n* = 10) [120–220]. Haptor 116–132 (124; *n* = 5) [110] wide. Ventral anchor 43–48 (46; *n* = 5) [45–75] long; dorsal anchor 45–49 (47; *n* = 4) [60–82] long. Ventral bar 44–49 (46; *n* = 2) [52–75] long; dorsal bar 46–51 (49; *n* = 3) [45–61] long. Hook 10–12 (11; *n* = 14) [10] long. Pharynx 37–48 (42; *n* = 9) long, 38–46 (42; *n* = 9) wide [pharyngeal diameter 30–40]. Testis 75–113 (90; *n* = 9) long, 64–103 (80; *n* = 8) [80–180 × 50–130]. Copulatory complex 123–137 (130; *n* = 6) [150–190] long. Germarium 46–66 (52; *n* = 9) long, 30–49 (44; *n* = 8) [46–95 × 28–82] wide.

### Remarks

Although body lengths of the specimens of *H. spirale* from Australia were slightly less than those of specimens from Hawaii, present specimens conformed with the description of the species by Yamaguti [92]. Two differences were noted: 1) the constriction of the body at the level of the vagina, and 2) the muscle pads located anterior to the anchor/bar complexes, both mentioned by Yamaguti [92], were not observed in the specimens from Australia. These differences may have been a result of different collection, staining, and mounting techniques used in the two studies.

*Haliotrema spirale* was originally described from five mullid hosts from Hawaii by Yamaguti [92]. Its occurrence on these hosts in Hawaii and now on *U. tragula* in Australia suggested that the helminth may be a ubiquitous parasite of mullid fishes throughout the Indo-Pacific region. The occurrence of *H. spirale* on *U. tragula* in Moreton Bay represented a new host record for the parasite and a new faunal record for the bay.

## *Haliotrema tugulduriforme* n. sp.

urn:lsid:zoobank.org:act:35F3D5E5-B50D-46BA-A85D-AAF6B23FD0E9

Type host: Blue-barred parrotfish, *Scarus ghobban* Fabricius, Series Eupercaria, Scaridae.

Type locality: Moreton Bay off Amity Point, North Stradbroke Island, Queensland, Australia (27°23′ S, 153°26′ E), 11 January 2016.

Infection site: Gill lamellae.

Specimens studied: Holotype, QM G240988; 23 paratypes, QM G240989–240999, AM W54755, USNM 1692991-1692997, HWML 217026.

Etymology: The specific name (treated as an adjective) refers to the base of the copulatory complex resembling a Mongolian tuguldur hat.

### Description (Figs. 70–76)

Body fusiform, flattened dorsoventrally. Cephalic region broad; cephalic lobes moderately to poorly developed. Eyespots usually absent, rarely present as one or two pairs of poorly organized chromatic granules; chromatic granules minute, spherical; numerous granules scattered in cephalic region. Pharynx subspherical to ovate, with dorsoposterior indentation; esophagus moderately long; intestinal ceca united well posterior to testis. Peduncle broad, slightly tapered toward haptor. Haptor subhexagonal in outline. Anchors similar; each with broad base having elongate superficial root and short deep root, slightly arcing shaft, short recurved point; superficial root of dorsal anchor noticeably longer than that of ventral anchor. Ventral bar appearing as two bilateral wings, each with ventral pockets; pockets may be continuous at midlength of bar. Dorsal bar broadly V shaped, with slightly expanded rounded ends. Hook distribution normal; each hook delicate, with short point, arcing shaft, protruding terminally depressed thumb, short shank comprised of single subunit; FH loop nearly shank length. Genital pore midventral, posterior to esophageal bifurcation. Testis ovate; proximal portion of vas deferens not observed, distal portion dilating to form seminal vesicle; seminal vesicle lying to left of MCO, folded into inverted U before giving rise to ejaculatory duct; ejaculatory duct apparently entering base of MCO. Prostates not observed; single prostatic reservoir lying to left of MCO. MCO comprising slightly arcing shaft arising from base; base resembling a Mongolian tuguldur hat; shaft of MCO with whip-like termination. Accessory piece a sinuous rod basally articulated to base of MCO. Germarium ovate; oötype, Mehlis’ glands not observed; uterus delicate, directed anteriorly along body midline toward common genital pore. Vaginal pore dextromarginal; vaginal canal with thick distal wall, proximally giving rise to delicate meandering proximal segment uniting with seminal receptacle. Seminal receptacle spherical, lying immediately anterior to germarium. Vitellarium dense, coextensive with intestinal ceca; bilateral vitelline ducts extend toward body midline dorsal to seminal receptacle. Egg not observed.

**Figures 70–76:**
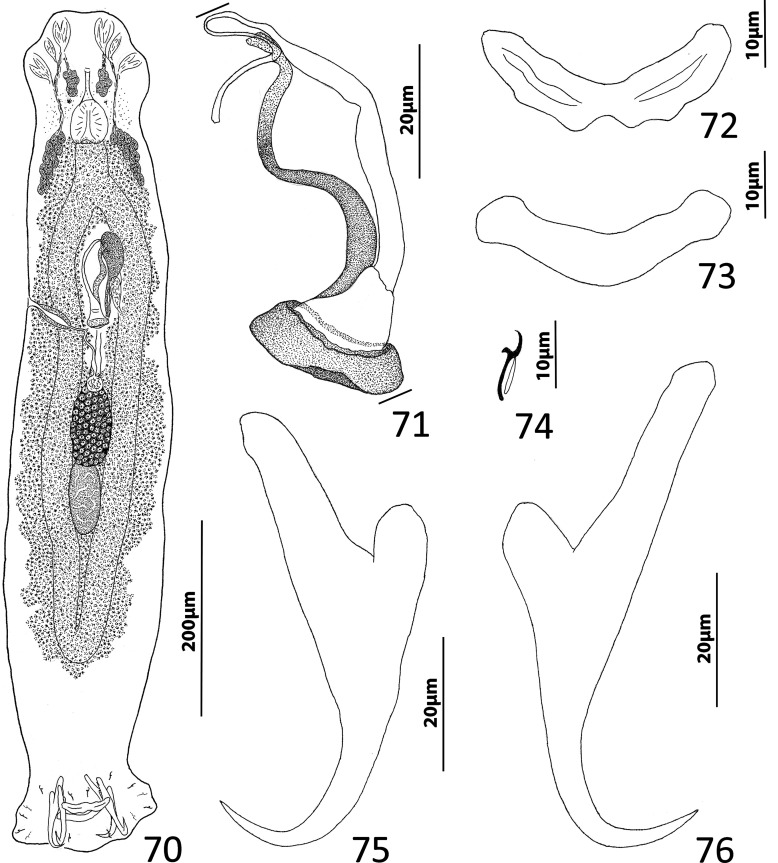
*Haliotrema tugulduriforme* n. sp. from the blue-barred parrotfish, *Scarus ghobban*, Scaridae. 70, Whole mount (ventral view, composite); 71, Copulatory complex (dorsal view); 72, Ventral bar; 73, Dorsal bar; 74, Hook; 75, Ventral anchor; 76, Dorsal anchor. Parallel lines on Figure 71 indicate the limits of the dimension measured.

Measurements: Body 716–989 (802; *n* = 11) long; greatest width (excluding haptor) 130–161 (143; *n* = 13). Haptor 95–149 (122; *n* = 13) wide. Ventral anchor 68–72 (70; *n* = 10) long; dorsal anchor 72–83 (77; *n* = 10) long. Ventral bar 39–49 (45; *n* = 10) long; dorsal bar 41–49 (45; *n* = 9) long. Hook 12–14 (13; *n* = 16) long. Pharynx 44–55 (49; *n* = 12) long, 40–50 (45; *n* = 13) wide. Testis 54–93 (68; *n* = 11) long, 29–39 (35; *n* = 11) wide. Copulatory complex 62–81 (71; *n* = 10) long. Germarium 62–101 (76; *n* = 11) long, 43–56 (49; *n* = 11) wide.

### Remarks

Five species of *Haliotrema* have been described from scarid fishes: *Haliotrema conspecta* Zhukov, 1980 from the redtail parrotfish, *Sparisoma chrysopterum* (Block & Schneider); *Haliotrema sanchezae* Cruces, Chero, Saez, & Luque, 2017 from the bumphead parrotfish, *Scarus perrico* Jordan & Gilbert; *Haliotrema scari* Young, 1968 from the rivulated parrotfish, *Scarus fasciatus* Valenciennes (now *Scarus rivulatus* Valenciennes); *Haliotrema shanweii* Li, 2007 from *Scarus* sp.; and *Haliotrema tuberobaculum* Zhukov, 1980 from the striped parrotfish, *Scarus croicensis* Bloch [now *Scarus iseri* (Bloch)]. Based on the comparative morphology of the copulatory complexes, *Haliotrema tugulduriforme* n. sp. most closely resembles *H. scari* and *H. shanweii* by possessing a copulatory complex with an accessory piece articulated to the tubular MCO [47, 95]. It differs from these species by the position of the articulation point of the MCO and accessory piece (articulation point basal in *H. tugulduriforme* and near the midlength of the MCO in *H. scari* and *H. shanweii*). The remaining species, all occurring on scarids from the marine waters of the Western Hemisphere, have a cylindrical MCO enveloped by a sheath [11, 99].

The occurrence of species having two distinct types of copulatory complexes in the western Pacific Ocean and the Western Hemisphere, respectively suggests that at least two evolutionary clades may exist among the species of *Haliotrema* from scarids worldwide. That two clades may exist is supported by the species of *Haliotrema* occurring on scarids from the western Pacific Ocean having dorsal and ventral haptoral bars lacking bifurcated ends, whereas one or both of the haptoral bars of species occurring in the Western Hemisphere have bifurcated ends.

## *Hamatopeduncularia thalassini* Bychowsky & Nagibina, 1969

Syn. *Hamatopeduncularia thalissini* of Rastogi *et al.* (2005) (misspelling)

(Figs. 77–82)

**Figures 77–82:**
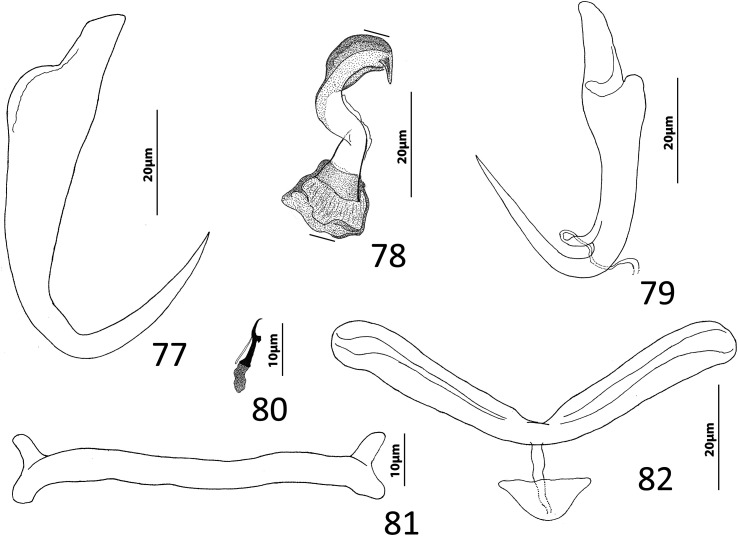
*Hamatopeduncularia thalassini* Bychowsky & Nagibina, 1969 from the Arafura catfish, *Pararius proximus*, Ariidae. 77, Dorsal anchor; 78, Copulatory complex (ventral view); 79, Ventral anchor; 80, Hook; 81, Ventral bar; 82, Dorsal bar. Parallel lines on Figure 78 indicate the limits of the dimension measured.

Type host: Giant catfish, *Arius thalassinus* (Rüppell) [now *Netuma thalassina* (Rüppell)], Siluriformes, Ariidae.

Type locality: South China Sea of Hainan, China.

Current record: Arafura catfish, *Pararius proximus* (Ogilby), Ariidae: Moreton Bay off Green Island, Queensland, Australia (27°25′ S, 153°14′ E), 11 January 2016.

Previous records: *N. thalassina* (all as *A. thalassinus*): South China Sea of Hainan, China [5]; Straits of Malacca (Sungai Buloh, Selangor), Peninsular Malaysia [51]; Probandar, India [64]. *Arius jella* Day: Off Visakhapatnam Coast, Bay of Bengal, Andhra Pradesh, India (27°30′ S, 153°20′ E) [21]. *Arius* sp., Siluriformes, Ariidae: Probandar, India [64]. *Mystus seenghala* (Sykes) [now *Sperata seenghala* (Sykes)], Siluriformes, Bagridae: Ganges River at Chandpur, India [65].

Infection site: Gill lamellae.

Specimens studied: 14 voucher specimens, QM G240960–240966, USNM 1692966-1692969, HWML 217021.

Measurements (respective measurements from the original description follow in brackets those of current specimens): Body 921–1,090 (1,030; *n* = 4) [580–660] long; greatest width (excluding haptor) 102–138 (123; *n* = 4) [70–80]. Haptor 106–135 (120; *n* = 4) wide. Ventral anchor 52–57 (54; *n* = 10) [55] long; dorsal anchor 62–70 (65; *n* = 8) [68] long. Ventral bar 62–81 (73; *n* = 7) [70)] long; dorsal bar 72–90 (83; *n* = 7) [90–100] long. Hook 14–19 (16; *n* = 19) [10–12] long. Pharynx 53–70 (59; *n* = 4) long, 48–54 (51; *n* = 4) wide. Testis 118–146 (134; *n* = 3) long, 52–61 (55; *n* = 3) wide. Copulatory complex 87–106 (97; *n* = 7) [50] long. Germarium 87–105 (94; *n* = 3) long, 40–54 (49; *n* = 3) wide.

### Remarks

The specimens of *H. thalassini* from *P. proximus* in Moreton Bay were identified based on the original description of the species. Present specimens conformed relatively closely with the original description, except that they tended to be somewhat larger than those described by Bychowsky & Nagibina [5] (see measurements). In addition, the medio-posterior process of the dorsal bar was found to be hood-like in specimens from Moreton Bay, whereas it was shown in the original description to resemble the T-shaped hand tool known as a pickax in the original description. Finally, a filamentous extension of the distal spine of the ventral-anchor shaft was observed in the present specimens (Fig. 79), in one specimen of which it appeared to be continuous with that of the other member of the anchor pair.

The occurrence of *H. thalassini* on *P. proximus* in Moreton bay represents a new host record for the helminth and a new faunal record for the bay.

## *Lethrinitrema australiense* n. sp.

urn:lsid:zoobank.org:act:D7ABAB43-201B-45A0-88F5-51B58A10F0B3

Type host: Spangled emperor, *Lethrinus nebulosus* (Forsskål), Series Eupercaria, Lethrinidae.

Type locality: Moreton Bay off Peel Island, Queensland, Australia (27°30′ S, 153°20′ E), 13 January 2016.

Infection site: Gill lamellae.

Specimens studied: Holotype, QM G240865; 23 paratypes, QM G240866–240874, USNM 1692885-1692893, HWML 217004.

Etymology: The specific name is derived by adding the Latin suffix (-*ensis* = denoting place) to the country name and refers to the species occurring in the marine waters off Australia.

### Description (Figs. 83–89)

Body elongate, with nearly parallel lateral margins, flattened dorsoventrally. Cephalic region broad; terminal and two bilateral cephalic lobes moderately to well developed. Two pairs of eyespots, members often dissociated, apparently lacking lenses; members of posterior pair slightly larger and nearly equidistant with those of anterior pair; chromatic granules minute, ovate to irregular; accessory granules uncommon or absent in cephalic region. Pharynx ovate; esophagus short to non-existent; intestinal ceca terminate blindly anterior to haptor, union of ceca posterior to testis suggested but unconfirmed. Peduncle broad, short to non-existent. Haptor subhexagonal, with two small reservoirs emptying near bases of anchors *via* delicate ducts. Ventral anchor robust, with short arcing shaft, moderately long recurved point ending short of level of tip of superficial root; ventral anchor base with elongate deep and superficial roots; superficial root having terminal bulbous lobe. Dorsal anchor delicate, with elongate superficial root, shorter deep root, short arcing shaft, long recurved point extending short of level of tip of superficial root. Ventral bar rod shaped, with paired submedial expansions along anterior margin; dorsal bar broadly V shaped. Hook distribution normal; each hook with protruding, blunt, terminally depressed thumb, uniform shank comprised of single subunit; FH loop nearly shank length. Testis ovate; proximal portion of vas deferens not observed, distal portion directed anteriorly, recurving posteriorly sinistral to MCO, then entering base of MCO. Seminal vesicle, prostates, prostatic reservoirs not observed. MCO with large base shaped as inverted Mexican tequila shot glass (el Caballito) and tubular shaft proximally tapered, shaped as interrogation mark. Germarium pyriform; oötype, Mehlis’ glands not observed; uterus delicate, extending anteriorly along body midline, occasionally containing single egg. Vaginal pore dextral, submarginal; vagina unsclerotized, enclosed within thick sleeve. Seminal receptacle subspherical, lying on body midline anterior to germarium. Vitellarium dense, often obscuring other reproductive organs and parts of intestinal ceca, coextensive with intestinal ceca, extending to level of anterior margin of haptor; bilateral vitelline ducts far anterior of germarium. Egg deformed in present specimens, with short proximal filament.

**Figures 83–89:**
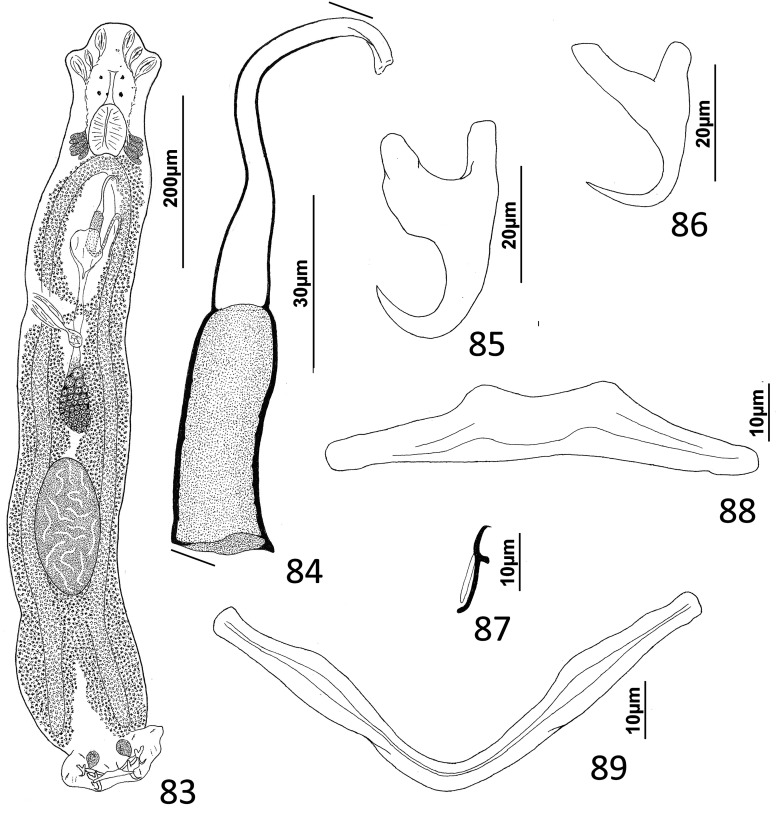
*Lethrinitrema australiense* n. sp. from the spangled emperor, *Lethrinus nebulosus*, Lethrinidae. 83, Whole mount (ventral view, composite); 84, Male copulatory organ (ventral view); 85, Ventral anchor; 86, Dorsal anchor; 87, Hook; 88, Ventral bar; 89, Dorsal bar. Parallel lines on Figure 84 indicate the limits of the dimension measured.

Measurements: Body 691–924 (818; *n* = 11) long; greatest width (excluding haptor) 120–197 (158; *n* = 11). Haptor 106–156 (127; *n* = 10) wide. Ventral anchor 37–41 (39; *n* = 8) long; dorsal anchor 29–35 (33; *n* = 10) long. Ventral bar 60–77 (68; *n* = 9) long; dorsal bar 82–102 (93; *n* = 9) long. Hook 12–15 (13; *n* = 13) long. Pharynx 48–70 (58; *n* = 11) long, 41–54 (47; *n* = 11) wide. Testis 102–158 (132; *n* = 13) long, 59–93 (77; *n* = 13) wide. Male copulatory organ 75–96 (89; *n* = 11) long. Germarium 69–101 (82; *n* = 8) long, 31–51 (42; *n* = 8) wide.

### Remarks

*Lethrinitrema australiense* n. sp. resembles *Lethrinitrema grossecurvitubum* (Li & Chen, 2005) Sun, Li & Yang, 2014 by having the tubular shaft of the MCO in the shape of an interrogation mark. It differs from the latter species by the base of the MCO being nearly as long as the tubular shaft (base much shorter than the shaft of the MCO in *L. grossecurvitubum*) and by having a terminal bulbous expansion of the superficial root of the ventral anchor (absent in *L. grossecurvitubum*) (compare Fig. 85 with figures 2b and 2c in [49] and figures 5E, 5F, and 5G in [82]). Based on comparative morphology of the haptoral and copulatory sclerites, *L. australiense* most closely resembles *Lethrinitrema austrosinense* (Li & Chen, 2005) Sun, Li & Yang, 2104. *Lethrinitrema australiense* and *L. austrosinense* differ by the latter species lacking the interrogation-mark shape of the shaft of the MCO (shaft of MCO slightly arcing in *L. austrosinense*; see figures 1a–c in [49] and figures 6A–H in [82]).

## *Lethrinitrema fleti* (Young, 1968) Lim & Justine, 2011

Syn. *Haliotrema fleti* Young, 1968

(Figs. 90–96)

**Figures 90–96:**
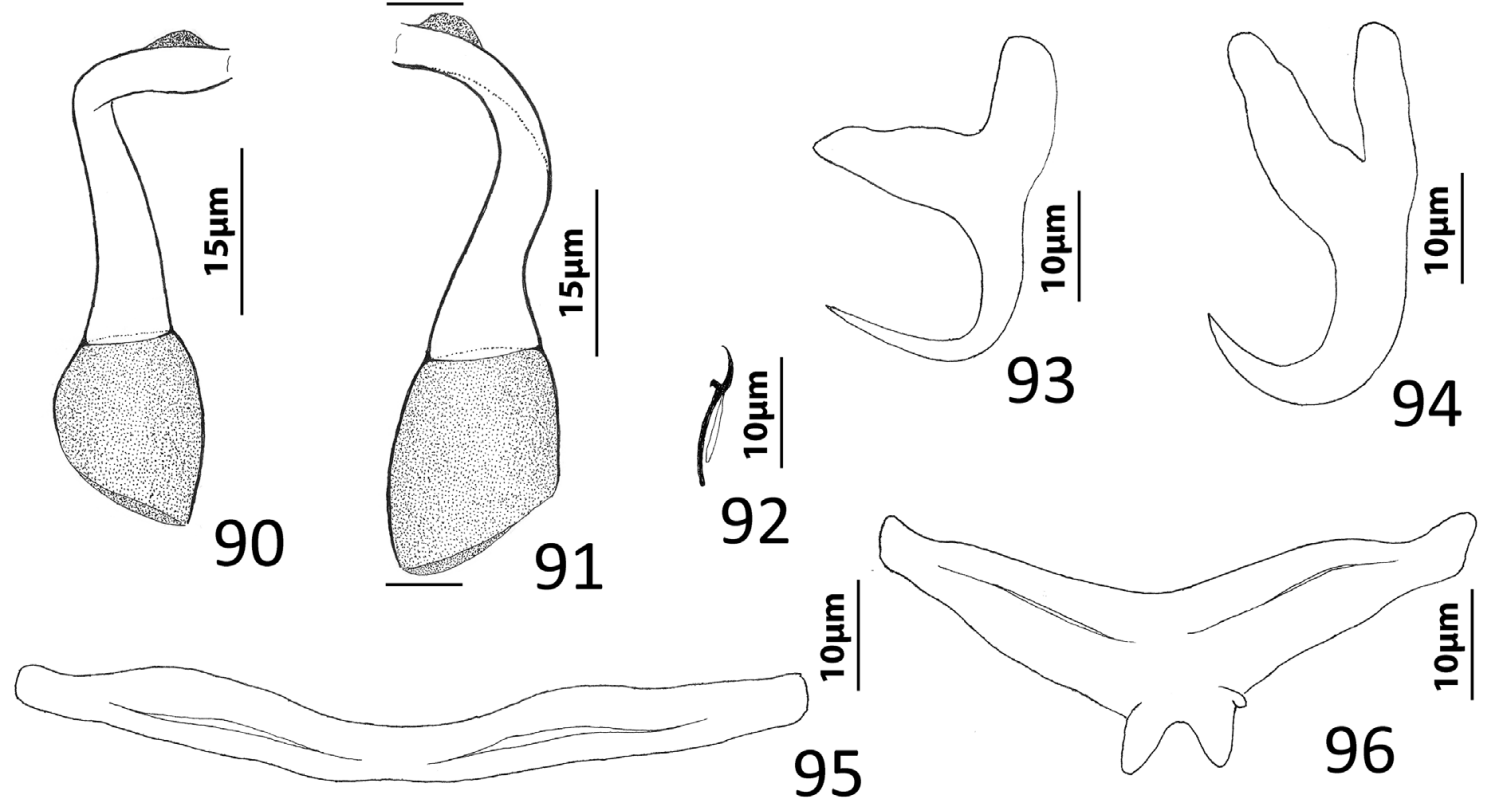
*Lethrinitrema fleti* (Young, 1968) Lim & Justine, 2011 from the spangled emperor, *Lethrinus nebulosus*, Lethrinidae. 90, Male copulatory organ (ventral view); 91, Male copulatory organ (dorsal view); 92, Hook; 93, Dorsal anchor; 94, Ventral anchor; 95, Dorsal bar; 96, Ventral bar. Parallel lines on Figure 91 indicate the limits of the dimension measured.

Type host: Grass emperor, *Lethrinus fletus* Whitley (now *Lethrinus laticaudis* Alleyne & Macleay), Series Eupercaria, Lethrinidae.

Type locality: Moreton Bay, Queensland, Australia.

Current records: *L. laticaudis*: Moreton Bay off Green Island, Queensland, Australia (27°25′ S, 153°14′ E), 11, 12 January 2016; Moreton Bay, Queensland, Australia (specific locality in Moreton Bay not recorded), 12 January 2016. *Lethrinus nebulosus* (Forsskål), Lethrinidae: Moreton Bay off Peel Island, Queensland, Australia (27°30′ S, 153°20′ E), 13 January, 2016.

Previous records: *L. laticaudis* (as *L. fletus*): Moreton Bay, Queensland, Australia [95]. *Lethrinus miniatus* (Forster): Heron Reef off Heron Island, Queensland, Australia [66]; Elford Reef, ~30 mi E of Cairns, Queensland, Australia (16°45′ S, 146°15′ E) [66]; Moreton Bay, Queensland, Australia (as *Lethrinus chrysostomus* Richardson) [95]. *L. nebulosus*: South China Sea [97]; Yangjiang, Guangdong Province, China [88, 89].

*Infection site*: Gill lamellae.

Specimens studied: 26 voucher specimens (from *L. laticaudis*), QM G240880–240884, G240875–240879, USNM 1692894-1692902, HWML 217005, 217006, 217007; voucher specimen (from *L. nebulosus*), HWML 217008.

Measurements (respective measurements from the original description [95] follow in brackets those of current specimens): Body 625–1,470 (905; *n* = 14) [748–1,210] long; greatest width (excluding haptor) 119–198 (164; *n* = 15) [137–175]. Haptor 78–156 (127; *n* = 12) wide. Ventral anchor 33–39 (37; *n* = 6) [31–33] long; dorsal anchor 29–35 (32; *n* = 11) [32–34] long. Ventral bar 46–58 (53; *n* = 9) [44–50] long; dorsal bar 57–71 (65; *n* = 11) [54–60] long. Hook 12–14 (13; *n* = 25) long. Pharynx 51–69 (57; *n* = 15) long, 38–68 (48; *n* = 15) wide. Testis 78–164 (130; *n* = 15) long, 28–100 (71; *n* = 15) wide. Male copulatory organ 46–53 (50; *n* = 10) [34–36] long. Germarium 50–98 (73; *n* = 13) long, 34–52 (43; *n* = 13) wide.

### Remarks

Young’s [95] description of this species as *Haliotrema fleti* Young, 1968 is adequate and corresponded well with the morphological features of present specimens. With the exception of length of the MCO, the respective ranges of the dimensions of present specimens included or overlapped those reported by Young [95] for the species. Young [95] recorded the length of the MCO to be 34–36 μm (5 specimens measured), whereas it was found to be 46–53 μm long (10 specimens measured) among specimens collected during the present study. These differences appear to be fairly significant. However, a comparison of the sizes reported by Young [95] for the haptoral sclerites and those suggested by the 25-μm scalebar applied to his figures 12b to 12f of the MCO and haptoral sclerites clearly indicate that the scale for the figures of the haptoral components is erroneous. Assuming the length of the dorsal anchor depicted in Young’s [95] figure 12d to be 32–34 μm (as he reported in his paragraph of measurements) and applying it as the true scale for his figure 12b of the MCO, suggests that the total length of the MCO was about 41 μm, which closely approaches that reported herein for the MCO of the current specimens. It is apparent, therefore, that his recorded measurement for the length of the MCO as 34–36 μm is also erroneous.

## *Lethrinitrema lituus* n. sp.

urn:lsid:zoobank.org:act:BFBA26DF-322F-4462-AAEC-12CEC1474266

Syns *Lethrinitrema* sp. 1 of Sun *et al.* [82]; *Lethrinitrema* sp. 2 of Sun *et al.* [82].

Type host: Spangled emperor, *Lethrinus nebulosus* (Forsskål), Series Eupercaria, Lethrinidae.

Type locality: Moreton Bay off Peel Island, Queensland, Australia (27°30′ S, 153°20′ E), 13 January 2016.

Previous record: *L. nebulosus*: South China Sea off Zhanjiang, Guangdong Province, China (21°19′ N, 110°40′ E) (as *Lethrinitrema* sp. 1 and *Lethrinitrema* sp. 2) [82].

Infection site: Gill lamellae.

Specimens studied: Holotype, QM G240854; 14 paratypes, QM G240855–240860, USNM 1692877-1692881, HWML 217002; voucher specimen, USNM 1692868.

Etymology: The specific name (a noun) is from Latin (*lituus* = a curved trumpet) and refers to the shape of the male copulatory organ.

### Description (Figs. 97–104)

Body fusiform, flattened dorsoventrally. Cephalic region broad; cephalic lobes moderately developed. Two pairs of poorly defined eyespots, members often dissociated; lenses absent; chromatic granules minute, ovate to subspherical; accessory granules common throughout cephalic region. Pharynx ovate; esophagus short to non-existent; intestinal ceca confluent well posterior to testis. Peduncle broad, tapered posteriorly, with two large gland reservoirs emptying *via* delicate posterior ducts in haptor near anchors. Haptor bilobed, narrow, undifferentiated from peduncle. Ventral anchor robust, with short shaft having proximal angular bend and small hump on deep (dorsal) side, moderately long point ending short of level of tip of superficial root; anchor base with short knob-like deep root, superficial root having terminal cap. Dorsal anchor comparatively delicate, with moderately long superficial root, short deep root, slightly arcing shaft, long point ending short of level of tip of superficial root. Ventral bar rod shaped, enlarged posteromedially; dorsal bar gently bowed to broadly U or rarely broadly V shaped. Hook distribution normal; each hook delicate, with protruding blunt thumb, undilated shank comprised of single subunit; FH loop nearly shank length. Testis subspherical to ovate; proximal portion of vas deferens not observed, distal portion apparently dilating to form fusiform seminal vesicle lying to right of MCO. Prostates, prostatic reservoirs not observed. MCO with inverted-tumbler-shaped base, tubular gently arcing shaft; tip of shaft acute. Germarium elongate, pyriform; oötype, Mehlis’ glands, uterus not observed. Vaginal pore dextromarginal; vagina unsclerotized extending medially to spherical seminal receptacle lying ventral to anterior margin of germarium. Vitellarium dense, often obscuring other reproductive organs, coextensive with intestinal ceca, may extend into peduncle as dendritic arms; bilateral vitelline ducts immediately anterior to germarium. Egg not observed.

**Figures 97–104:**
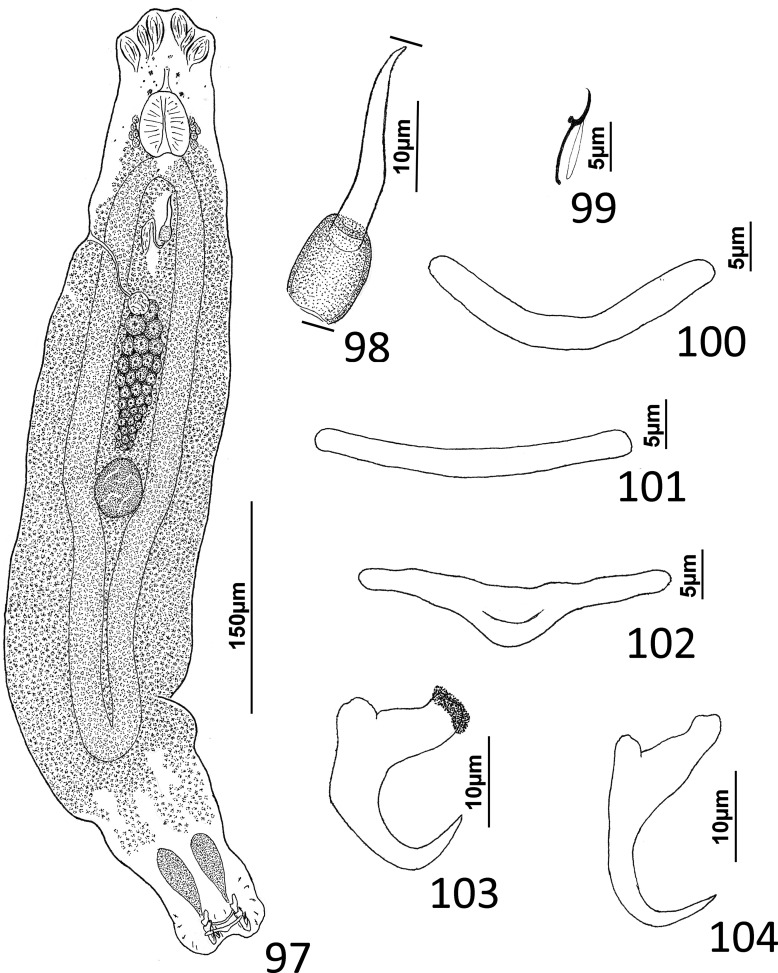
*Lethrinitrema lituus* n. sp. from the spangled emperor, *Lethrinus nebulosus*, Lethrinidae. 97, Whole mount (ventral view, composite); 98, Male copulatory organ (ventral view); 99, Hook; 100, 101, Dorsal bars; 102, Ventral bar; 103, Ventral anchor; 104, Dorsal anchor. Parallel lines on Figure 98 indicate the limits of the dimension measured.

Measurements: Body 520–892 (694; *n* = 11) long; greatest width 115–223 (172; *n* = 13). Haptor 74–92 (82; *n* = 11) wide. Ventral anchor 19–23 (21; *n* = 8) long; dorsal anchor 24–26 (25; *n* = 6) long. Ventral bar 35–44 (40; *n* = 7) long; dorsal bar 33–41 (38; *n* = 7) long. Hook 11–14 (12; *n* = 17) long. Pharynx 47–58 (51; *n* = 5) long, 39–41 (40; *n* = 5) wide. Testis 31–43 (37; *n* = 5) long, 31–38 (35; *n* = 5) wide. Male copulatory organ 31–35 (33; *n* = 4). Germarium 70–116 (93; *n* = 5) long, 42–53 (47; *n* = 5) wide.

### Remarks

Based on only one and four specimens, respectively, Sun *et al.* [82] provided partial descriptions of two unnamed but morphologically similar forms from *Lethrinus nebulosus* in the South China Sea that they assigned to *Lethrinitrema* as sp. 1 and sp. 2. *Lethrinitrema lituus* n. sp. possesses features intermediate between the two forms, but differs from them by 1) having a sigmoid tubular shaft of the MCO (shafts slightly arcing in both *Lethrinitrema* sp. 1 and 2); 2) lacking a superficial groove in the ventral bar (present in *Lethrinitrema* sp. 1); and 3) having a length (31–35μm) of the MCO intermediate between that of *Lethrinitrema* sp. 1 (56 μm long) and *Lethrinitrema* sp. 2 (22–28μm long). Sun *et al.* [82] also used the shapes of the ventral and dorsal bars and differences in the robustness of the superficial roots of the dorsal anchors to differentiate the two undescribed forms, but these differences and those listed above between *L. lituus* and *Lethrinitrema* sp. 1 and sp. 2 apparently represent intraspecific variation and/or varying views due to orientation of structures within the microscopic plane of view; correspondingly, the latter two forms are herein considered synonyms of *L. lituus*.

## *Lethrinitrema nebulosum* Sun, Li, & Yang, 2014

(Figs. 105–110)

**Figures 105–110:**
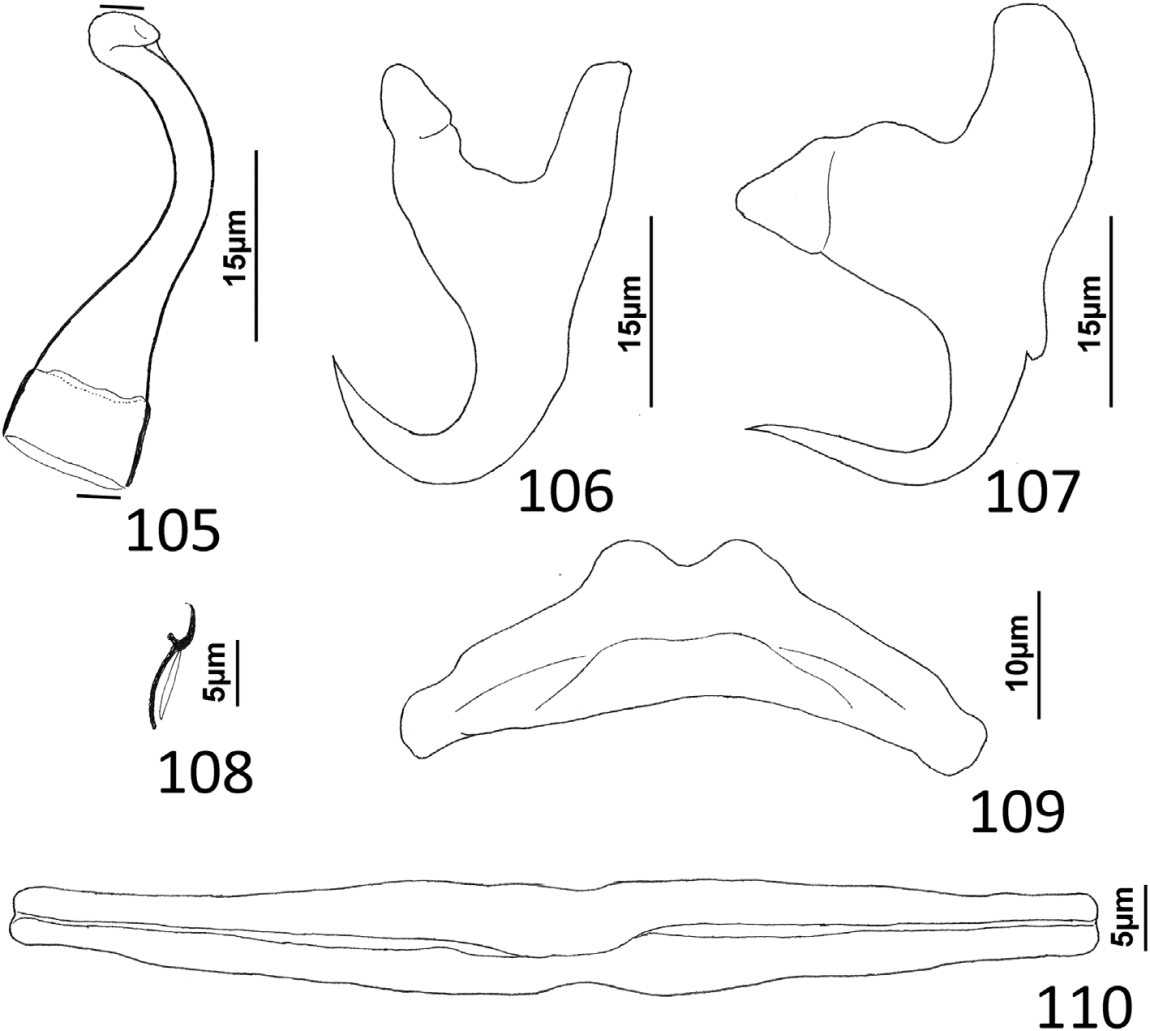
*Lethrinitrema nebulosum* Sun, Li, & Yang, 2014 from the spangled emperor, *Lethrinus nebulosus*, Lethrinidae. 105, Male copulatory organ (ventral view); 106, Ventral anchor; 107, Dorsal anchor; 108, Hook; 109, Ventral bar; 110, Dorsal bar. Parallel lines on Figure 105 indicate the limits of the dimension measured.

Type host: Spangled emperor, *Lethrinus nebulosus* (Forsskål), Series Eupercaria, Lethrinidae.

Type locality: Off Zhanjiang, Guangdong Province, South China Sea (21°19′ N, 110°40′ E).

Current record: *L. nebulosus*: Moreton Bay off Peel Island, Queensland, Australia (27°30′ S, 153°20′ E), 13, 14 January 2016.

Previous record: There have been no additional records of *L. nebulosum* since that of the original description [82].

Infection site: Gill lamellae.

Specimens studied: 9 voucher specimens, QM G240861–240864, USNM 1692882-1692884, HWML 217003.

Measurements (respective measurements from the original description follow in brackets those of current specimens): Body 908–1,140 (1,020; *n* = 5) [538–1,294 (912; *n* = 7)] long; greatest width (excluding haptor) 160–191 (175; *n* = 4) [190–310 (240; *n* = 13)]. Haptor 116–138 (129; *n* = 4) [134–249 (193; *n* = 13)] wide. Ventral anchor 36–37 (*n* = 1) [27–33 (31; *n* = 12)] long; dorsal anchor 39–43 (41; *n* = 3) [30–36 (34; *n* = 13)] long. Ventral bar 43–55 (50; *n* = 6) [38–49 (44; *n* = 13)] long; dorsal bar 77–90 (83; *n* = 6) [62–75 (70; *n* = 13)] long. Hook 12–13 (*n* = 6) [10–12 (11; *n* = 32)] long. Pharynx 52–68 (61; *n* = 5) long, 44–50 (47; *n* = 5) [48–74 (61; *n* = 13)] wide. Testis 56–85 (70; *n* = 2) [51–130 (84; *n* = 12)] long, 48–52 (50; *n* = 2) [39–72 (53; *n* = 12)] wide. Male copulatory organ 37–42 (40; *n* = 3) [22–26 (24; *n* = 12); see Remarks] long. Germarium 95–121 (108; *n* = 2) [45–130 (97; *n* = 13)] long, 45–47 (46; *n* = 2) [36–72 (50; *n* = 13)] wide.

### Remarks

The original description of *L. nebulosum* is adequate [82]. In present specimens, a small nub is present on the deep (ventral) margin of the dorsal anchor, which was not reported in the original description. The species is easily distinguished from all of its congeners parasitizing the spangled emperor by having a slight uplift or posterior recurve of the dorsal anchor point (Fig. 107) (dorsal anchor point is curved anteriorly in all of its congeners). In the original description, an inconsistency was noted between the measurement of the length of the MCO provided in the description (22–26 μm) and that suggested by the scale for figure 3b of the MCO (~ 40 μm). The latter value appears correct based on the respective length determined in present specimens.

The finding of *L. nebulosum* on the spangled emperor in Moreton Bay is a new faunal record for the bay.

## *Ligophorus bostrychus* n. sp.

urn:lsid:zoobank.org:act:D7AFABD3-35EC-4A3F-8AD8-F026DD3654B8

Type host: Greenback mullet, *Planiliza subviridis* (Valenciennes), Mugiliformes, Mugilidae.

Type locality: Moreton Bay off Wynnum, North Stradbroke Island, Queensland, Australia (27°25′ S, 153°11′ E), 18 January 2016.

Infection site: Gill lamellae.

Specimens studied: Holotype, QM G240911; 7 paratypes, QM G240912–240913, USNM 1692930-1692932, HWML 217013.

Etymology: The specific name (a noun) is from Greek (*bostrychos* = a lock of hair) and refers to the distal end of the accessory piece of the copulatory complex.

### Description (Figs. 111–118)

Body proper slender, with nearly parallel lateral margins, slightly flattened dorsoventrally; greatest width usually in anterior trunk. Tegument smooth. Cephalic region broad; one terminal, two bilateral cephalic lobes well differentiated; three pairs of head organs; cephalic glands not observed. Two pairs of eyespots; each eyespot with lens; members of posterior pair usually larger, slightly farther apart than those of anterior pair; chromatic granules ovate to subspherical; accessory granules few or absent in cephalic region. Pharynx subspherical to ovate; esophagus short; intestinal ceca confluent well posterior to gonads. Peduncle broad, slightly tapered toward haptor; haptor subhexagonal, with lateral lobes containing hook pairs 3, 4, 6, 7. Ventral anchor with large base having long superficial root and well-developed deep root, short curved shaft, and long recurved point extending just past level of tip of superficial anchor root; union of shaft and point forming smooth arc. Dorsal anchor delicate compared to ventral anchor, with well-developed basal roots, slightly arcing shaft, short point reaching just short of level of tip of superficial anchor root; union of shaft and point angular. Ventral bar rod shaped, with short truncate median knoll flanked by two delicate anteriorly directed processes; ends of bar rounded, slightly enlarged. Dorsal bar broadly V-shaped, with slightly enlarged ends directed laterally or posterolaterally. Hook distribution normal; hook delicate, with uniform shank, upright acute thumb; FH loop about 3/4 shank length. Genital pore midventral at level of esophagus. MCO with base having flared irregular margin and secondary balloon-like cavity; delicate tubular shaft forming counterclockwise coil of slightly more than one ring. Accessory piece unarticulated with MCO, comprising proximal sheath and distal portion sometimes appearing as a tuft-of-hair. Gonads indistinct; germarium generally pyriform (U-shape reported in some congeners not observed), pretesticular; margins of testis obscured by vitellarium in holotype. Proximal vas deferens not observed; seminal vesicle tear-drop shaped, lying sinistroposterior to base of MCO; elongate prostatic reservoir lying to right of seminal vesicle, may extend posteriorly as far as anterior margin of germarium. Oötype, Mehlis’ gland, uterus not observed. Vaginal pore apparently ventral near body midline immediately anterior to subspherical seminal receptacle; large seminal receptacle, overlying anterior end of germarium. Vitellarium comprising two bilateral bands of vitelline follicles coextensive with intestine; bilateral vitelline ducts not observed. Egg not observed.

**Figures 111–118:**
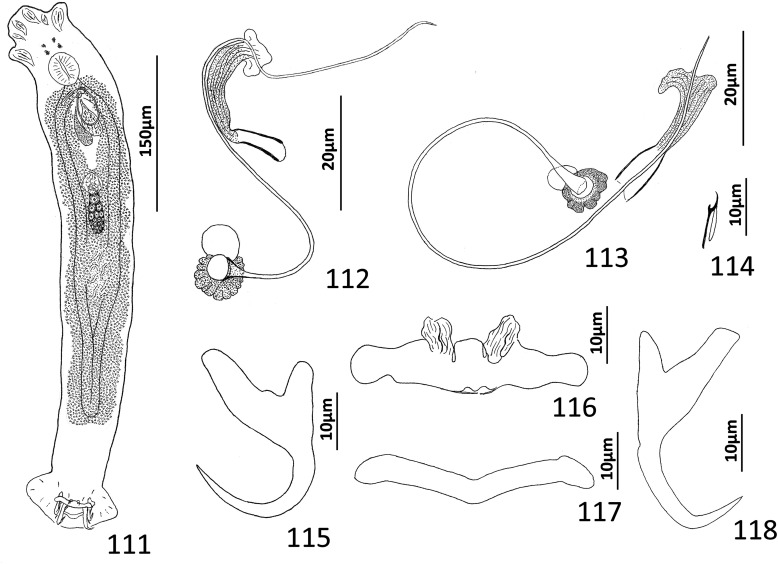
*Ligophorus bostrychus* n. sp. from the greenback mullet, *Planiliza subviridis*, Mugilidae. 111, Whole mount (ventral view, composite); 112, 113, Male copulatory organs (ventral views); 114, Hook; 115, Ventral anchor; 116, Ventral bar; 117, Dorsal bar; 118, Dorsal anchor. Measurements of MCOs (Figs. 112, 113) are the actual lengths obtained using a calibrated curvimeter on drawings drawn with the aid of a camera lucida.

Measurements: Body 468–512 (490; *n* = 2) long; greatest width 83–95 (89; *n* = 2). Haptor 97–102 (100; *n* = 2) wide. Ventral anchor 29–35 (32; *n* = 4) long; dorsal anchor 36–42 (39; *n* = 6) long. Ventral bar 31–45 (38; *n* = 4) long; dorsal bar 35–44 (39; *n* = 5) long. Hook 11–14 (12; *n* = 14) long. Pharynx 32–34 (33; *n* = 2) long, 23–26 (25; *n* = 2) wide. Testis 27–28 (*n* = 1) long, 27–28 (*n* = 1) wide. Male copulatory organ (actual length) 104–119 (111; *n* = 5); accessory piece 24–35 (27; *n* = 5) long. Germarium 29–59 (44; *n* = 2) long, 26–30 (28; *n* = 2) wide.

### Remarks

Species of *Ligophorus* Euzet & Suriano, 1977 are often difficult to identify and differentiate because of their nearly identical internal anatomy, with species determination often depending on seemingly small morphological differences in the haptoral and copulatory sclerites. Adding to the difficulty of identification is that some species of mullets, the only group of fishes known to serve as hosts for these helminths, are infected by multiple *Ligophorus* spp. For example, the cosmopolitan *Mugil cephalus* Linnaeus hosts at least 15 species of *Ligophorus*; *Valamugil buchanani* Bleeker [valid as *Crenimugil buchanani* (Bleeker) and *Planiliza subviridis* with at least eight species each (not including *L. bostrychus* n. sp. from *P. subviridis*); and *Chelon saliens* (Risso) and *Planiliza carinata* (Valenciennes) with as many as five and six species, respectively. Finally, the apparently low-level host specificity of some *Ligophorus* spp. further hinders identification (see [40, 76, 79, 80, 81].

**Figures 119–125:**
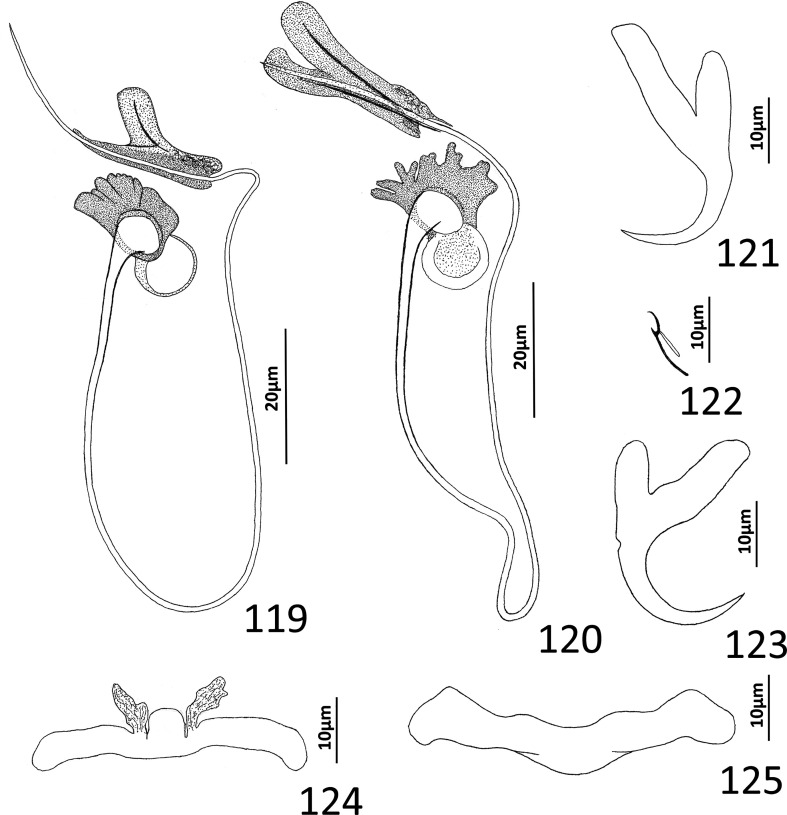
*Ligophorus kaohsianghsieni* (Gussev, 1962) Gussev, 1985 from the greenback mullet, *Planiliza subviridis*, Mugilidae. 119, 120, Copulatory complexes (ventral views). 121, Dorsal anchor; 122, Hook; 123, Ventral anchor; 124, Ventral bar; 125, Dorsal bar. Measurements of MCOs (Figs. 119, 120) are the actual lengths obtained using a calibrated curvimeter on drawings made using a camera lucida.

*Ligophorus bostrychus* n. sp. is distinguished from all other congeners by having 1) an accessory piece comprised of a proximal sheath and a distal portion often appearing to form a tuft-of-hair; 2) a ventral anchor with an evenly recurved short shaft and elongate point; and 3) a dorsal anchor slightly larger than the ventral anchor and having an angular union of the shaft and point. It most closely resembles *Ligophorus kaohsianghsieni* (Gussev, 1962) Gussev, 1985, by having a smoothly arcing shaft and point of the ventral anchor, elongate filiform MCO, and similar haptoral bars. *Ligophorus bostrychus* is easily distinguished from *L. kaohsianghsieni* in the comparative morphology of the accessory piece of the copulatory complex, by having dorsal and ventral anchors of noticeably different sizes and by having a shorter MCO (compare Figs. 112–118 and Figs. 119–125; also see [18, 76]).

## *Ligophorus kaohsianghsieni* (Gussev, 1962) Gussev, 1985

Syns *Ancyrocephalus kaohsianghsieni* Gussev, 1962; *Ligophorus kaohsinghsieni* of Dmitrieva (1996) (lapsus)

(Figs. 119–125)

Type host: So-iuy mullet, *Mugil soiuy* Basilewsky [now *Planiliza haematocheilus* (Temminck & Schlegel)], Mugiliformes, Mugilidae.

Type locality: Tumen and Liao Rivers of northeastern China (see Remarks).

Current record: *Planiliza subviridis*: Moreton Bay off Wynnum, North Stradbroke Island, Queensland, Australia (27°25′ S, 153°11′ E), 18 January 2016.

Previous records: See [12, 76, 86].

Infection site: Gill lamellae.

Specimens studied: 8 voucher specimens, QM G240914–240916, USNM 1692927-1692929, HWML 217012.

Measurements (dimensions of the sclerites were not provided by Gussev [17]; respective measurements from Gussev [18] follow in brackets those of current specimens): Body 667–733 (700; *n* = 2) [1,500] long; greatest width (excluding haptor) 144–168 (156; *n* = 2) [400]. Haptor 117–139 (128; *n* = 2) [71–208 (150; *n* = 60)] wide. Ventral anchor 25–32 (29; *n* = 5) [37–40] long; dorsal anchor 34–40 (36; *n* = 5) [39–41] long. Ventral bar 34–42 (38; *n* = 5) [45] long; dorsal bar 40–48 (43; *n* = 6) [43] long. Hook 12–14 (13; *n* = 14) [15] long. Pharynx 55–59 (57; *n* = 2) [46–59 (53; *n* = 30)] long, 46–50 (48; *n* = 2) [46–59 (53; *n* = 30)] wide. Testis 90–97 (94; *n* = 2) long, 47–54 (50; *n* = 2) wide. Male copulatory organ (actual length) 187–208 (199; *n* = 6) [250–265]; accessory piece 21–28 (25; *n* = 6) [60–75] long. Germarium 55–60 (58; *n* = 2) long, 25–44 (35; *n* = 2) wide.

### Remarks

The redescription of *L. kaohsianghsieni* by Sarabeev *et al.* [76] is sufficient for defining the species. The presence of *L. kaohsianghsieni* on greenback mullets in Moreton Bay (two of three mullets infected, prevalence 67%) represents a new host record for the helminth and a new faunal record for the bay. The species has been previously reported from China, Russia, Ukraine, and Bulgaria, and previously reported hosts included *P. haematocheilus*, *Liza aurata* (Risso) [now *Chelon auratus* (Risso)], and *Mugil cephalus* Linnaeus. The helminth occurs naturally within the waters of the western Pacific Ocean and was introduced along with its type host into eastern Europe from the Sea of Japan [76, 86].

Gussev’s [17] statement indicating the hosts of *Ancyrocephalus vanbenedenii* (Parona & Perugia, 1890) Johnston & Tiegs, 1922 [now *Ligophorus vanbenedenii* (Parona & Perugia, 1890) Euzet & Suriano, 1977] and *A. kaohsianghsieni* Gussev, 1962 [now *Ligophorus kaohsianghsieni* (Gussev, 1962) Gussev, 1985] is unclear, when he (page 341 in the Russian text) simply listed the two dactylogyrids followed by a list of hosts identified only by the common Russian names Лoбaнa, кeфaлeй (the loban, *Mugil cephalus*) and Пилeнгaca (the pilengas, *M. soiuy*). Apparently using the same parasite specimens that were available in 1962, Gussev ([18], page 211]) clarified the statement when he indicated that *A. kaohsianghsieni* had been found only on the pilengas, *M. soiuy*.

Gussev [17], the author of *L. kaohsianghsieni*, did not identify the type locality nor any other localities from which his specimens of the species were collected, although he later [18] listed the Tumen-Ula and Liaohe rivers of northeastern China as localities in his redescription of the species that was apparently based on the collecting localities of the same specimens he had before him in 1962. The formal description of *Ancyrocephalus kaohsianghsieni* Gussev, 1962 was to have been provided in a paper by Gussev *et al.* (Gussev, A. V., Zhukov, E. V., & Shulman S. S. 1962. Parasites of fish of the Liaohe River. Parazitologicheskii Sbornik), which was listed in the Literature Cited in Gussev [17] but never published (E. Dmitrieva, pers. comm.). Based on its title, the unpublished paper of Gussev *et al.* would likely have established the type locality for the helminth as the Liaohe River of northeastern China, which Sarabeev *et al.* [76] recognized as such for *L. kaohsianghsieni*. However, Gussev [18] listed both the Tumen-Ula and the Liaohe rivers in his list of localities for the species, whereas Soo & Lim [79] gave the Sea of Japan as its type locality. Article 73.2.3 of the ICZN states that “if the syntypes originated from two or more localities…, the type locality encompasses all of the places of origin (of the specimens) (parentheses ours).” Thus, the type locality for *L. kaohsianghsieni* includes both the Liaohe and Tumen-Ula rivers of northeastern China, which were listed by Gussev [18] and apparently based on specimens used for the original description of the species. It’s native range currently includes the two Chinese rivers (as type locality), the Sea of Japan, the East China Sea, and the South China Sea (see [86]), and now eastern Australia [*ex nobis*].

## *Ligophorus parvicopulatrix* Soo & Lim, 2012

(Figs. 126–132)

**Figures 126–132:**
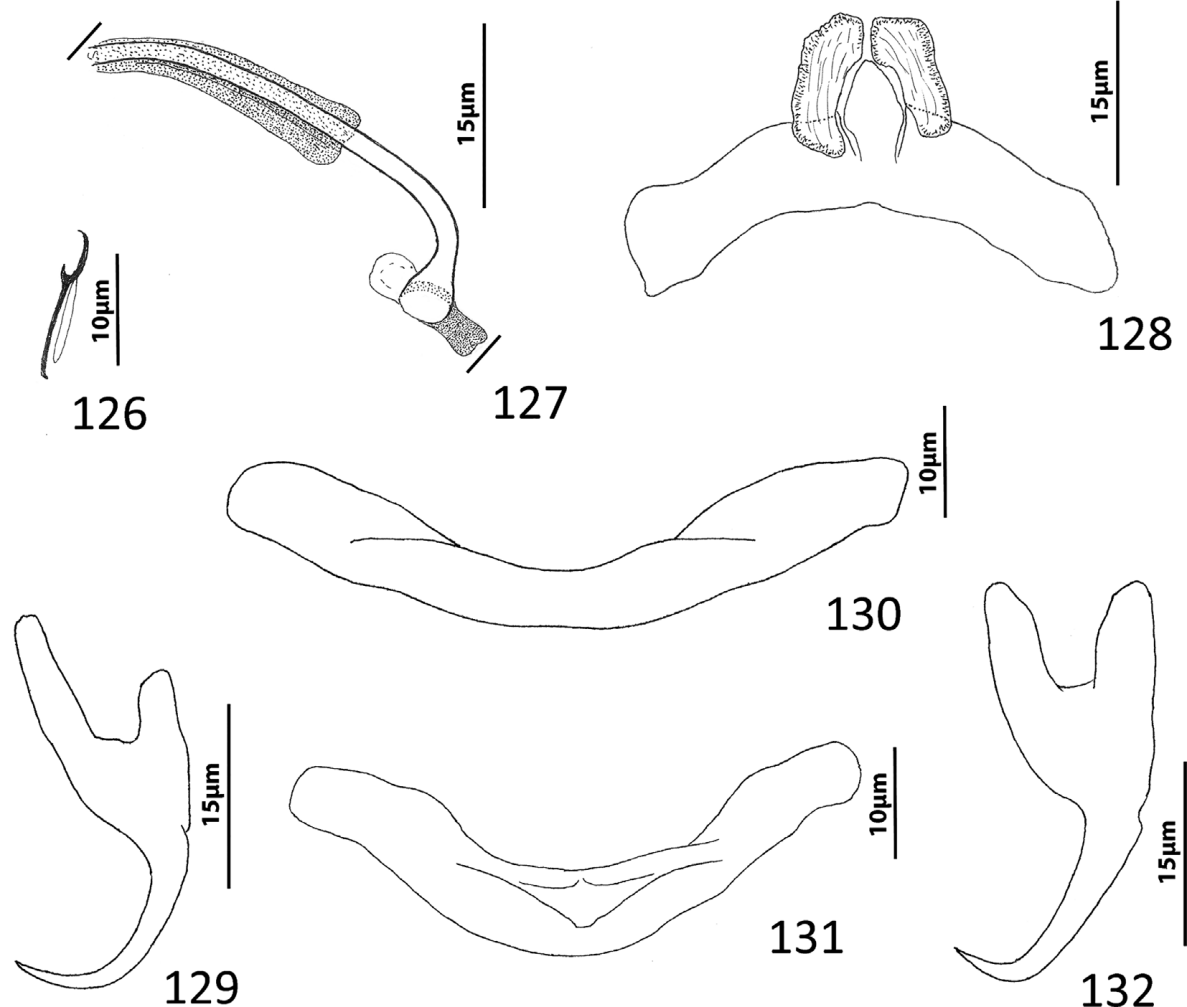
*Ligophorus parvicopulatrix* Soo & Lim, 2012 from the greenback mullet, *Planiliza subviridis*, Mugilidae. 126, Hook; 127, Copulatory complex (ventral view); 128, Ventral bar; 129, Dorsal anchor; 130, 131, Dorsal bars; 132, Ventral anchor. Parallel lines on Figure 127 indicate the limits of the dimension measured.

Type host: Greenback mullet, *Liza subviridis* (Valenciennes) [now *Planiliza subviridis* (Valenciennes)], Mugiliformes, Mugilidae.

Type locality: Off Carey Island, Banting, Malaysia (02°51′ N, 101°22′ E).

Current record: *P. subviridis*: Moreton Bay off Wynnum, North Stradbroke Island, Queensland, Australia (27°25′ S, 153°11′ E), 18 January 2016.

Previous record: There have been no additional records for *L. parvicopulatrix* since the original description [79].

Infection site: Gill lamellae.

Specimens studied: 36 voucher specimens, QM G240895–240910, USNM 1692915-1692926, HWML 217011.

Measurements (respective measurements from the original description [79] follow in brackets those of current specimens): Body 664–922 (815; *n* = 10) [642–1,454 (1,078; *n* = 60)] long; greatest width (excluding haptor) 112–188 (143; *n* = 11) [73–231 (166; *n* = 60)]. Haptor 117–154 (134; *n* = 8) [71–208 (150; *n* = 60)] wide. Ventral anchor 34–41 (37; *n* = 16) [32–36 (34; *n* = 60)] long; dorsal anchor 28–34 (31; *n* = 21) [23–30 (27; *n* = 60)] long. Ventral bar 38–45 (42; *n* = 22) [36–46 (39; *n* = 60)] long; dorsal bar 45–60 (53; *n* = 21) [38–59 (49; *n* = 60)] long. Hook 12–14 (13; *n* = 28) [9–13 (11; *n* = 60)] long. Pharynx 42–50 (47; *n* = 12) [46–59 (53; *n* = 30)] long, 35–46 (40; *n* = 12) [46–59 (53; *n* = 30)] wide. Testis 73–132 (106; *n* = 11) long, 33–76 (56; *n* = 11) wide. Male copulatory organ 33–42 (37; *n* = 10) [41–71 (48; *n* = 60)] long. Germarium 49–83 (62; *n* = 10) long, 29–59 (44; *n* = 10) wide.

### Remarks

*Ligophorus parvicopulatrix*, along with *Ligophorus kaohsianghsieni* and *Ligophorus bostrychus* n. sp., occurred concomitantly on the gill lamellae of the greenback mullet in Moreton Bay; *L. parvicopulatrix* had a prevalence of 67% (two of three greenback mullets infected) and the highest intensity of the three species co-occurring on the fish. The present specimens of *L. parvicopulatrix* corresponded closely with the original description, except that the haptoral hooks possessed an upright acute thumb (upright thumb shown to be blunt in figure 10E in Soo & Lim [79]) and the superficial roots of the dorsal anchors in present specimens were comparatively longer than that shown in figure 10A of Soo & Lim [79]; the respective measurements of current specimens overlapped or fell within the respective ranges reported by Soo & Lim [79].

The occurrence of *L. parvicopulatrix* in Moreton Bay represents a new faunal record for the bay.

## *Neohaliotrema gemmula* n. sp.

urn:lsid:zoobank.org:act:C7BC76A1-FEA3-40CA-AD50-2414D8D4F72C

Type host: Indo-Pacific sergeant, *Abudefduf vaigiensis* (Quoy & Gaimard), Series Ovalentaria, Pomacentridae.

Type locality: Moreton Bay off Amity Point, North Stradbroke Island, Queensland, Australia (27°23′ S, 153°26′ E), 11–19 January 2016.

Infection site: Gill lamellae.

Specimens studied: Holotype, QM G240941; 4 paratypes, QM G240942–240943, USNM 1692955-1692956.

Etymology: The specific name (a noun) is from Latin (*gemmula* = a small gem).

### Description (Figs. 133–142)

Body proper fusiform, slightly flattened dorsoventrally; greatest width usually near midlength at level of germarium. Tegument smooth. Cephalic region broad; two terminal, two bilateral cephalic lobes poorly to moderately differentiated; three bilateral pairs of head organs; bilateral pair of cephalic glands posterolateral to pharynx. Single pair of eyespots; each eyespot with lens; chromatic granules small, ovate; accessory granules few in cephalic region. Pharynx subspherical; esophagus short; intestinal ceca confluent posterior to testis. Peduncle short, tapering slightly toward haptor; haptor with prominent bilateral lobes containing hook pairs 3, 4 and 7. Ventral anchor with base having elongate superficial root and shorter deep root, shaft distally expanded forming small angular internal blade, elongate point with slight distal recurve; sharp angular union of point and shaft. Dorsal anchor robust, with well-developed basal roots (superficial root longer than deep root, with small rose-thorn-like spur at tip), robust shaft having large internal blade, delicate doubly recurved point. Ventral and dorsal bars similar in shape; each with posteromedial shield-like expansion, blunt slightly expanded ends. Hook distribution normal; hook of respective pairs with delicate shaft and point, upright acute thumb, shank differing in length and robustness among hook pairs and composed of two poorly differentiated subunits. Shanks of hook pair 1 & 6 elongate, uniform in diameter; proximal subunit of shank of hook 2 robust, directed posteriorly in haptor from its origin, giving rise to recurved distal subunit; hook shanks of pairs 3–5 and 7 short; FH loop extending to near union of shank subunits of all hook pairs. Genital pore medioventral near level of esophageal bifurcation. MCO lightly sclerotized, comprising bulbous base and J-shaped tubular shaft. Accessory piece variable, unarticulated with MCO, serving as guide for distal portion of shaft of MCO. Gonads intercecal, subovate, tandem; testis postgermarial. Vas deferens not observed; seminal vesicle lying sinistrally at level of seminal receptacle, a simple dilation of distal vas deferens; ejaculatory duct elongate, extending from seminal vesicle to base of MCO; prostates not observed; single prostatic reservoir forming inverted U or J dorsal to MCO. Oötype surrounded by two large bilateral masses of Mehlis’ glands; uterus conspicuous, dilated, extending along body midline to genital pore. Vaginal pore, vagina not observed; small seminal receptacle lying immediately anterior to Mehlis’ gland. Vitellarium dense, coextensive with intestinal ceca; bilateral vitelline ducts at level of seminal receptacle. Egg not observed.

**Figure 133:**
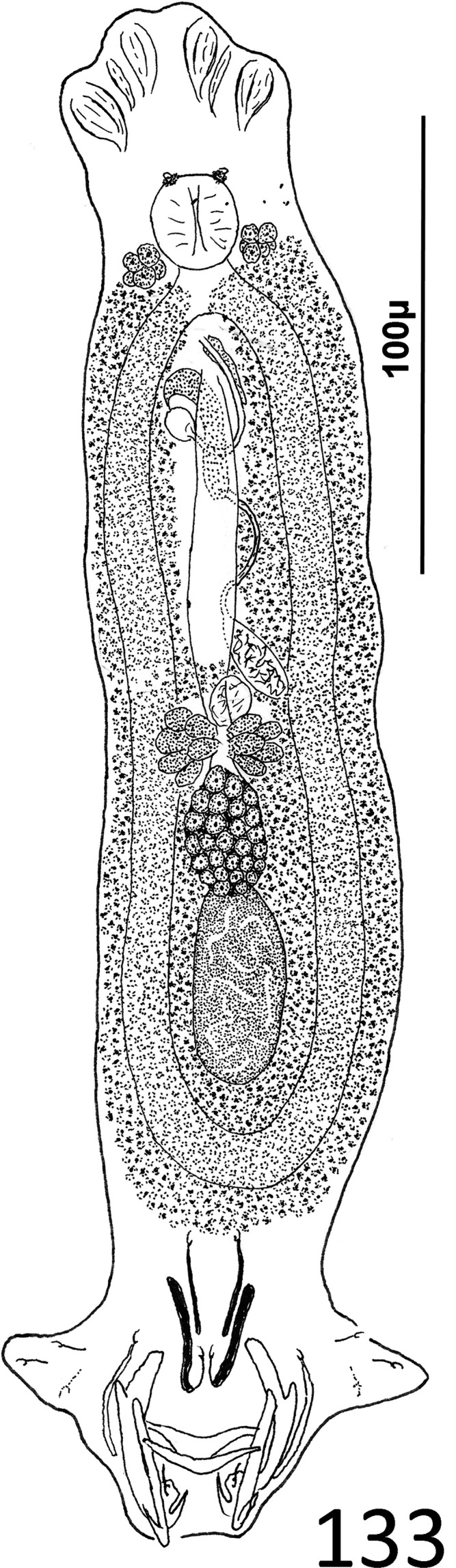
Whole mount (ventral view, composite) of *Neohaliotrema gemmula* n. sp. from Indo-Pacific sergeant, *Abudefduf vaigiensis*, Pomacentridae.

**Figures 134–142:**
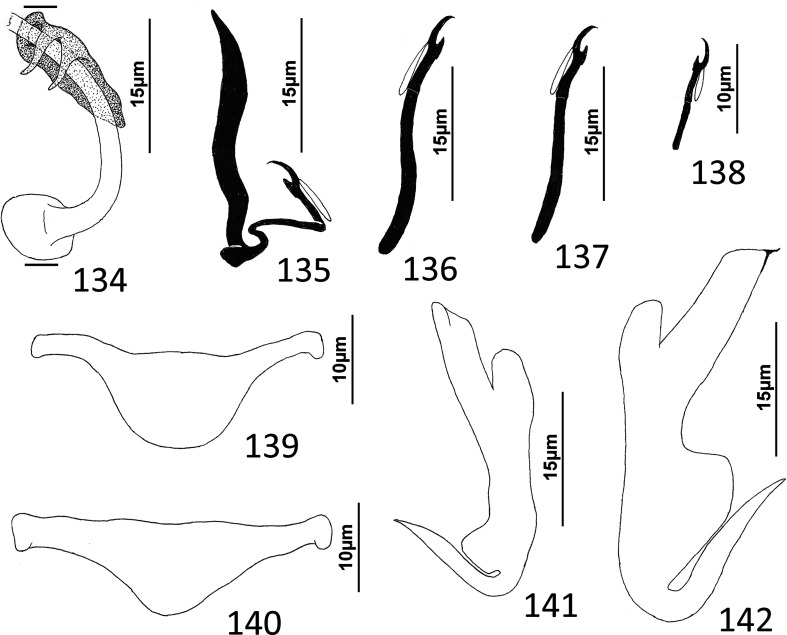
Haptoral and copulatory sclerites of *Neohaliotrema gemmula* n. sp. 134, Copulatory complex (ventral view); 135, Hook (pair 2); 136, Hook (pair 6); 137, Hook (pair 1); 138, Hook (pairs 3–5, 7); 139, Dorsal bar; 140, Ventral bar; 141, Ventral anchor; 142, Dorsal anchor. Parallel lines on Figure 134 indicate the limits of the dimension measured.

Measurements: Body 348–356 (352; *n* = 2) long; greatest width 70–72 (71; *n* = 2). Haptor 92–100 (96; *n* = 2) wide. Ventral anchor 33–44 (38; *n* = 3) long; dorsal anchor 44–48 (46; *n* = 3) long. Ventral bar 37–38 (*n* = 1) long; dorsal bar 34–39 (36; *n* = 2) long. Hook pair 1, 27–30 (28; *n* = 3) long; hook pairs 3–5, 7, 11–14 (12; *n* = 8) long; hook pair 6, 28–29 (*n* = 2) long; a measurement for the length of hook pair 2 could not be obtained because of its variable shape. Pharynx 19–28 (23; *n* = 2) wide. Testis 69–88 (79; *n* = 2) long, 22–68 (45; *n* = 2) wide. Male copulatory organ 29–32 (31; *n* = 2) long. Germarium 40–77 (59; *n* = 2) long, 20–43 (32; *n* = 2) wide.

### Remarks

This species most closely resembles *Neohaliotrema moretonense* n. sp., from which it is easily distinguished by having 1) a small blade on the distal internal surface of the shaft of the ventral anchor (distal internal surface of shaft of the ventral anchor with deep notch and lacking a blade in *N. moretonense*); 2) a ventral anchor point with its tip slightly recurved posteriorly (ventral anchor point straight in *N. moretonense*); 3) a comparatively shorter hook pair 7; and 4) ventral and dorsal haptoral bars with prominent posteromedial shield-like processes (posteromedial processes minimally developed in *N. moretonense*).

## *Neohaliotrema malayense* Lim & Gibson, 2010

(Figs. 143–150)

**Figures 143–150:**
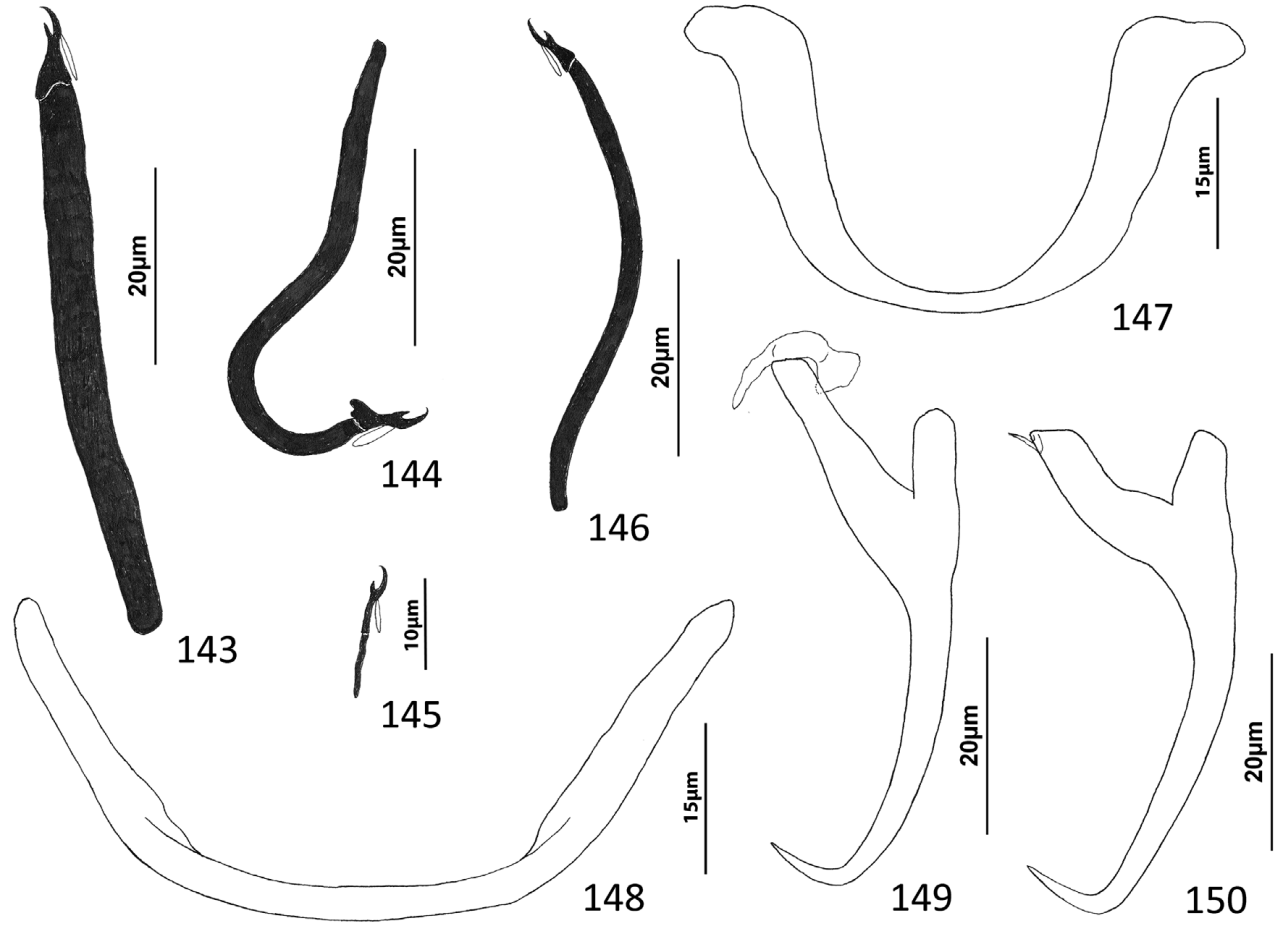
*Neohaliotrema malayense* Lim & Gibson, 2010 from the Indo-Pacific sergeant, *Abudefduf vaigiensis*, Pomacentridae. 143, Hook (pair 1); 144, Hook (pair 2); 145, Hook (pairs 3–5, 7); 146, Hook (pair 6); 147, Dorsal bar; 148, Ventral bar; 149, Dorsal anchor; 150, Ventral anchor.

Type host: Indo-Pacific sergeant, *Abudefduf vaigiensis* (Quoy & Gaimard) (as *A. vaigensis*, a misspelling), Series Ovalentaria, Pomacentridae.

Type locality: Andaman Sea off Pulau Langkawi, Malaysia.

Current records: *A. vaigiensis*: Moreton Bay off Amity Point, North Stradbroke Island, Queensland, Australia (27°23′ S, 153°26′ E), 11–18 January 2016. *Abudefduf bengalensis* (Bloch): Moreton Bay off Amity Point, North Stradbroke Island, Queensland, Australia (27°23′ S, 153°26′ E), 11–18 January 2016. *Abudefduf whitleyi* Allen & Robertson: Moreton Bay off Amity Point, North Stradbroke Island, Queensland, Australia (27°23′ S, 153°26′ E), 11–18 January 2016.

Previous record: There have been no additional records of *A. malayense* since the original description [53].

Infection site: Gill lamellae.

Specimens studied: 16 voucher specimens (from *A. vaigiensis*), QM G240917–240921, USNM 1692933–1692939, HWML 217015; 6 voucher specimens (from *A. bengalensis*), QM G240922–240923, USNM 1692940–1692941, HWML 217016; 5 voucher specimens (from *A. whitleyi*), QM G240924–240925, USNM 1692942–1692043, HWML 217014.

Measurements: Table 3.

**Table 3:** Measurements (in micrometers) of *Neohaliotrema malayense* parasitizing the gill lamellae of three pomacentrids off Australia and Malaysia.

Host	*Abudefduf vaigiensis*	*Abudefduf bengalensis*	*Abudefduf bengalensis*	*Abudefduf whitleyi*
Locality	(Moreton Bay, Australia)	(Malaysia)	(Moreton Bay, Australia)	(Moreton Bay, Australia)
Reference	[*ex nobis*]	[53]	[*ex nobis*]	[*ex nobis*]
Body				
Length	357–423 (406; *n* = 7)	444–597 (510; *n* = 30)	385–423 (406; *n* = 3)	370–415 (396; *n* = 3)
Greatest width	53–72 (64; *n* = 7)	56–100 (85; *n* = 30)	58–69 (65; *n* = 3)	55–82 (66; *n* = 3)
Haptor				
Width	131–172 (149; *n* = 7)	128–208 (176; *n* = 30)	152–168 (158; *n* = 3)	137–152 (144; *n* = 3)
Pharynx				
Length	20–25 (23; *n* = 6)	20–31 (25; *n* = 30)	22–30 (26; *n* = 3)	22–26 (24; *n* = 2)
Width	20–25 (22; *n* = 6)	19–31 (25; *n* = 30)	21–29 (25; *n* = 3)	22–25 (24; *n* = 2)
Testis				
Length	51–131 (79; *n* = 6)	–	59–76 (66; *n* = 3)	68–81 (74; *n* = 2)
Width	24–35 (31; *n* = 6)	–	27–31 (29; *n* = 3)	27–31 (29; *n* = 2)
Germarium				
Length	36–47 (40; *n* = 4)	–	38–61 (50; *n* = 3)	33–46 (40; *n* = 2)
Width	20–24 (22; *n* = 4)	–	22–26 (23; *n* = 3)	24–26 (25; *n* = 2)
Ventral anchor				
Length	47–54 (50; *n* = 9)	44–50 (47; *n* = 60)	49–53 (51; *n* = 3)	48–52 (50; *n* = 2)
Dorsal anchor				
Length	53–58 (55; *n* = 9)	46–53 (51; *n* = 60)	54–59 (56; *n* = 3)	52–57 (54; *n* = 2)
Ventral bar				
Length	68–88 (75; *n* = 8)	62–81 (72; *n* = 30)	72–75 (73; *n* = 2)	68–69 (*n* = 1)
Dorsal bar				
Length	57–90 (67; *n* = 8)	71–82 (74; *n* = 28)	58–61 (59; *n* = 2)	63–64 (*n* = 1)
Hook pair 1				
Length	50–66 (61; *n* = 9)	55–65 (60; *n* = 60)	62–67 (64; *n* = 3)	56–62 (59; *n* = 2)
Hook pairs 3–5, 7				
Length	14–17 (15; *n* = 15)	10–16 (13; *n* = 236)	13–16 (14; *n* = 5)	13–15 (14; *n* = 4)
Hook pair 6				
Length	42–63 (56; *n* = 7)	49–62 (56; *n* = 60)	34–52 (43; *n* = 2)	52–59 (55; *n* = 2)

### Remarks

The comparative morphology and the respective measurements of the sclerotized components of the haptor of the specimens from Moreton Bay corresponded closely with those of *N. malayense* reported by Lim & Gibson [53]. However, the copulatory complexes of the Australian specimens were all obscured by the dense vitellarium, and as a result could not be compared with Lim & Gibson’s [53] description of the species. Nonetheless, similarity of the haptoral sclerites leaves little doubt of the identification of the Australian specimens as being *N. malayense*.

*Abudefduf whitleyi and A. bengalensis* represented new host records for *N. malayense*, and the occurrence of the helminth in Moreton Bay constituted a new faunal record for the bay.

## *Neohaliotrema moretonense* n. sp.

urn:lsid:zoobank.org:act:E91728CC-2128-4E4A-8931-F9F9B8FE82C7

Syn. *Neohaliotrema* sp. of Lim & Gibson [53]

Type host: Indo-Pacific sergeant, *Abudefduf vaigiensis* (Quoy & Gaimard), Series Ovalentaria, Pomacentridae.

Type locality: Moreton Bay off Amity Point, North Stradbroke Island, Queensland, Australia (27°23′ S, 153°26′ E), 11–19 January 2016.

Other record: *Abudefduf bengalensis* (Bloch): Moreton Bay off Amity Point, North Stradbroke Island, Queensland, Australia (27°23′ S, 153°26′ E), 11–18 January 2016.

Previous record: *A. vaigiensis*: Andaman Sea off Pulau Langkawi, Malaysia (as *Neohaliotrema* sp.) [53].

Infection site: Gill lamellae.

Specimens studied: Holotype, QM G240926; 20 paratypes (from *A. vaigiensis*), QM G240927–240934, USNM 1692944–1692950, HWML 217017; 13 voucher specimens (from *A. bengalensis*), QM G240935–240940, USNM 1692951–1692954, HWML 217018.

Etymology: The specific name reflects Moreton Bay off Brisbane, Australia, containing the type locality from which the species was collected.

### Description (Figs. 151–160)

Body proper fusiform, slightly flattened dorsoventrally; greatest width usually near midlength at level of germarium. Tegument smooth. Cephalic region broad; two terminal, two bilateral cephalic lobes poorly to moderately differentiated; three pairs of head organs; bilateral pair of cephalic glands posterolateral to pharynx. Single pair of eyespots; each eyespot with lens often obscured by chromatic eye granules; chromatic granules small, ovate; accessory granules few in cephalic region. Pharynx spherical; esophagus short; intestinal ceca confluent posterior to testis. Peduncle tapering toward haptor; haptor with prominent bilateral lobes containing hook pairs 3, 4, 7. Ventral anchor with base having elongate superficial root and shorter deep root, short robust terminally truncate shaft with distal indentation at union of shaft and point, straight elongate point; sharp angular union of point and shaft. Dorsal anchor robust, with well-developed basal roots, shaft distally expanded to form large internal blade, and delicate doubly recurved point; rounded union of shaft and point; superficial roots of dorsal and ventral anchors with fingernail-like tips. Haptoral bars minimally enlarged posteromedially; ventral bar broadly V shaped, with tapered semi-acute ends; dorsal bar broadly U or V shaped, with truncate ends directed laterally. Hook distribution normal; hook of respective pairs with delicate shaft and point, upright acute thumb, shank with differing lengths among hook pairs; shank composed of two poorly differentiated subunits; FH loop extending to near union of shank subunits. Shanks of hook pairs 1, 6 elongate, uniform in diameter; proximal subunit of shank of hook 2 robust, directed posteriorly in haptor from its origin, giving rise to delicate recurved distal subunit; hook shanks of pairs 3–5, 7 short. Genital pore on body midline, ventral to esophagus. MCO lightly sclerotized, with bulbous base and delicate J-shaped tubular shaft. Accessory piece unarticulated with MCO, morphologically variable depending on orientation, often frayed distally, serving as guide for distal portion of shaft of MCO. Gonads intercecal, subovate, tandem; testis postgermarial. Vas deferens, seminal vesicle, prostatic reservoir not observed. Oötype surrounded by two bilateral masses of Mehlis’ glands; uterus conspicuous, dilated, extending along body midline toward genital pore. Vaginal pore, vagina, seminal receptacle not observed. Vitellarium dense, coextensive with intestinal ceca; bilateral vitelline ducts partially overlain by anterior portion of Mehlis’ gland. Egg not observed.

**Figures 151–160:**
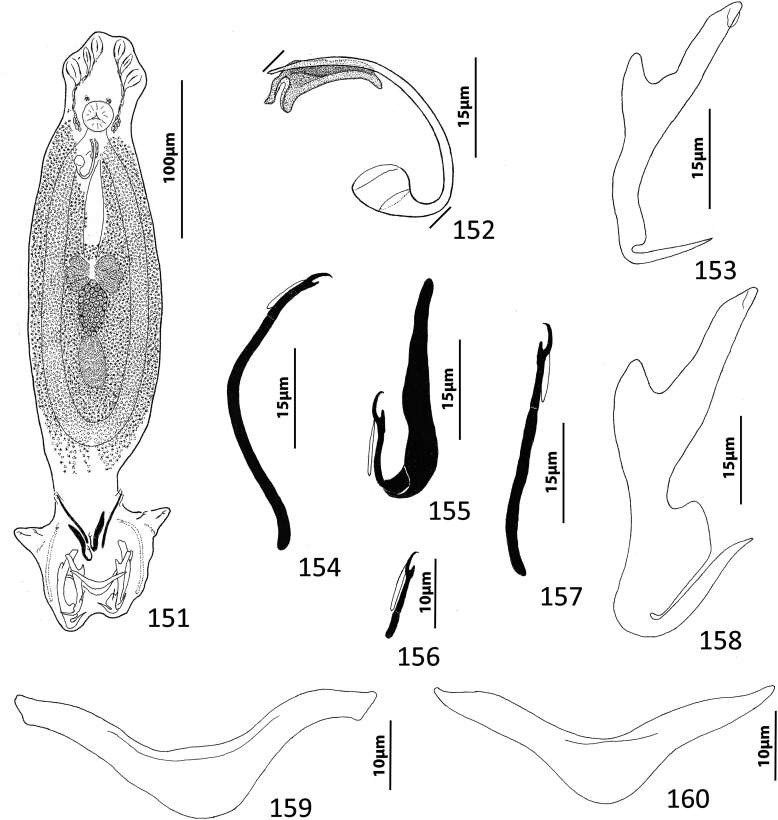
*Neohaliotrema moretonense* n. sp. from the Indo-Pacific sergeant, *Abudefduf vaigiensis*, Pomacentridae. 151, Whole mount (ventral view, composite); 152, Copulatory complex (ventral view); 153, Ventral anchor; 154, Hook (pair 6); 155, Hook (pair 2); 156, Hook (pairs 3–5, 7); 157, Hook (pair 1); 158, Dorsal anchor; 159, Dorsal bar; 160, Ventral bar. Parallel lines on Figure 152 indicate the limits of the dimension measured.

Measurements (measurements of specimens collected from *A. bengalensis* follow in brackets those collected from *A. vaigiensis*, respectively): Body 369–395 (381; *n* = 3) [328–347 (337; *n* = 3)] long; greatest width (excluding haptor) 69–92 (79; *n* = 5) [69–79 (75; *n* = 5)]. Haptor 93–104 (98; *n* = 5) [87–97 (93; *n* = 3)] wide. Ventral anchor 40–43 (41; *n* = 16) [40–45 (43; *n* = 7)] long; dorsal anchor 49–57 (53; *n* = 16) [49–58 (54; *n* = 8)] long. Ventral bar 41–51 (46; *n* = 13) [44–53 (49; *n* = 2)] long; dorsal bar 45–51 (47; *n* = 13) [42–51 (45; *n* = 6)] long. Hook pair 1, 37–40 (38; *n* = 12) [37–47 (41; *n* = 7)] long; pairs 3–5 & 7, 13–15 (14; *n* = 17) [12–14 (13; *n* = 12)] long; pair 6, 44–52 (48; *n* = 16) [(43–51 (48; *n* = 6)] long; a measurement for the length of hook pair 2 could not be obtained because of its variable shape, but see scale for Fig. 155. Pharynx 21–25 (23; *n* = 3) [20–25 (22; *n* = 4)] long, 20–23 (21; *n* = 3) [19–24 (22; *n* = 4)] wide. Testis 37–47 (43; *n* = 4) [27–45 (35; *n* = 3)] long, 21–31 (25; *n* = 5) [20–24 (22; *n* = 23] wide. Male copulatory organ 32–39 (36; *n* = 8) [33–34 (*n* = 1)] long. Germarium 25–32 (29; *n* = 4) [41–51 (44; *n* = 5)] long, 21–30 (25; *n* = 4) [21–30 (27; *n* = 5)] wide.

### Remarks

Lim & Gibson [53] first reported this species as *Neohaliotrema* sp. from the gills of *Abudefduf vaigiensis* from Malaysia. They stated that their specimens resembled *Neohaliotrema macracanthum*, which was described by Zhukov [98] from the gills of the *Abudefduf saxatilis* (Linnaeus) and *Abudefduf taurus* (Müller & Troschel) in the Gulf of Mexico off Havana, Cuba. Mendoza-Franco *et al.* [56], who recorded supplementary observations on specimens of *N. macracanthum* parasitizing *A. saxatilis* from the Cayo Arcas Reef in the Gulf of Mexico, failed to provide illustrations of the species, although their reported measurements of the body size and haptoral and copulatory sclerites compared with those presented by Lim & Gibson [53]. Lim & Gibson [53] indicated that their specimens were consistently larger than those reported by Zhukov [98] and identified some morphological differences in the ventral bar and the hooks of pair 2, which suggested that *N. macracanthum* and *Neohaliotrema* sp. may not be conspecific. Although not mentioned by Lim & Gibson [53], the ventral anchor shafts of *Neohaliotrema* sp. form a nearly uniform arc (Fig. 153), whereas those of *N. macracanthum* were clearly shown to have an obvious distal double curvature (see figure 1*a* in Zhukov [98]). These differences along with *N. macracanthum* and *Neohaliotrema* sp. parasitizing different host species that are restricted to the waters of the Atlantic and Pacific oceans, respectively, would further support non-conspecificity. Thus, *N. moretonense* is proposed for the specimens from Moreton Bay and for those of *Neohaliotrema* sp. of Lim and Gibson [53].

Li *et al.* [48] described *Neohaliotrema tukerhamatus* Li, Chang, & Wu, 2010, *Neohaliotrema antiacanthus* Li, Chang, & Wu, 2010, and *Neohaliotrema bengalense* Li, Chang, & Wu, 2010 (the latter as *N. bengalensis*) from three species of *Abudefduf*, two of which were identified as *A. vaigiensis* and *A. bengalensis*, from the South China Sea off Guangdong, China. The drawings provided by Li *et al.* [48] of the haptoral and copulatory sclerites of the three helminths are similar albeit overly diagrammatic, suggesting that the validity of some or all of the three species remains questionable. However, if the drawings of the dorsal haptoral bars of the Chinese forms reasonably reflect the actual morphology of the bars (see figures 2, 10, and 15 of Li *et al.* [48]), the group may represent a valid species of *Neohaliotrema*. Dorsal bars, having a broad width and posterolaterally directed rounded ends as shown in these figures, do not occur in any of the remaining described congeners, including *N. moretonense* n. sp. and *N. gemmula* n. sp. Nonetheless, a re-examination of the *Neohaliotrema* species infesting *Abudefduf* spp. in the South China Sea will be necessary to determine the validity of the species described by Li *et al.* [48].

## *Pleuronectitrema* n. gen.

urn:lsid:zoobank.org:act:7B7871A4-58E5-428D-8414-2BAF8CCC287B

Type species: *Pleuronectitrema spirula* n. sp. from the large toothed flounder, *Pseudorhombus arsius* (Hamilton), Pleuronectiformes, Paralichthyidae*.*

Other species: *Pleuronectitrema arsiosa* (Venkatanarasaiah, 1984) n. comb.; *Pleuronectitrema kuwaitense* n. sp.; *Pleuronectitrema youngi* (Venkatanarasaiah, 1984) n. comb. (all from *P. arsius*); *Pleuronectitrema* sp. (syn. *Haliotrema* sp. of Zhang [97]).

Etymology: The generic name reflects the order of flatfishes, the Pleuronectiformes, species of which harbored members of the new genus.

### Diagnosis

Body fusiform, slightly flattened dorsoventrally, comprising body proper (cephalic region, trunk, peduncle) and haptor. Tegument smooth. Two terminal, two bilateral cephalic lobes; three pairs of bilateral head organs; bilateral cephalic glands unicellular, lateral or posterolateral to pharynx. Eyespots present. Mouth subterminal, midventral, prepharyngeal; pharynx a muscular bulb; esophagus short; intestinal ceca two, confluent posterior to gonads, lacking diverticula. Common genital pore midventral near level of intestinal bifurcation. Gonads intercecal, tandem; germarium pretesticular. Testis entire; vas deferens apparently looping left intestinal cecum; seminal vesicle a simple dilation of distal portion of vas deferens. Copulatory complex lacking accessory piece. MCO tubular, coiled, with bulbous or funnel-shaped base; coil with counterclockwise rings. Germarium entire, uterus extending anteriorly from germarium along body midline. Vaginal pore midventral (possibly also dextroventral) anterior to germarium; seminal receptacle pregermarial. Vitellarium in trunk, absent from regions of other reproductive organs. Haptor armed with dorsal and ventral anchor/bar complexes, seven pairs of similar hooks with normal distribution. Ventral and dorsal anchor/bar complexes similar in shape. Hooks with normal dactylogyrid distribution; each hook having upright acute thumb, shank comprised of single subunit. Parasites of pleuronectiform fishes.

### Remarks

*Pleuronectitrema* gen. n. includes species that are morphologically similar to those of *Euryhaliotrema*, species of which share the following features with those of *Pleuronectitrema*: 1) haptoral hooks with upright and acute thumbs and shanks comprised of a single subunit, 2) dorsal and ventral anchor/bar complexes, 3) tandem gonads (testis postgermarial), and 4) a coiled MCO with counterclockwise rings [30]. *Pleuronectitrema* differs from *Euryhaliotrema* by its species having a ventral vaginal pore (vaginal pore dextromarginal or submarginal in species of *Euryhaliotrema*) and by lacking an accessory piece in the copulatory complex (most species of *Euryhaliotrema* possess an accessory piece that may or may not be articulated to the MCO). Species of *Pleuronectitrema* are parasitic on the gills of flatfishes (Pleuronectiformes), whereas all known species of *Euryhaliotrema* occur on fishes formerly or currently assigned to the Perciformes. Although ecological characters such as host preferences are generally weak for differentiating taxa, the finding of similar dactylogyrid species sharing a basically identical internal anatomy that differs from that of species assigned to *Euryhaliotrema* and *Haliotrema* and parasitizing hosts assigned to different orders of fishes would appear to provide additional support for the new genus.

Venkatanarasaiah [84] described *Haliotrema arsiosa* Venkatanarasaiah, 1984 and *Haliotrema youngi* Venkatanarasaiah, 1984 found parasitizing *Pseudorhombus arsius* in India. The comparative morphology of the haptoral and copulatory sclerites of these species and that of the type species of *Pleuronectitrema* suggest that the two Indian species are phylogenetically close to *P*. *spirula* (compare Figs. 162–167 with figures 1–12 in Venkatanarasaiah [84]). Venkatanarasaiah [84] reported that both Indian species possessed a dextral vaginal pore, but did not provide a whole-mount drawing of the helminth nor specifically identify the position of the pore relative to the body margin or whether it was located on the dorsal or ventral surface of the body. If the pores of the two Indian species are dextroventral, the most likely option, their positions would not exclude the Indian species from *Pleuronectitrema*. Thus, *H. arsiosa* and *H. youngi* are transferred to *Pleuronectitrema* as *Pleuronectitrema arsiosa* (Venkatanarasaiah, 1984) n. comb. and *Pleuronectitrema youngi* Venkatanarasaiah, 1984) n. comb., respectively.

**Figures 161–167:**
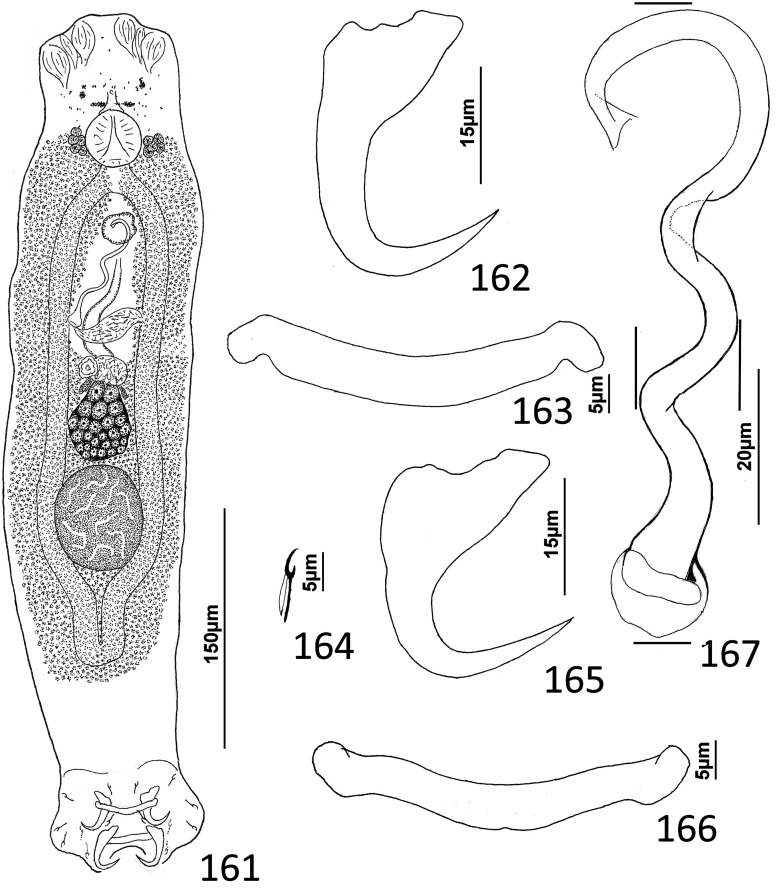
*Pleuronectitrema spirula* n. sp. from the large toothed flounder, *Pseudorhombus arsius*, Paralichthyidae. 161, Whole mount (ventral view, composite); 162, Dorsal anchor; 163, Dorsal bar; 164, Hook; 165, Ventral anchor; 166, Ventral bar; 167, Male copulatory organ (ventral view). Parallel lines on Figure 167 indicate the limits of the dimensions measured.

Zhang [97] reported *Haliotrema* sp. from the five-eyed flounder, *Pseudorhombus quinquocellatus* Weber & de Beaufort, from Haikou, Hainan Province, China (20°00′ N, 110°03′ E). Although he [97] did not provide information on the internal anatomy of the species, he did provide a brief description and drawings of the haptoral and copulatory sclerites, which suggest that the species represents another species of *Pleuronectitrema*, to which it is here transferred as *Pleuronectitrema* sp. It resembles *P. arsiosa*, and *P. youngi* in the comparative morphology of the MCO, but is distinguished from these species by having an elongate filamentous structure (apparently the accessory piece) associated with the MCO. This species requires naming and a full description to meet the requirements of the ICZN [22], which likely will depend on a new collection of specimens from the host.

In a section of the Results, following that dealing with the dactylogyrids from Moreton Bay, *Pleuronectitrema kuwaitense* n. sp. from the gills *P. arsius* collected off Kuwait is described. Although not occurring in Moreton Bay, this species is morphologically similar to *P. ariosa* and *P. youngi*, has a midventral vaginal pore, and is described below to provide added support for the new genus. *Pleuronectitrema kuwaitense* is differentiated below from its congeners in the Remarks following its description.

Species infecting pleuronectiform fishes have been previously assigned to two other monotypic dactylogyrid genera: *Protancyrocephalus* Bychowsky, 1957 with *Protancyrocephalus strelkowi* Bychowsky, 1957 from *Limanda aspera* (Pallas), taken off the eastern coast of Russia; and *Protancyrocephaloides* Burn, 1978 with *Protancyrocephaloides liopsettae* Burn, 1957, from *Liopsetta putnami* (Gill), off the eastern coast of the United States. *Pleuronectitrema* n. gen. differs from these genera by its species possessing ventral vaginal pores and two anchor/bar complexes in the haptor (in species of both *Protancyrocephalus* and *Protancyrocephaloides*, the vaginal pores are dextromarginal; both dorsal and ventral haptoral bars are lacking in *Protancyrocephalus streldowi*; and the dorsal bar is absent in *Protancyrocephaloides lipsettae* [2, 3, 91].

## *Pleuronectitrema spirula* n. sp.

urn:lsid:zoobank.org:act:CFE99D7E-D072-4A8E-AC28-ED006B6DE111

Type host: Large toothed flounder, *Pseudorhombus arsius* (Hamilton), Pleuronectiformes, Paralichthyidae.

Type locality: Moreton Bay off Green Island, Queensland, Australia (27°25′ S, 153°14′ E), 12, 13 January 2016.

Infection site: Gill lamellae.

Specimens studied: Holotype, QM G240885; 21 paratypes, QM G240886–240894, USNM 1692903–1692909, HWML 217009.

Etymology: The specific name (a noun) is from Latin (*spirula* = a small spiral) and refers to the male copulatory organ.

### Description (Figs. 161–167)

Body flattened dorsoventrally, with nearly parallel lateral margins. Cephalic region broad; cephalic lobes moderately to poorly developed. Two pairs of eyespots; members often dissociated, poorly defined, and lacking lenses; chromatic granules minute, ovate to subspherical; accessory granules common throughout cephalic region. Pharynx subspherical to ovate. Peduncle broad, slightly tapered posteriorly. Haptor subhexagonal in outline. Ventral and dorsal anchors similar in shape; each anchor robust, with large base having short superficial root and minimally developed deep root, evenly curved shaft and point; superficial root of dorsal anchor shorter than that of ventral anchor. Ventral and dorsal bars gently arced; ends of ventral bar directed anterolaterally, those of dorsal bar directed posterolaterally. Hooks delicate; FH loop nearly shank length. Testis subspherical to ovate; proximal portion of vas deferens not observed, distal portion dilating to form fusiform seminal vesicle; seminal vesicle extending diagonally from left side of trunk to base of MCO. Prostates, prostatic reservoirs not observed. MCO a loosely coiled tubular shaft arising from small bulbous base; shaft with distal loop and slightly flared termination; coil of MCO counterclockwise, with about 2½ rings. Germarium pyriform; oötype not observed; small Mehlis’ glands lying along anterolateral margins of germarium. Uterus delicate, slightly dilated. Vaginal pore midventral, ventral to seminal receptacle; vaginal canal not observed, apparently short. Seminal receptacle variable in shape, lying immediately anterior to germarium. Vitellarium dense, coextensive with intestinal ceca; bilateral vitelline ducts extend toward body midline where they fuse dorsal to seminal receptacle. Egg not observed.

Measurements: Body 338–774 (579; *n* = 14) long; greatest width (excluding haptor) 84–154 (130; *n* = 15). Haptor 79–117 (102; *n* = 11) wide. Ventral anchor 25–33 (30; *n* = 6) long; dorsal anchor 33–39 (37; *n* = 6) long. Ventral bar 44–55 (48; *n* = 6) long; dorsal bar 42–50 (47; *n* = 6) long. Hook 10–12 (11; *n* = 17) long. Pharyngeal diameter 33–46 (40; *n* = 16). Testis 47–108 (71; *n* = 12) long, 40–69 (57; *n* = 12) wide. Male copulatory organ 65–86 (79; *n* = 6) long; ring diameter 12–16 (14; *n* = 6). Germarium 50–81 (64; *n* = 11) long, 39–57 (47; *n* = 11) wide.

### Remarks

*Pleuronectitrema spirula* n. sp. is the type species of the genus and differs from its congeners by having a loosely coiled MCO with about 2½ counterclockwise rings (MCOs of *P. ariosa*, *P. youngi*, and *P. kuwaitense* with less than 2½ rings) and by having dorsal and ventral anchors with short, minimally developed basal roots (dorsal and ventral anchors of *P. ariosa* and *P. youngi* with comparatively elongate superficial roots; superficial root elongate in the dorsal anchor of *P. kuwaitense*) (compare Figs. 162–166 with Figs. 181–187 and figures 1–12 in Venkatanarasaiah [84]).

## *Tetrancistrum sigani* Goto & Kikuchi, 1917

Syns *Tetrancistrum nebulosi* Young, 1967; *Pseudohaliotrematoides granulosum* Yao, Wang, Xia, & Chen, 1998; *Pseudohaliotrematoides* sp. of Ko and Chan [29].

Type host: Mottled spinefoot, *Siganus fuscescens* (Houttuyn), Acanthuriformes, Siganidae.

Type locality: Japan.

Current records: *S. fuscescens*: Moreton Bay off Green Island, Queensland, Australia (27°25′ S, 153°14′ E), 11, 13 January 2016; Moreton Bay off Peel Island, Queensland, Australia (27°30′ S, 153°20′ E), 14 January 2016.

Previous records: *S. fuscescens*: Great Barrier Reef off Heron Island, Queensland, Australia (23°27′ S, 151°55′ E), 15–22 July 2001 [38]; Gulf of Tonkin (South China Sea) near Lingao, Hainan Province, China (23°27′ S, 151°55′ E), 11 July 2004, 15 January 2006 [38]; Tarumi, Hyôgo Prefecture, Japan (Yamaguti [90]; this record was overlooked by Kritsky *et al.* [38]). *Epinephelus chlorostigma* (Valenciennes), Epinephelidae: Japan (Ishii & Sawada [24]; if not an accidental infection, this fish as a natural host for *T. sigani* requires confirmation). See additional records for *T. sigani* in Kritsky *et al.* [38].

Infection site: Gill lamellae.

Specimens studied: 3 voucher specimens, QM G240945–240946 (from Green Island), QM G240947 (from Peel Island).

Measurements: Body 1,130–1,350 (1,240; *n* = 2) long; greatest width 306–318 (312; *n* = 2). Haptor 102–119 (111; *n* = 2) wide. Ventral anchor 78–79 (*n* = 1) long; dorsal anchor 76–93 (83; *n* = 3) long. Ventral bar 24–25 (*n* = 1) long; dorsal bar 31–32 (*n* = 1) long. Pharynx 63–71 (67; *n* = 2) long, 59–63 (61; *n* = 2) wide. Testis 147–157 (152; *n* = 2) long, 140–146 (143; *n* = 2) wide. Male copulatory organ 73–74 (*n* = 1) long; accessory piece 80–81 (*n* = 1) long. Germarium 86–118 (102; *n* = 2) long, 89–100 (94; *n* = 2) wide.

### Remarks

Only three specimens of *T. sigani* were found parasitizing the mottled spinefoot in Moreton Bay. These specimens corresponded closely with the redescription of the species by Kritsky *et al.* [38], who provided drawings sufficient for identification of the species. The species was previously reported from *S. fuscescens* in Moreton Bay by Young [94].

## *Tetrancistrum siganioides* n. sp.

urn:lsid:zoobank.org:act:40452DE8-D4A7-41CE-A630-A39F571EE1B3

Type host: Mottled spinefoot, *Siganus fuscescens* (Houttuyn), Acanthuriformes, Siganidae.

Type locality: Moreton Bay off Green Island, Queensland, Australia (27°25′ S, 153°14′ E), 11, 13 January 2016.

Other records: *S. fuscescens*: Moreton Bay off Peel Island, Queensland, Australia (27°30′ S, 153°20′ E), 14 January 2016; Moreton Bay off Garden Island, Queensland, Australia (27°37′ S, 153°20′ E), 24 June 2016; Moreton Bay off Dunwich, North Stradbroke Island, Queensland, Australia (27°29′ S, 153°23′ E), 18 January 2016.

Infection site: Gill lamellae.

Specimens studied: Holotype, QM G240944; 14 paratypes, QM G240948–240953, USNM 1692957-1692961, HWML 217019.

Etymology: The specific name (an adjective) refers to the similarity of the species to *Tetrancistrum sigani*.

### Description (Figs. 168–173)

With characters of the genus as emended by Kritsky *et al.* [38]. Body foliiform, flattened dorsoventrally; cephalic region tapered anteriorly from trunk, with moderately to poorly developed bilateral and terminal cephalic lobes; three bilateral pairs of head organs; paired bilateral groups of prepharyngeal and post pharyngeal cephalic glands present. Eyespots absent; chromatic granules minute, ovate to subspherical, scattered throughout cephalic region. Mouth subterminal, midventral, at level of head organs; pharynx elongate ovate; esophagus short, bifurcating to form two intestinal ceca; intestinal ceca lacking diverticula, terminating blindly posterior to testis. Peduncle broad, tapered posteriorly; haptor poorly differentiated from peduncle. Ventral anchor robust, with short shaft and point forming even arc at their union, broad base having large grooved roots, superficial root longer than deep root. Dorsal anchor with robust grooved deep root, short rod-like superficial root, straight shaft, short point. Bars similar, lightly sclerotized; each rod shaped, with expanded terminations. Hooks absent (in adults). Testis subspherical to ovate; vas deferens, seminal vesicle, prostatic vesicles not observed. Copulatory complex comprising unarticulated MCO and accessory piece. MCO an arcing tubular shaft with variably flared distal end; base of MCO with subrectangular posterior and variable anterior flanges. Accessory piece an arcing often flattened rod with proximal flange and small distal terminal knob. Germarium cone shaped, forming cap over anterior end of testis; oötype, Mehlis’ glands not observed; uterus delicate extending anteriorly along body midline to genital pore. Vaginal pore dextromarginal; vagina unsclerotized, with distal thick-walled vestibule; vaginal canal delicate, extending to small seminal receptacle lying near body midline anterior to germarium. Vitellarium dense, coextensive with intestinal ceca; bilateral vitelline ducts immediately anterior to germarium. Egg not observed.

**Figures 168–173:**
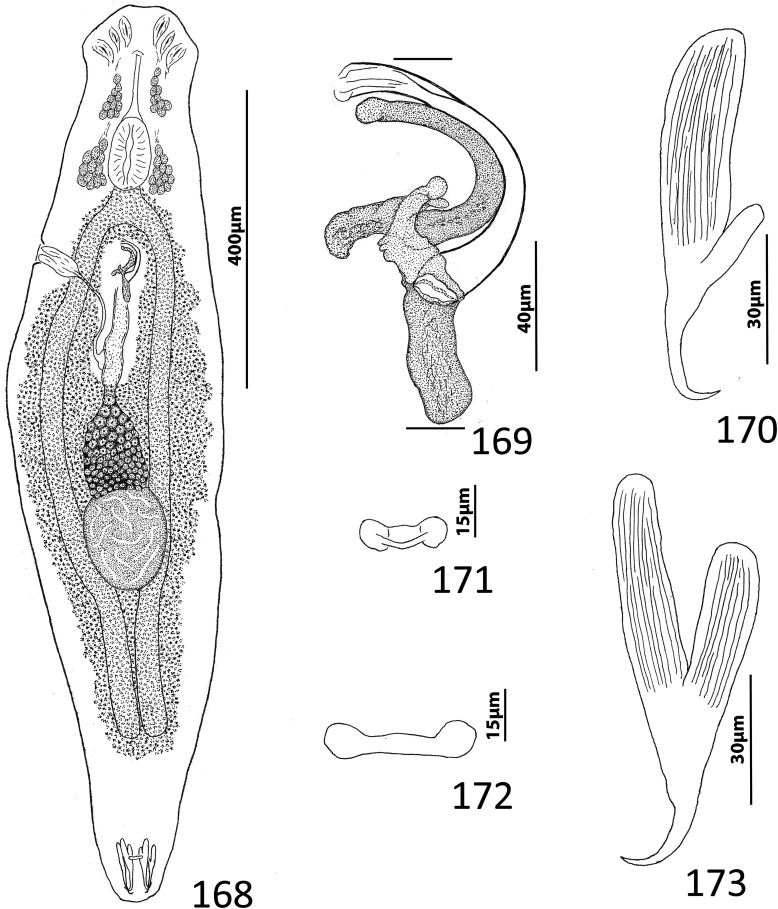
*Tetrancistrum siganioides* n. sp. from the mottled spinefoot, *Siganus flavescens*, Siganidae. 168, Whole mount (ventral view, composite); 169, Copulatory complex (ventral view); 170, Dorsal anchor; 171, Ventral bar; 172, Dorsal bar; 173, Ventral anchor. Parallel lines on Figure 169 indicate the limits of the dimension measured.

Measurements: Body 877–1,260 (1,160; *n* = 8) long; greatest width 272–421 (344; *n* = 8). Haptor 88–138 (112; *n* = 6) wide. Ventral anchor 90–101 (94; *n* = 6) long; dorsal anchor 78–100 (87; *n* = 6) long. Ventral bar 27–28 (*n* = 5) long; dorsal bar 30–38 (35; *n* = 6) long. Pharynx 60–93 (75; *n* = 8) long, 51–63 (56; *n* = 8) wide. Testis 132–200 (171; *n* = 8) long, 133–190 (158; *n* = 8) wide. Male copulatory organ 74–119 (97; *n* = 10) long. Germarium 76–135 (117; *n* = 5) long, 100–147 (127; *n* = 5) wide.

### Remarks

Including *Tetrancistrum siganioides*, *Tetrancistrum* currently includes 17 valid species, 11 of which occur on species of *Siganus* (Siganidae), five on *Naso* spp. (Acanthuridae), and one on a *Lutjanus* sp. (Lutjanidae) [1, 38]. *Tetrancistrum sigani* and *T. siganioides* n. sp. are the only congeners currently known to parasitize the mottled spinefoot, with *T. sigani* being the most morphologically similar congener of *T. siganioides*. *Tetrancistrum siganioides* is easily differentiated from *T. sigani* by lacking diverticula of the intestinal ceca (lateral diverticula of the intestinal ceca present in *T. sigani*), by having an unarticulated MCO and accessory piece of the copulatory complex (MCO and accessory piece basally articulated in *T. sigani*), by the shaft of the MCO forming a smooth arc and with a flared distal tip (shaft of MCO J shaped and having an unflared distal end with a diagonal opening in *T. sigani*), and by having a small knob-like termination of the accessory piece (termination of accessory piece large and club-like in *T. sigani*).

## *Triacanthinella falcanalis* (Young, 1968) Lim & Gibson, 2008

Syn. *Haliotrema falcanalis* Young, 1968

Type host: Black-flag tripodfish, *Triacanthus falcanalis* Ogilby (now *Tripodichthys angustifrons* (Hollard), Tetraodontiformes, Triacanthidae.

Type locality: Moreton Bay, Queensland, Australia.

Current record: *T. angustifrons*: Moreton Bay off Green Island, Queensland, Australia, 12–14 January 2016.

Previous records: There are no other records of *T. falcanalis* other than that of Young [95] for the original description of the species.

Infection site: Gill lamellae.

Specimens studied: 38 voucher specimens, QM G241000–241014, USNM 1692999–1693011, HWML 217027.

### Redescription (Figs. 174–180)

Body proper flattened dorsoventrally, elongate, usually with nearly parallel lateral margins; cephalic region broad, with three bilateral pairs of head organs, moderately developed bilateral and terminal cephalic lobes; peduncle narrow, tapered posteriorly, with pair of peduncular glands. Two pairs of eyespots; members often poorly developed or dissociated, lacking lenses; chromatic granules minute, variable in shape, scattered throughout cephalic region. Mouth subterminal, midventral, at level of head organs; pharynx subspherical, with posterior indentation; moderately long esophagus bifurcating to form intestinal ceca; intestinal ceca lacking diverticula, confluent posterior to testis. Haptor ellipsoidal, large bilateral lobes containing hook pairs 2–4, 6, 7. Ventral anchor robust, with long point, straight shaft, enlarged base lacking deep root and having short knob-like superficial root; crescent-shaped sclerite present (see Lim & Gibson [52]). Dorsal anchor with long delicate point, straight shaft having distal constriction at union with point, base lacking deep root and having long superficial root. Ventral bar saddle shaped, with two submedial digitiform processes directed posteriorly. Dorsal bar rod shaped, with deeply bifurcated ends and spur-like posteromedial process. Hook distribution normal; each hook comprising C-shaped hooklet and delicate shank comprised of single subunit; FH loop about 3/4 shank length. Testis ovate; vas deferens, seminal vesicle not observed. Prostatic glands conspicuous in anterior trunk dorsal to copulatory complex; two elongate prostatic vesicles crossing each other. MCO elongate, comprised of tubular shaft arising from small inverted-cup-shaped base; shaft with small delicate terminal flange and inconspicuous sheath enveloping distal end; accessory piece absent. Allantoid germarium folded upon itself; intercecal oviduct directed sinistrally around seminal receptacle; uterus not observed; two groups of Mehlis’ glands, one lying along each side of germarium. Vaginal pore dextromarginal; vagina unsclerotized, with thick distal wall; vaginal canal delicate, expanding to form large pyriform seminal receptacle when sperm (spermatophore?) present; seminal receptacle with proximal encircling ridge. Vitellarium dense, coextensive with intestinal ceca; bilateral vitelline ducts immediately anterior to seminal receptacle. Egg not observed.

**Figures 174–180:**
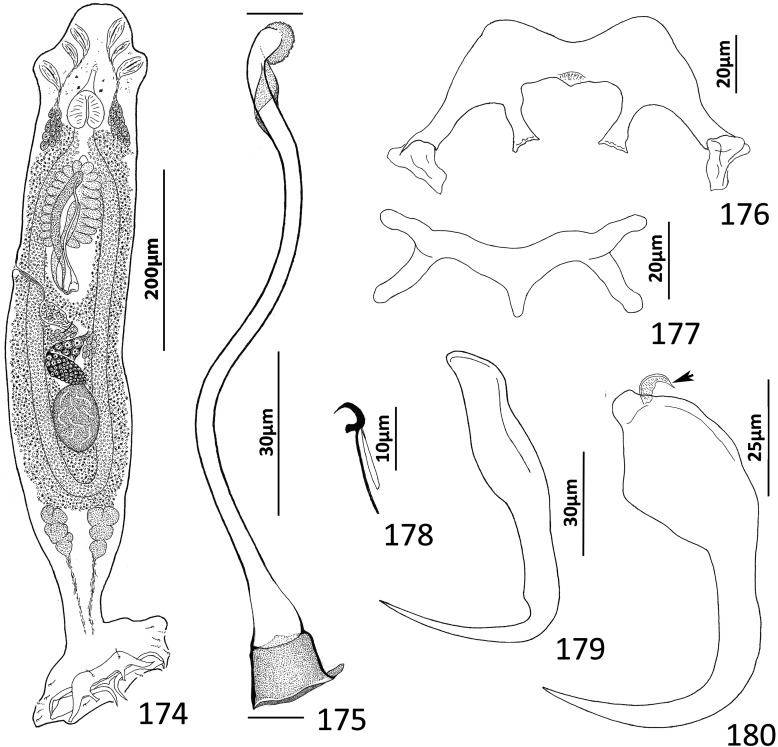
*Triacanthinella falcanalis* (Young, 1968) Lim & Gibson, 2008 from the black-flag tripodfish, *Tripodichthys angustifrons*, Triacanthidae. 174, Whole mount (ventral view, composite); 175, Male copulatory organ (ventral view); 176, Ventral bar; 177, Dorsal bar; 178, Hook; 179, Dorsal anchor; 180, Ventral anchor (arrow indicates the crescent sclerite presented by Lim & Gibson [52]). Parallel lines on Figure 175 indicate the limits of the dimension measured.

Measurements (respective measurements from Young [95] follow those of present specimens in brackets): Body 433–828 (684; *n* = 25) [572–1,380] long; greatest width 92–166 (126; *n* = 25) [82–240]. Haptor 113–191 (148; *n* = 23) wide. Ventral anchor 51–86 (67; *n* = 13) [57–65] long; dorsal anchor 47–71 (60; *n* = 13) [51–58] long. Ventral bar 78–112 (95; *n* = 13) [46–68] long; dorsal bar 56–84 (70; *n* = 13) [43–41, *sic*] long. Hook 15–20 (17; *n* = 20) long. Pharynx 30–60 (41; *n* = 24) long, 28–48 (39; *n* = 24) wide. Testis 30–105 (76; *n* = 21) long, 30–79 (56; *n* = 21) wide. Male copulatory organ 76–170 (121; *n* = 9) [81–154] long. Germarium 28–71 (53; *n* = 21) long, 30–52 (42; *n* = 21) wide.

### Remarks

This species was described by Young [95] as *Haliotrema falcanalis* Young, 1968 from the gills of the black-flag tripodfish occurring in Moreton Bay. In his revision of *Haliotrema*, Young [95] proposed six species groups to include the then known species assigned to the genus; he placed *H. falcanalis* in Species Group 1 along with twelve other species occurring on the gills of fishes belonging to five perciform families (the Acanthuridae, Zanclidae, Balistidae, Triacanthidae, and Apogonidae). Species Group 1 was diagnosed in part with the following features: dorsal anchors with shafts at 90° to the plane of the basal roots; absence of peduncular glands; the MCO directed anteriorly and lacking an accessory piece; and presence of a single prostatic reservoir. Several of Young’s [95] species groups were later shown to represent monophyletic genera, including his Species Group 2 (now represented by *Euryhaliotrema* and *Haliotrematoides* Kritsky, Yang, & Sun, 2009), Species Group 3 (now *Ligophorus*), Species Group 4 (likely representing *Haliotrema* [*sensu stricto*]), and Species Group 6 (now *Lethrinitrema*) (see [13, 30, 35, 46, 54]).

That Young’s species group 1 was unnatural was suggested by Bychowsky & Nagibina [4], who stated in a footnote at the end of their paper that *H. falcanalis* was a member of *Triacanthinella*. Bychowsky & Nagibina [4] did not provide a new binomen for *H. falcanalis*, and as a result, formal transfer of the species to *Triacanthinella* was not accomplished by their statement. Without comment on the footnote, Lim & Gibson [52] formally transferred the species to *Triacanthinella* as *T. falcanalis* (Young, 1968) Lim & Gibson, 2008.

*Triacanthinella falcanalis* was redescribed herein to augment the original description [95]. Current specimens revealed the presence of two prostatic reservoirs paralleling the copulatory complex (one reservoir reported in the original description); absence of the 90° flexion of the dorsal anchor (present in the original description, apparently a result of an artifact introduce by coverslip pressure during mounting on slides); and presence of cephalic glands flanking the pharynx (not observed by Young [95]). In his Table 1, Young [95] states that species assigned to Species Group 1 lack “peduncular gland cells” but shows their presence in his figure 3a of the whole mount of *H. falcanalis*. A pair of peduncular gland cells were also present in specimens collected for the present study.

## On *Pleuronectitrema kuwaitense* Kritsky & Sey n. sp., a new species from the Persian Gulf off Kuwait

The primary objective of the present paper was to report on the diversity of dactylogyrids (Monogenoidea) occurring on the marine fishes of Moreton Bay, and the proposal of *Pleuronectitrema* gen. n. was deemed necessary for *Pleuronectitrema spirula* n. sp. found infecting the gill lamellae of the large toothed flounder, *Pseudorhombus arsius* in the bay (see above for the diagnosis of the genus and description of *P. spirula*). In 1996, a similar species was collected from the same host from the Persian Gulf off Kuwait by Dr. Otto Sey, the specimens of which were forwarded shortly thereafter to the author for study. These specimens were initially recognized as representing a new genus, but to propose the taxon at that time would have rendered it monotypic as no other dactylogyrid species was sufficiently described that could also have been included in the genus. The proposal of *Pleuronectitrema*, with *P. spirula* n. sp. as its type species, now provides a generic-level taxon for the Kuwaiti specimens, which are named and described below as *Pleuronectitrema kuwaitense* n. sp. Including *P. kuwaitense* n. sp. in the present paper provides additional support for the validity of the new genus.

## *Pleuronectitrema kuwaitense* Kritsky & Sey n. sp.

urn:lsid:zoobank.org:act:CB7E08C3-8DF6-4FAC-B06D-7DD3759A843F

Type host: Large toothed flounder *Pseudorhombus arsius* (Hamilton), Pleuronectiformes, Paralichthyidae.

Type locality: Persian Gulf off Kuwait, 16 October 1996.

Infection site: Gill lamellae.

Specimens studied: Holotype, USNM 1692910; 9 paratypes, USNM 1692911-1692914, HWML 217010.

Etymology: The specific name reflects the country from which the parasite was collected.

### Description (Figs. 181–187)

Body slender, elongate, somewhat flattened dorsoventrally. Cephalic region broad, with numerous prepharyngeal glandular bodies, poorly developed cephalic lobes; three pairs of head organs. Two pairs of eyespots often absent (absent in holotype); lenses absent; chromatic granules minute, generally ovate; accessory granules usually present throughout cephalic region. Pharynx subspherical to ovate. Peduncle elongate, slightly tapered posteriorly. Haptor subhexagonal in dorsoventral view. Ventral anchor with large base having short superficial root and minimally developed deep root, evenly curved shaft, short point extending past level of tip of superficial root. Dorsal anchor comparatively delicate, with moderately long superficial root, incipient deep root, arcing shaft, short point extending to level of tip of superficial root. Ventral bar broad, flat, with bilateral arms directed posterolaterally. Dorsal bar gently arced, with tips recurved posterolaterally. Hooks delicate; each with upright acute thumb, undilated shank; FH loop nearly shank length. Testis elongate ovate; proximal portion of vas deferens not observed, distal portion dilating to form fusiform seminal vesicle; seminal vesicle extending diagonally from left side of trunk toward base of MCO. Prostates not observed; single vesicle (apparently a prostatic reservoir) lying posterior to base of MCO. MCO a loosely coiled tubular shaft with about 1½ counterclockwise rings arising from expanded base; shaft with thick proximal wall giving rise distally to sheath enclosing distal portion of shaft. Germarium pyriform; oötype, Mehlis’ glands, uterus not observed. Vaginal pore midventral, difficult to observe; Vaginal canal apparently short. Seminal receptacle variable, lying ventral to anterior end of germarium. Vitellarium coextensive with intestinal ceca; bilateral vitelline ducts extend toward body midline where they fuse anterodorsal to seminal receptacle. Egg not observed.

**Figures 181–187:**
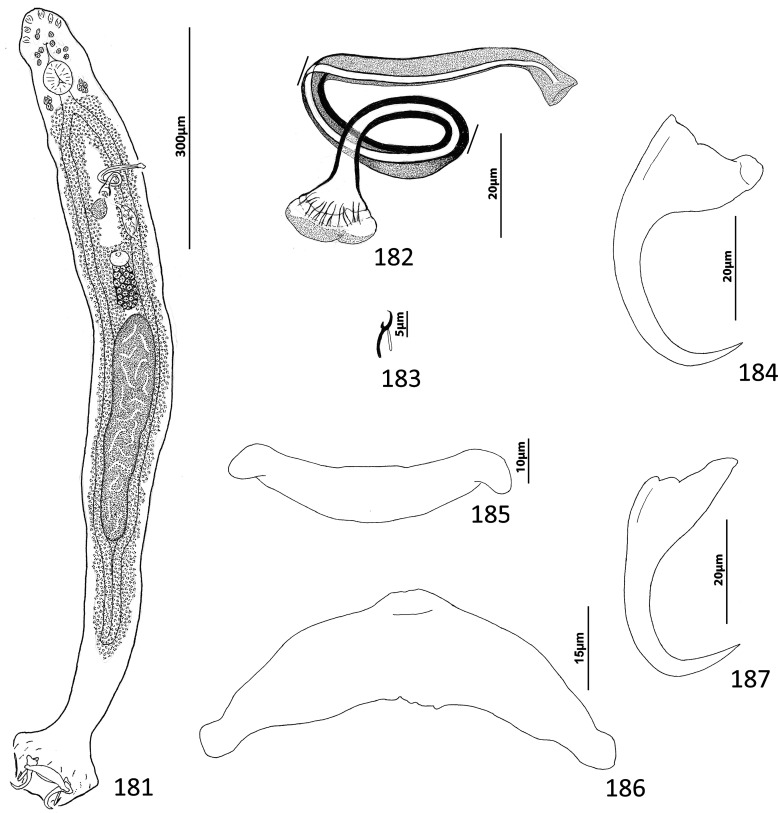
*Pleuronectitrema kuwaitense* Kritsky & Sey n. sp. from the large toothed flounder, *Pseudorhombus arsius*, Paralichthyidae. 181, Whole mount (ventral view, composite); 182, Male copulatory organ (ventral view); 183, Hook; 184, Ventral anchor; 185, Dorsal bar; 186, Ventral bar; 187, Dorsal anchor. Measurements of the length of the MCO (Fig. 182) are the actual lengths obtained using a calibrated curvimeter on drawings made using a camera lucida; parallel lines on Fig. 182 indicate the measured limits of the ring diameter.

Measurements: Body 1,010–1,160 (1,080; n =3) long; greatest width (excluding haptor) 135–147 (141; *n* = 3). Haptor 141–147 (145; *n* = 11) wide. Ventral anchor 49–52 (51; *n* = 6) long; dorsal anchor 43–50 (46; *n* = 6) long. Ventral bar 72–85 (79; *n* = 6) long; dorsal bar 50–61 (55; *n* = 6) long. Hook 10–12 (11; *n* = 18) long. Pharynx 43–51 (47; *n* = 3) long, 40–48 (45; *n* = 3) wide. Testis 230–334 (271; *n* = 3) long, 58–84 (71; *n* = 3) wide. Male copulatory organ 125–155 (140; *n* = 7) long (actual length); ring diameter 21–35 (29; *n* = 7). Germarium 64–105 (84; *n* = 3) long, 41–49 (45; *n* = 3) wide.

### Remarks

The specimens on which this species was based were collected by Dr. Otto Sey from the large toothed flounder from the Persian Gulf off Kuwait during 1996. *Pleuronectitrema kuwaitense* n. sp. differs from *Pleuronectitrema spirula* n. sp. by having 1) a larger diameter of the rings of the MCO (21–35 in *P. kuwaitense*, 12–16 in *P. spirula*), 2) anchors with comparatively longer shafts, and 3) a large flat ventral bar (ventral bar rod shaped in *P. spirula*). *Pleuronectitrema kuwaitense* most closely resembles *P. youngi* (Venkatanarasaiah, 1984) n. comb. by having a similarly shaped MCO. It differs from *P. youngi* by its broad ventral bar, ventral anchors with comparatively long shafts, and dorsal anchors with shorter superficial roots. Venkatanarasaiah [84] apparently erroneously depicted the hooks of *P. youngi* as having protruding and blunt thumbs (thumbs of the hooks of *P. kuwaitense* are upright and acute).

## Conclusion

Over a period of about ten days during January 2016, 51 species of Dactylogyridae were collected from 278 marine fishes (representing 73 species) examined from Moreton Bay. These dactylogyrids included 28 new species and represented 22 genera, of which three were newly proposed. Prior to the published studies emanating from the January survey, only 14 previously described species of Dactylogyridae had been recorded from Moreton Bay. That more than 1,100 species of marine fishes occur within the bay [25] and that only 60 species of Dactylogyridae are currently known from fewer than 10% of the potential hosts present (Table 1), suggest that many dactylogyrid species remain to be documented from the waters of Moreton Bay.
